# Behaviour change interventions to promote health and well-being among older migrants: A systematic review

**DOI:** 10.1371/journal.pone.0269778

**Published:** 2022-06-16

**Authors:** Warsha Jagroep, Jane M. Cramm, Semiha Denktaș, Anna P. Nieboer

**Affiliations:** 1 Department of Socio-Medical Sciences, Erasmus School of Health Policy & Management, Erasmus University Rotterdam, Rotterdam, The Netherlands; 2 Department of Psychology, Education and Child Studies, Erasmus School of Social and Behavioural Sciences, Erasmus University Rotterdam, Rotterdam, The Netherlands; PLOS: Public Library of Science, UNITED KINGDOM

## Abstract

**Background:**

Whether behaviour change interventions are effective for the maintenance of older migrants’ health and well-being is uncertain. A systematic review was conducted to assess evidence for the capacity of behaviour change techniques (BCTs) to promote the health and well-being of older migrants.

**Methods:**

Electronic databases (Cochrane CENTRAL, Embase, Ovid MEDLINE and Web of Science) were searched systematically to identify relevant randomised controlled trials, pre–post studies and quasi-experimental studies published before March 2021. Additional articles were identified through citation tracking. Studies examining BCTs used to promote the health and/or well-being of older migrants were eligible. Two independent reviewers used the Behaviour Change Technique Taxonomy version 1 to extract data on BCTs. Data on intervention functions (IFs) and cultural adaption strategies were also extracted. Intervention contents (BCTs, IFs, culture adaption strategies) were compared across effective and ineffective interventions according to health and well-being outcome clusters (anthropometrics, health behaviour, physical functioning, mental health and cognitive functioning, social functioning and generic health and well-being).

**Results:**

Forty-three studies (23 randomised controlled trials, 13 pre–post studies and 7 quasi-experimental studies) reporting on 39 interventions met the inclusion criteria. Thirteen BCTs were identified as promising for at least one outcome cluster: goal-setting (behaviour), problem-solving, behavioural contract, self-monitoring of behaviour, social support (unspecified), instruction on how to perform the behaviour, information about health consequences, information about social and environmental consequences, demonstration of the behaviour, social comparison, behavioural practice/rehearsal, generalisation of a target behaviour and addition of objects to the environment. Three BCTs (instruction on how to perform the behaviour, demonstration of the behaviour, and social comparison) and two IFs (modelling and training) were identified as promising for all outcome clusters.

**Conclusions:**

Thirteen distinct BCTs are promising for use in future interventions to optimise health and well-being among older migrants. Future research should focus on the effectiveness of these BCTs (combinations) in various contexts and among different subgroups of older migrants, as well as the mechanisms through which they act. Given the scarcity of interventions in which cultural adaption has been taken into account, future behavioural change interventions should consider cultural appropriateness for various older migrant (sub)groups.

**Trial registration:**

PROSPERO CRD42018112859.

## Introduction

Globally migration occurs due to a range of factors such as political unrest, unemployment, colonial factors and displacement related to conflict [1]. Although migration is not a recent phenomenon, is has been an important part of human history. According to the Population Division of the United Nations, the global number of international migrants increased by 60 million between 2010 and 2020 [2]. Older adults are overrepresented among international migrants, compared to the total population; in 2020 globally 12% of international migrants were at least 65 years old, compared to 9% of the total population [2].

“Migrant” is an umbrella term that refers to “a person who moves away from his or her place of usual residence, whether within a country or across an international border, temporarily or permanently” ([3], p. 132). Research in the interdisciplinary field of aging and migration generally differentiates groups of migrants, such as guest workers (e.g., Turks and Moroccans in Western Europe), people from former colonies (e.g., Indians and Pakistanis in the UK, Indonesian and Surinamese in the Netherlands), and labor migrants (e.g., Mexicans and Caribbeans in the US) [4, 5]. Older migrants in these groups have the common characteristics of having migrated at relatively young ages, having spent much of their working lives in their host countries, and identification as first-generation migrants (people born in a country other than that of their country of residence) [6–9].

When people migrate, they settle down in a new culture, developing “migrant identities.” These identities are aspects of people’s social identities derived from the sense of belonging to a particular group, culture, and environment [10]. Positive migrant identities buffer against the distress experienced by migrants and seem to be invariant across ethnicities [11–14]. Migrant identities are essential for the well-being of people experiencing other cultures [15]; indeed, they seem to be associated positively with health and well-being [16–18]. They can be seen as an aspect of acculturation, which involves physical, psychological, cultural, and social changes (e.g., learning a new language, establishing new social connections, shifting old cultural expectations) as people adjust to the cultures of their host countries [19]. Individuals create migrant identities in societies in four ways: 1) through strong identification with both groups, indicative of integration; 2) through identification with neither group, suggesting marginalization; 3) through exclusive identification with the majority culture, indicating assimilation; and 4) through identification with only the minority group, reflecting separation [20]. For older migrants, this process involves cross-cultural adjustment and dealing with aging in a foreign country. A majority of migrants experience acculturative stress and difficulties with their identities due to the pressure to assimilate while maintaining cultural roots; identification with two different groups may be challenging for ethnic minority group members because of conflicts in attitudes, values, and behaviours [21, 22]. Irrespective of how migrants identify themselves, their home and host cultures seem to have impacts on their lives, depending on the context [23]. Given that culture varies across groups and within the same group and individual, cultural appropriateness should be considered when migrants are involved [24].

The disease risk profiles of migrant and native populations differ, sometimes in favor of, but usually to the disadvantage of migrants. Well-documented examples are the greater prevalence of coronary disease among people originating from the South Asian subcontinent [25], depression among labor migrants from Morocco and Turkey [26], and stroke among people originating from Africa [27]. Mental disorders are common in a large share of migrant populations [28, 29]. This manifestation has been related to the feelings of rejection in host countries, social exclusion, and discrimination that these populations may face [6, 30–33]. Perceived discrimination also seems to negatively affect migrants’ health and well-being [34–39], and has been related to higher levels of stress and unhealthy behaviours [33, 40, 41]. Older migrants are especially vulnerable, given that they tend to have more chronic diseases, lower levels of self-rated health and functioning, more limitations in daily activities, and higher rates of depressive symptoms compared with their native counterparts [42–50], which might have an impact on their quality of life [51]. The maintenance of healthy behaviours such as physical activity (PA) and healthy eating is known to be beneficial for physical health, mental health, and well-being [52–57].

Although migrants are expected to differ on some aspects, depending on their country of origin, there are also similarities for the migrant population in general. Older migrants are more likely than their native counterparts to be disadvantaged in terms of socio-economic status (SES) due to lower educational levels, un/under-employment and, often, responsibilities to relatives abroad [58–61]. Low SES has been shown to result in poorer health [62], well-being [63, 64] and unhealthy behaviours [65, 66], and thus less overall health [67].

On arrival in a variety of countries, migrants are widely acknowledged to have an initial health advantage over the native population, known as the “healthy migrant effect” [68–75]. A vast body of empirical literature, however, shows that the initial health advantage diminishes over time [50, 76]. Migrants acquire disadvantages over the life course, both early life conditions in the country of origin, as well as exposure to challenging situations in the country of designation in terms of poor economic and social conditions. These economic and social conditions are further enforced by cultural and language barriers, homesickness, discrimination and stigmatization [77–79], which erases the initial health advantage and creates a decline over time of migrants’ health status [58].

Next, for older migrants ageing takes place in a second language environment. This poses extra cultural, social, and health-related challenges when mastery of the second language is low. Inadequate health literacy [80, 81], language differences [82–84] and, sociocultural factors [85, 86] may be informal barriers to physical and mental health services access. Low language proficiency also affects individuals’ health behaviours [87] because the ability to find and understand health information is associated with the ability to make appropriate health-related decisions [88].

Finally, accessing physical and mental health services is challenging for older populations in general [89–91], however, research indicates that it is more challenging for older migrants due to barriers associated with language, discrimination, health beliefs, the lack of culturally appropriate programs, knowledge of the health care system, and awareness of available health services [47, 92–97]. These factors may result in the underutilization of essential health services [98–100], eventually leading to increase in illness, lower levels of well-being, multimorbidity, disability, and mortality [82, 101–103]. Given these commonalities, this systematic review focused on the global population of older migrants.

People’s health and well-being depend heavily, but not solely, on health behaviours; for instance, PA results in improved cardiovascular health, lower blood pressure, increased muscular strength, decreased depression and improved quality of life [104]. A healthy diet helps to prevent many diseases, such as diabetes [105], coronary heart disease [106] and cancer [107]. Social behaviours, such as social participation and social activities, also improve health and well-being [108–111]. These examples illustrate how health behaviours are relevant to health and well-being, and in turn depend on behaviour choices [112]. These choices, such as being (more) active and having a healthy diet, are not always easily accomplished or maintained. Moreover, engagement in healthy behaviours differs across migrant groups, due for example to cultural factors such as PA patterns and dietary habits, and individual factors such as language proficiency [113–115]. The challenge is to find appropriate means of supporting older migrants’ adoption of healthy behaviours.

Behaviour change interventions (BCIs) have received considerable attention, given their potential to promote healthy behaviours, such as the adoption of a healthy diet [116, 117], adequate PA [118] and social activity [119]. BCIs can be defined as coordinated sets of activities designed to change specific behaviour patterns [120], and have the potential to improve health and well-being [121]. BCIs have observable, replicable and irreducible components designed to alter or redirect behaviour, known as behaviour change techniques (BCTs) [120]. BCTs are relevant across behaviours and outcomes, as they are not specifically applicable to single outcome measures. The identification of BCTs aids the assessment of the effectiveness of intervention components targeting several outcome measures. Several reviews have examined associations between BCTs and intervention effects, identifying various effects [116–119, 122]. Increasing numbers of BCTs are not necessarily associated with better outcomes [116], but combinations of BCTs might increase the effectiveness of interventions [122]. Yet, no existing evidence indicates which BCTs optimise older migrants’ health behaviours and, consequently, their health and well-being.

A useful start in addressing this issue and identifying potentially effective components is to specify the BCTs used in interventions. BCT taxonomies are developed with the aim of establishing a sound basis for description of the procedures involved in interventions, without additional assumptions about BCTs, and ultimately of guiding the development of future behaviour change interventions [123]. Michie and colleagues [124] developed the Behaviour Change Technique Taxonomy version 1 (BCTTv1), a comprehensive taxonomy which describes distinct techniques that may be used to change behaviour, together with nine individual functions that any intervention may provide [120]. Michie and colleagues propose that the classification of distinct BCTs and functions within interventions enables determination of how interventions operate and thus of which components might be integrated into new and more effective interventions [120, 124, 125].

Previous research has indicated that intervention effectiveness may be improved by tailoring programmes to the relevant populations (e.g. by taking culture into account) [126–128], but current systematic reviews focus mainly on the health and well-being of the general older population, and not specifically on older migrants [129–133]. Older migrants comprise a vulnerable group in society in terms of the maintenance of health and well-being. Behaviour change interventions have the potential to positively affect health behaviours. The identification of intervention components such as BCTs that can effectively change behaviours can guide future intervention development, thereby improving the health and well-being of vulnerable groups such as older migrants. To our knowledge, no systematic review to date has explored BCIs targeting the health and/or well-being of older migrants; thus, we conducted this review of existing empirical research on the topic. Its aim was to identify promising BCTs that are components of effective BCIs that promote the health and/or well-being of older migrants, with consideration of the cultural adaption of interventions. The findings will facilitate the development of effective BCIs for this population.

## Methods

This systematic review was registered with the International Prospective Register of Systematic Reviews (PROSPERO, registration number CRD4201811285), and was conducted and reported following the Preferred Reporting Item for Systematic Reviews and Meta-Analyses (PRISMA) statement (S1 Table) [134].

The definition of older individuals varies among countries [135] and between natives and migrants [136]. In most high-income countries, the cut-off of 65 years is used to demarcate older age, but this threshold is not suitable for older migrants. In most Asian countries, people aged ≥ 45 years are considered to be old. In general, migrants report ‘feeling old’ at younger ages relative to their native counterparts [137], which is related to their hard lives and work, and low educational levels. To maintain consistency with international studies [138–140], we used the cut-off age of 45 years to define older individuals.

For the present review, the definition of migrant was based on that provided by the International Organization for Migration: ‘any person who is moving or has moved across an international border or within a State away from his/her habitual place of residence, regardless of the person’s legal status, whether the movement is voluntary or involuntary, what the causes for the movement are, or what the length of stay is’ [141]. As this review focused on international migrants, we did not consider individuals who migrated within states.

### Search strategy

We conducted a systematic search of reports on BCIs implemented with older migrants. Studies were identified initially by searches of the Cochrane CENTRAL, Embase, Ovid MEDLINE and Web of Science electronic databases from inception to March 2021 using keywords referring to BCIs, older migrants (age ≥ 45 years), health and well-being. Details of the searches are provided in S2 Table. Additionally, references cited in identified reviews were screened for eligibility. No limitation on the date of publication was imposed.

### Data collection and analysis

#### Selection of studies

Studies that were eligible for inclusion met the following criteria: 1) focus on BCIs to promote health and/or well-being; 2) targeting of individuals aged 45 years and older; 3) inclusion of migrants; 4) randomised controlled trial, pre–post study, or quasi-experimental design; and 5) written in English.

#### Data extraction and management

All identified articles were downloaded to the reference management software Endnote X6.0.1. Duplicate reports were excluded, and the titles and abstracts of the remaining articles were assessed for relevance. The inclusion criteria were then applied to exclude ineligible articles. Additionally, identified reviews were screened for potentially relevant references. The full texts of the eligible articles were then retrieved and subjected to full review. Two reviewers (JMC and RWJ) independently performed study selection. Discrepancies regarding eligibility were resolved by discussion and consensus. The following data were extracted from the final set of selected articles and entered into an Excel spreadsheet (Microsoft Office Professional Plus 2013): author(s), title, year of publication, aim, ethnicity of participants (definition of migrant), age range, study design, data collection/follow-up (method and period), loss to follow-up (number and reasons), intervention details [country, setting, content, function, BCT, level (individual or group), cultural adaption, intensity], behaviour change theory/model used, method(s) used to asses health and well-being and corresponding results. *P* values for mean changes between baseline and follow-up(s) were extracted, with effect sizes when available. Effects were interpreted as small (Cohen’s *d* ≥ 0.2), medium (*d* ≥ 0.5) and large (*d* ≥ 0.8) [142].

#### Study quality and risk of bias assessment

The same two reviewers who assessed study eligibility (JMC and RWJ) assessed the methodological quality of the studies using the seven domains (random sequence generation, random allocation concealment, blinding of participants and personnel, blinding of outcome assessment, incomplete outcome data, selection outcome reporting and other sources of bias) provided in the Cochrane Handbook [143] for randomised controlled trials (S3 Table). Each domain was rated according to the risk of bias (high, low or unclear). Studies were considered to be highly susceptible to bias when two or more of the seven domains showed susceptibility to bias, three or more domains had unclear risks, or one domain showed susceptibility to bias and two domains had unclear risks. Eleven studies showed susceptibility to bias [144–154], three studies had a moderate risk of bias [155–157] and nine studies had a low risk of bias [158–166].

The Newcastle-Ottawa Scale (NOS) [167] for cohort studies was used to assess the quality of non-randomised trials (S4 Table). Points were allocated for the three domains of selection, comparability and outcome (maximum, 9 points). The risk of bias was categorised as high (0–3 points), moderate (4–6 points) or low (7–9 points). The strength of the evidence for each intervention was assessed using predefined criteria adapted from the Center for Evidence-Based Medicine’s levels of evidence [168] (S3 and S4 Tables). Eleven studies had a moderate risk of bias [169–179] and eight studies had a low risk of bias [180–187].

#### Intervention functions

Descriptions from the behaviour change wheel [120] were used to code each intervention as serving one or more of the following nine functions: 1) coercion (creating the expectation of punishment or cost), 2) education (increasing knowledge or understanding), 3) enablement [increasing means/reducing barriers to increase capability (beyond education and training) or opportunity (beyond environmental restructuring)], 4) environmental restructuring (changing the physical or social context), 5) incentivisation (creating the expectation of reward), 6) modelling (providing an example for people to aspire to or imitate), 7) persuasion (using communication to induce positive or negative feelings or stimulate action), 8) restriction [using rules to reduce the opportunity to engage in the target behaviour (or to increase the target behaviour by reducing the opportunity to engage in competing behaviours)] and 9) training (imparting skills). For example, an intervention involving the provision of information to promote healthy eating was coded as having an ‘education’ function. Each intervention could involve more than one of the nine functions.

#### Cultural adaption

To identify culturally adapted interventions, Kreuter and colleagues’ [188] work describing strategies to promote culturally appropriate health promotion programmes and materials was used. Five categories were distinguished: 1) peripheral strategies that improve programme/study materials’ visual appeal to the target population (i.e. by using certain colours, images or fonts), 2) evidential strategies that enhance the perceived relevance of a health issue for a given group by raising awareness and providing facts on the importance of a health condition for that group, 3) linguistic strategies to improve the accessibility of programmes/materials by providing them in the dominant or native language of the target population, 4) constituents-involving strategies that draw directly on the experience of members of the target population (i.e. by hiring indigenous staff members, involving community members in programme development and delivery) and 5) socio-cultural strategies by which health-related issues are discussed in the context of broader social and/or cultural values and characteristics of the target population.

#### BCTs coding and analysis

The BCTTv1 provides consensus definitions and labels for 93 distinct BCTs organised hierarchically into 16 clusters: 1) goals and planning, 2) feedback and monitoring, 3) social support, 4) shaping knowledge, 5) natural consequences, 6) comparison of behaviour, 7) associations, 8) repetition and substitution, 9) comparison of outcomes, 10) reward and threat, 11) regulation, 12) antecedents, 13) identity, 14) scheduled consequences, 15) self-belief and 16) covert learning [124]. The taxonomy also includes detailed coding instructions enabling the identification and precise description of technical intervention components that elicit behaviour changes (e.g. physical activity, diet). According to its authors, the BCTTv1 is a trustworthy tool for the extraction of details about intervention content, and the identification and synthesis of distinct and replicable (combinations of) potentially active ingredients related to effectiveness [124]. The BCTTv1 was developed for the organisation of information about behaviour change interventions, rather than to illustrate behaviour in a real-time context [189]. In addition, it enables the investigation of how other factors, such as the mode of delivery, intervention intensity, target behaviour and target population, may make BCTs more or less effective [125]. The BCTTv1 guides the characterization of intervention content to facilitate intervention implementation, delivery and evaluation, with the synthesis of evidence at the BCT level [116–119]. Trained coders can apply the BCTTv1 to identify BCTs from intervention descriptions reliably (in consensus with each other and over time) and validly (as assessed by agreement with experienced coder consensus) [190]. BCTTv1 codes were assigned to intervention components. BCTs in intervention and control groups were identified separately, and BCTs applied exclusively in the intervention groups were extracted. This approach was used to explain difference in effects, as described by Peters and colleagues [191]. Online training in BCTTv1 use was completed [192], to ensure consistency in data recording, BCT data extraction was duplicated independently (by JMC and RWJ) at a level of 10% based on the most comprehensive published intervention descriptions. Freely available published protocols and full manuals were used for the coding procedure when available. Any disagreement in coding was resolved through discussion to reach consensus. Following BCTTv1 coding principles, we extracted BCTs that were definitely (coded ++) or probably (coded +) present to capture all relevant BCTs. For example, when an intervention involved participants’ recording of their food intake, the ‘self-monitoring of behaviour’ BCT was coded as probably present (+). When the intervention manual indicated that participants were asked to record and review their food diaries each week, this BCT was coded as definitely present (++).

As no intervention assessed in the included studies involved the use of a single BCT, we report only on the effectiveness of BCT combinations.

#### Analysis

Two analyses were completed. First, intervention components covering intervention function, cultural awareness and BCTs, of the included interventions were described. Second, the effectiveness of interventions and links between components and effectiveness were estimated.

Following Gardner and colleagues [121], each extracted outcome variable was classified inductively into one of six outcome clusters: anthropometrics (i.e. weight, blood pressure), health behaviour (i.e. PA, fruit and vegetable intake, social activity), physical functioning (i.e. functional status, activities of daily living, physical impairment), mental health and cognitive functioning (i.e. depressive symptoms, recall), social functioning (i.e. social support, loneliness) and generic health and well-being involving indicators not captured by other clusters (i.e. health-related quality of life, vitality, pain).

Intervention effectiveness was evaluated for each outcome cluster according to the presence of a significant (p < 0.05) change in the intervention group. Indices of potential (IPs) for intervention components (percentages with evidence of effectiveness) were computed for each outcome cluster, following Gardner and colleagues [121]. IPs were calculated only for components used in four or more interventions to avoid over-interpretation of scant data. Intervention components with IPs > 50% (indicating that they were present in more effective than ineffective interventions) were deemed ‘promising’, and those with IPs ≤ 50% were deemed ‘not promising’.

The effectiveness of lifestyle interventions is commonly assessed after 3, 6, or 12 months [193]. The impact of intervention content (i.e. in terms of the behaviour targeted, intervention function, cultural awareness and BCTs) on effectiveness was assessed for all included studies and separately for studies providing ≥3 months follow-up data as opposed to shorter than 3 months.

## Results

**Fig 1:**
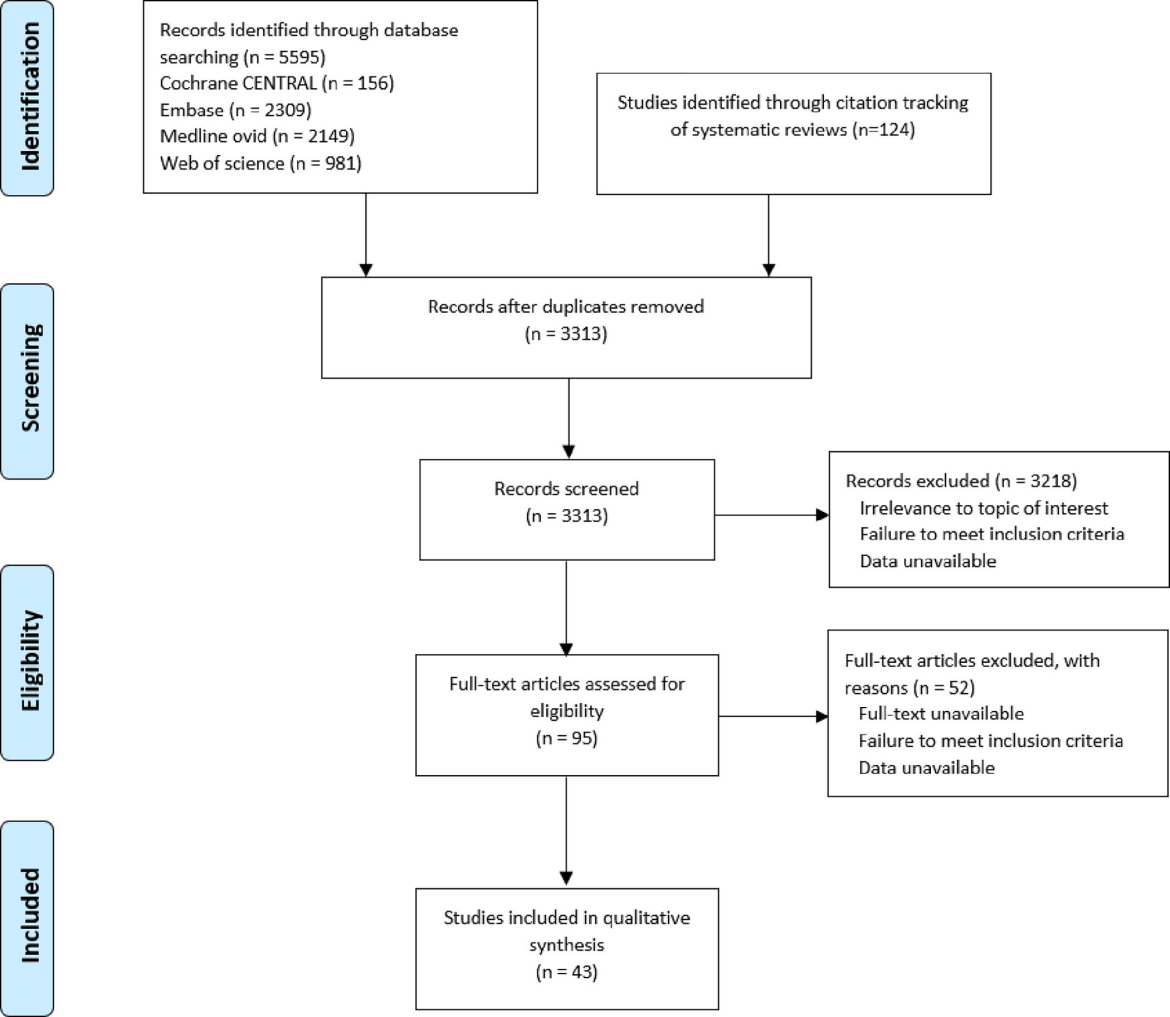
Flowchart of study selection. PRISMA Flowchart for the identification, screening, eligibility and inclusion of studies [134].

The search for articles related to BCIs promoting health and well-being among older migrants yielded 3313 records, from which 95 full-text articles were retrieved. After application of the eligibility criteria, 43 studies were deemed eligible for inclusion (Fig 1).

### Description of the included studies

Tables 1 and 2 summarise study and intervention characteristics; further study details are provided in S5 Table. The reviewed studies were randomised controlled trials (*n* = 17), cluster-randomised controlled trials (*n* = 2), parallel design RCT (*n* = 2), two-group randomised controlled trials (*n* = 1), three-group randomised controlled trial (*n* = 1), pre–post studies (*n* = 13) and quasi-experimental studies (*n* = 7). Three countries were represented, including Canada (*n* = 3), the Netherlands (*n* = 1) and the United States (*n* = 39). The studies included 20–781 individuals, with most of the studies (74%) using a cut-off age of 65 or 70 years. Across all studies, the most common races/ethnicities were Black/African American, Hispanic/Latino, Asian, Chinese and Korean American. Twenty-nine studies [144, 149, 151–156, 158, 161–166, 170, 172–183, 194] involved solely older migrants and four-teen studies [145–148, 150, 157, 159, 160, 169, 171, 184–187] involved older migrants and natives. With the exception of two studies [159, 169], more than 50% of participants in the latter studies were migrants. Participants’ migration backgrounds were defined by birth in a foreign country, proficiency in a language other than the native language of the country in which the intervention was conducted, and self-identification as a migrant. The authors of 30 articles did not mention how participants’ migration backgrounds were defined.

**Table 1:** Summary of intervention characteristics (*n* = 39).

Intervention characteristics		Number of interventions (*n* = 39) (%)
Number of behaviours targeted		
	One behaviour	28 (72%)
	Two behaviours	11 (28%)
Specific behaviours targeted		
	Physical activity	31 (79%)
	Healthy diet	10 (26%)
	Social functioning	6 (15%)
	Blood pressure management	1 (3%)
	Depression management	1 (3%)
	Health management	2 (5%)
Intervention functions^#^		
	Education	19 (49%)
	Enablement	19 (49%)
	Environmental restructuring	8 (21%)
	Modeling	14 (36%)
	Persuasion	15 (38%)
	Training	16 (41%)
Cultural adaptive interventions^¥^		24 (62%)
	Constituents-involving strategies	4 (17%)
	Linguistic strategies	19 (79%)
	Peripheral strategies	2 (8%)
	Sociocultural strategies	11 (46%)
Setting		
	Community based	5 (13%)
	Community center	8 (21%)
	Home based	6 (15%)
	Local church	5 (13%)
	Public elementary school	1 (3%)
	Senior center	10 (26%)
	Urban hospital	1 (3%)
Delivered by		
	Exercise expert	5 (13%)
	Health care professional	9 (23%)
	Older adult volunteer	1 (3%)
	Peer educator	3 (8%)
	Researcher	2 (5%)
	Trained facilitator	11 (28%)
	Not mentioned	8 (21%)
Evidence of effectiveness, by outcome cluster		
	Anthropometrics (*n* = 16)	Effective *n* = 12
		Not effective *n* = 4
	Behaviour (*n* = 17)	Effective *n* = 14
		Not effective *n* = 3
	Physical functioning (*n =* 19)	Effective *n* = 17
		Not effective n = 2
	Mental health and cognitive functioning (*n* = 18)	Effective *n* = 11
		Not effective *n* = 7
	Social functioning (*n* = 10)	Effective *n* = 8
		Not effective *n* = 2
	Generic health and well-being (*n* = 18)	Effective *n* = 11
		Not effective *n* = 7

^#^Definition of intervention functions: *Education*: ‘increasing knowledge or understanding’; *Enablement*: ‘increasing means/reducing barriers to increase capability (beyond education and training) or opportunity (beyond environmental restructuring)’; *Environmental restructuring*: ‘changing the physical or social context’; *Modeling*: ‘providing an example for people to aspire to or imitate’; *Persuasion*: ‘using communication to induce positive or negative feelings or stimulate action’; *Training*: ‘imparting skills’ [120].

^¥^ Definition of cultural adaption: *Linguistic strategies* improve the accessibility of programs/materials by providing them in the dominant or native language of the target population. *Sociocultural strategies* discuss health-related issues in the context of broader social and/or cultural values and characteristics of the target population [188].

**Table 2:** Intervention effectiveness by outcome cluster*.

**Anthropometrics outcomes**					
Targeted behaviour		Evidence of effectiveness (*n* = 12)	No evidence of effectiveness (*n* = 4)	All (*n* = 16)	Index of potential**
	**Physical activity**	**11**	**3**	**14**	**79%**
	**Healthy diet**	**5**		**5**	**100%**
Intervention functions^#^					
	**Education**	**4**	**3**	**7**	**57%**
	**Enablement**	**3**	**2**	**5**	**60%**
	**Environmental restructuring**	**3**	**1**	**4**	**75%**
	**Modelling**	**5**	**1**	**6**	**83%**
	Persuasion	2	3	5	40%
	**Training**	**6**		**6**	**100%**
Cultural adaption strategies^¥^		8	1	9	88%
	**Linguistic**	**6**		**6**	**100%**
	**Socio-cultural**	**3**	**1**	**4**	**75%**
BCT code	BCT label				
1.1	Goal-setting (behaviour)	3	3	6	50%
**1.2**	**Problem solving**	**4**	**3**	**7**	**57%**
1.8	Behavioural contract	2	2	4	50%
**2.3**	**Self-monitoring of behaviour**	**4**	**2**	**6**	**67%**
**3.1**	**Social support (unspecified)**	**8**	**3**	**11**	**73%**
**4.1**	**Instruction on how to perform the behaviour**	**9**	**2**	**11**	**82%**
**5.1**	**Information about health consequences**	**5**	**4**	**9**	**56%**
**6.1**	**Demonstration of the behaviour**	**6**	**1**	**7**	**86%**
**6.2**	**Social comparison**	**4**	**1**	**5**	**80%**
**8.1**	**Behavioural practice/rehearsal**	**6**	**1**	**7**	**86%**
**12.5**	**Adding objects to the environment**	**3**	**1**	**4**	**75%**
**Health behaviour outcomes**					
Targeted behaviour		Evidence of effectiveness (*n* = 15)	No evidence of effectiveness (*n* = 2)	All (*n* = 17)	Index of potential**
	**Physical activity**	**12**	**2**	**14**	**86%**
	**Healthy diet**	**5**	**2**	**7**	**71%**
Intervention functions^#^					
	**Education**	**7**	**1**	**8**	**88%**
	**Enablement**	**7**	**2**	**9**	**78%**
	**Modelling**	**4**		**4**	**100%**
	**Persuasion**	**8**	**2**	**10**	**80%**
	**Training**	**6**		**6**	**100%**
Cultural adaption strategies^¥^		8	1	9	89%
	**Linguistic**	**6**	**1**	**7**	**86%**
	**Socio-cultural**	**4**	**1**	**5**	**80%**
BCT code	BCT label				
**1.1**	**Goal-setting (behaviour)**	**10**	**1**	**11**	**91%**
**1.2**	**Problem solving**	**10**	**1**	**11**	**91%**
**1.8**	**Behavioural contract**	**3**	**1**	**4**	**75%**
**2.3**	**Self-monitoring of behaviour**	**6**		**6**	**100%**
**3.1**	**Social support (unspecified)**	**10**	**1**	**11**	**91%**
**4.1**	**Instruction on how to perform the behaviour**	**7**	**1**	**8**	**88%**
**5.1**	**Information about health consequences**	**8**	**3**	**11**	**73%**
**5.3**	**Information about social and environmental consequences**	**3**	**1**	**4**	**75%**
**6.1**	**Demonstration of the behaviour**	**4**		**4**	**100%**
**6.2**	**Social comparison**	**6**	**1**	**7**	**86%**
**8.1**	**Behavioural practice/rehearsal**	**6**		**6**	**100%**
9.2	Pros and cons	2	2	4	50%
**12.5**	**Adding objects to the environment**	**4**		**4**	**100%**
**Physical function outcomes**					
Targeted behaviour		Evidence of effectiveness (*n* = 17)	No evidence of effectiveness (*n* = 2)	All (*n* = 19)	Index of potential**
	**Physical activity**	**15**	**2**	**17**	**88%**
Intervention functions^#^					
	**Education**	**6**	**1**	**7**	**86%**
	**Enablement**	**8**	**1**	**9**	**89%**
	**Modelling**	**10**	**1**	**11**	**91%**
	**Persuasion**	**6**		**6**	**100%**
	**Training**	**6**	**1**	**7**	**86%**
Cultural adaption strategies^¥^		13	2	15	87%
	**Linguistic**	**11**	**2**	**13**	**85%**
	**Socio-cultural**	**6**	**1**	**7**	**86%**
BCT code	BCT label				
**1.1**	**Goal-setting (behaviour)**	**8**		**8**	**100%**
**1.2**	**Problem-solving**	**9**		**9**	**100%**
**2.3**	**Self-monitoring of behaviour**	**5**		**5**	**100%**
**3.1**	**Social support (unspecified)**	**8**	**1**	**9**	**89%**
**4.1**	**Instruction on how to perform the behaviour**	**14**	**1**	**15**	**93%**
**5.1**	**Information about health consequences**	**4**	**2**	**6**	**67%**
**6.1**	**Demonstration of the behaviour**	**13**	**1**	**14**	**93%**
**6.2**	**Social comparison**	**4**	**1**	**5**	**80%**
**8.1**	**Behavioural practice/rehearsal**	**14**	**1**	**14**	**93%**
**8.6**	**Generalisation of a target behaviour**	**5**	**1**	**6**	**83%**
**Mental health and cognitive function outcomes**					
Targeted behaviour		Evidence of effectiveness (*n* = 11)	No evidence of effectiveness (*n* = 7)	All (*n* = 18)	Index of potential**
	**Physical activity**	**10**	**5**	**15**	**67%**
Intervention functions^#^					
	**Education**	**5**	**3**	**8**	**63%**
	**Enablement**	**6**	**3**	**9**	**67%**
	**Modelling**	**4**	**2**	**6**	**67%**
	Persuasion	1	4	5	20%
	**Training**	**5**	**4**	**9**	**56%**
Cultural adaption strategies^¥^		10	3	13	77%
	**Linguistic**	**9**	**2**	**11**	**82%**
	**Socio-cultural**	**4**	**1**	**5**	**80%**
BCT code	BCT label				
1.1	Goal-setting (behaviour)	3	3	6	50%
**1.2**	**Problem solving**	**5**	**4**	**9**	**56%**
1.8	Behavioural contract	2	3	5	40%
2.3	Self-monitoring of behaviour	2	3	5	40%
**3.1**	**Social support (unspecified)**	**7**	**5**	**12**	**58%**
**4.1**	**Instruction on how to perform the behaviour**	**8**	**4**	**12**	**67%**
**5.1**	**Information about health consequences**	**4**	**2**	**6**	**67%**
**6.1**	**Demonstration of the behaviour**	**6**	**2**	**8**	**75%**
**6.2**	**Social comparison**	**4**	**2**	**6**	**67%**
**8.1**	**Behavioural practice/rehearsal**	**6**	**3**	**9**	**67%**
**8.6**	**Generalisation of a target behaviour**	**4**	**1**	**5**	**80%**
**12.5**	**Adding objects to the environment**	**3**	**2**	**75**	**60%**
**Social function outcomes**					
Targeted behaviour		Evidence of effectiveness (*n* = 8)	No evidence of effectiveness (*n* = 2)	All (*n* = 10)	Index of potential**
	**Physical activity**	**5**	**1**	**6**	**83%**
	**Social functioning**	**4**	**1**	**5**	**80%**
Intervention functions^#^					
	**Education**	**3**	**1**	**4**	**75%**
	**Modelling**	**4**		**4**	**100%**
	**Persuasion**	**4**	**1**	**5**	**80%**
	**Training**	**5**		**5**	**100%**
Cultural adaption strategy^¥^		5	2	7	71%%
	**Linguistic**	**3**	**1**	**4**	**75%**
BCT code	BCT label				
**1.1**	**Goal-setting (behaviour)**	**5**		**5**	**100%**
**1.2**	**Problem solving**	**4**		**4**	**100%**
**2.3**	**Self-monitoring of behaviour**	**4**		**4**	**100%**
**3.1**	**Social support (unspecified)**	**4**	**1**	**5**	**80%**
**4.1**	**Instruction on how to perform the behaviour**	**8**	**1**	**9**	**89%**
**6.1**	**Demonstration of the behaviour**	**6**	**1**	**7**	**86%**
**6.2**	**Social comparison**	**5**	**1**	**6**	**83%**
**8.1**	**Behavioural practice/rehearsal**	**7**	**1**	**8**	**88%**
**Generic health and well-being outcomes**					
Targeted behaviour		Evidence of effectiveness (*n* = 11)	No evidence of effectiveness (*n* = 7)	All (*n* = 18)	Index of potential**
	**Physical activity**	**10**	**5**	**15**	**67%**
Intervention functions^#^					
	**Education**	**6**	**4**	**10**	**60%**
	Enablement	4	5	9	44%
	**Modelling**	**6**	**1**	**7**	**86%**
	Persuasion	3	4	7	43%
	**Training**	**5**	**1**	**6**	**83%**
Cultural adaption strategy^¥^		7	5	12	58%
	**Linguistic**	**6**	**3**	**9**	**67%**
BCT code	BCT label				
1.1	Goal-setting (behaviour)	3	4	7	43%
1.2	Problem solving	4	4	8	50%
1.8	Behavioural contract	1	4	5	20%
2.2	Feedback on behaviour	1	3	4	25%
2.3	Self-monitoring of behaviour	1	3	4	25%
3.1	Social support (unspecified)	5	6	11	45%
**4.1**	**Instruction on how to perform the behaviour**	**10**	**2**	**12**	**83%**
5.1	Information about health consequences	3	3	6	50%
**6.1**	**Demonstration of the behaviour**	**7**	**2**	**9**	**78%**
**6.2**	**Social comparison**	**4**	**3**	**7**	**57%**
**8.1**	**Behavioural practice/rehearsal**	**7**	**2**	**9**	**78%**
**8.6**	**Generalisation of a target behaviour**	**6**	**2**	**8**	**75%**
12.5	Adding objects to the environment	1	3	4	25%
15.1	Verbal persuasion about capability	2	2	4	50%

BCT, behaviour change technique

*Only characteristics identified in at least four interventions within each cluster are reported for that cluster.

** Among all interventions featuring the focal characteristic, the percentage of interventions showing evidence of potential effectiveness for at least one variable in the relevant outcome cluster. Entries in bold are components found to show promise (index of potential > 50%).

^#^Definitions of intervention functions: education, ‘increasing knowledge or understanding’; enablement, ‘increasing means/reducing barriers to increase capability (beyond education and training) or opportunity (beyond environmental restructuring)’; environmental restructuring, ‘changing the physical or social context’; modelling, ‘providing an example for people to aspire to or imitate’; persuasion, ‘using communication to induce positive or negative feelings or stimulate action’; training, ‘imparting skills’ (ref. [120], p. 7).

^¥^ Definitions of cultural adaption strategies: linguistic, improving programme/materials accessibility by providing them in the dominant or native language of the target population; socio-cultural, discussing health-related issues in the context of broader social and/or cultural values and characteristics of the target population (ref. [188], p. 135–136.

Eight interventions were delivered individually [145, 149, 154, 159, 165, 171, 184, 185, 187], 18 were provided in groups [148, 152, 153, 156, 158, 163, 164, 169, 170, 175–180, 182–186, 194] and 13 used a combination of individual and group formats [144, 146, 147, 150, 151, 155, 157, 160–162, 166, 172–174, 181]. Different outcomes of the same interventions were reported separately for the Healthy Habits Program [174, 181], Well Elderly Lifestyle Redesign [146, 160], Experience Corps [147, 157], and Active Choices and Active Living Every Day [184, 185]. For the Healthy Habits Program, Hau et al. [181] build upon Lu et al’s [174] pilot study using a community-engaged approach. Several authors reported data from the same study. Clark *et al*. [146] and Juang *et al*. [160] reported data from the Lifestyle Redesign intervention, and Manson *et al*. [175, 176] and Taylor-Piliae *et al*. [178, 179] described two different tai chi interventions. This resulted in 39 articles reporting on 35 interventions. Manson *et al*. [176] and Wilcox *et al*. [185] reported data from previous studies, also included in this review [175, 184].

The number of functions per intervention ranged from one to four. Common functions were education, enablement and persuasion.

Twenty-four interventions implemented cultural adaptions, with four cultural adaption strategies identified. The number of such strategies per intervention ranged from one to three. Most of these studies took the native languages of the participants into account (linguistic strategy).

Of the 93 possible BCTs, 36 were identified in at least one intervention (S5 and S6 Tables). All BCT clusters except scheduled consequences and covert learning were represented. All interventions included multiple BCTs in various clusters (14 of 19), with all interventions encompassing two or more BCTs. The number of BCTs per intervention ranged from 2 to 15. The most frequently used BCTs were social support (unspecified) and instruction on how to perform the behaviour. Most studies targeted one behaviour, most frequently PA. Eleven studies targeted two behaviours (i.e. PA, healthy diet, social functioning). The authors of 14 studies specified that the interventions were based on behaviour change theories or models [144, 150–152, 156, 161–163, 165, 166, 173, 184–186]. Among all included interventions, 50 BCTs were coded as probably present (+) and 215 BCTs were coded as definitely present (++); S6 Table.

The frequency of contact with participants ranged from 1 to 34 sessions, with nine studies involving individually tailored programmes [144, 146, 151, 152, 154, 160, 162, 171, 187]. Intervention durations were 1 day; 6, 8, 9, 12, 16 and 20 weeks; 6 months; and 1 year. Thirty studies involved single follow-up evaluations, conducted 2 weeks to 2 years after baseline assessments [145, 146, 148–151, 153, 156, 157, 159, 160, 162–166, 169–171, 173, 175–182, 184, 186, 187, 194]. The remaining 13 studies had multiple (two or three) follow-up time points covering periods of 6 weeks to 4 years [144, 152, 154, 155, 157, 158, 161, 172, 174, 178, 179, 183, 185].

Among the 39 interventions assessing health and well-being outcomes, 12 of 16 showed effectiveness for anthropometrics outcomes, and 15 of 17 showed effectiveness for health behaviour outcomes. In addition, effectiveness was shown for 17 of 19 interventions assessing physical function outcomes, 11 of 18 examining mental health and cognitive function outcomes, 8 of 10 social function outcomes and 11 of 18 assessing generic health and well-being.

Assessment of the number of effective interventions among all interventions in each outcome cluster revealed that individually delivered interventions seemed to be less effective for all outcome clusters (one of four individually delivered interventions was effective for anthropometrics outcomes, one of four for health behaviour outcomes, two of three for physical function outcomes, one of three for mental health and cognitive function outcomes, one of four for generic health and well-being outcomes, and no such intervention was effective for social function outcomes). Interventions implemented in group-based sessions (alone or in combination with individual sessions) were most effective for al outcome clusters (9 of 11 group-based interventions were effective for anthropometrics outcomes, 10 of 12 for health behaviour outcomes, 15 of 17 for physical function outcomes, 8 of 13 for mental health and cognitive function outcomes, 8 of 10 for social function outcomes and 9 of 13 such interventions were effective for generic health and well-being outcomes).

### BCTs used in trials characterised as promising

BCTs targeting health and/or well-being, identified from descriptions of interventions in the included articles, are summarised by outcome cluster in Table 2. BCTs used in individual trials are shown in S5 and S6 Tables.

### Anthropometrics outcomes

A total of 12 interventions assessing anthropometrics outcomes resulted in significant improvements [144, 155, 156, 158, 161, 164, 174, 176–179, 184, 185], with most of these interventions targeting PA (Table 2). Five of six identified intervention functions were deemed promising: education (IP = 57%), enablement (IP = 67%), environmental restructuring (IP = 75%), modelling (IP = 83%) and training (IP = 100%). Eight effective interventions implemented cultural adaption strategies, most providing their programmes/materials in participants’ native languages (linguistic strategy; IP = 100%). Socio-cultural strategies (i.e. incorporation of traditional cuisine, culturally relevant music; IP = 75%) also showed promising effects. Nine BCTs were identified as promising: problem solving (IP = 57%), self-monitoring of behaviour (IP = 67%), social support (unspecified; IP = 73%), instruction on how to perform the behaviour (IP = 82%), information about health consequences (IP = 56%), demonstration of the behaviour (IP = 86%), social comparison (IP = 80%), behavioural practice/rehearsal (IP = 86%) and addition of objects to the environment (IP = 75%).

### Health behaviour outcomes

Effectiveness was shown for 14 of 17 interventions assessing behaviour outcomes [144, 145, 147–149, 157–159, 162, 163, 165, 172, 173, 180–182, 184, 185, 194], with most of these interventions targeting PA. Five intervention functions were identified as promising, ranging from one to four per intervention. Eight effective interventions included cultural adaption (linguistic and socio-cultural) strategies. In total, 13 BCTs were identified as promising: goal-setting (behaviour; IP = 91%), problem solving (IP = 91%), behavioural contract (IP = 75%), self-monitoring of behaviour (IP = 100%), social support (unspecified; IP = 91%), instruction on how to perform the behaviour (IP = 88%), information about health consequences (IP = 73%), information about social and environmental consequences (IP = 75%), demonstration of the behaviour (IP = 100%), social comparison (IP = 86%), behavioural practice/rehearsal (IP = 100%), and addition of objects to the environment (IP = 100%).

### Physical function outcomes

Nineteen BCIs employed physical function outcome measures [146, 147, 150, 153, 159, 160, 163, 164, 166, 170, 171, 173–181, 183, 187]; two interventions resulted in no significant improvement [153, 174]. All interventions targeted PA, except for one which targeted social functioning [147]. All six intervention functions were identified as promising. Fourteen interventions assessing physical functioning involved cultural adaption strategies, most of which were linguistic and socio-cultural. Goal-setting (behaviour; IP = 100%), problem solving (IP = 100%), self-monitoring of behaviour (IP = 100%), social support (unspecified; IP = 89%), instruction on how to perform the behaviour (IP = 93%), information about health consequences (IP = 60%), demonstration of the behaviour (IP = 93%), social comparison (IP = 80%), behavioural practice/rehearsal (IP = 93%), generalisation of a target behaviour (IP = 83%), and addition of objects to the environment (IP = 80%) were identified as promising BCTs.

### Mental health and cognitive function outcomes

Eleven interventions resulted in significant progress in mental health and cognitive function outcome measures [146, 153, 160, 161, 163, 170, 171, 174, 178, 179, 181, 183, 184], with most of these interventions targeting PA. Four intervention functions were deemed promising: education (IP = 63%), enablement (IP = 67%), modelling (IP = 67%), and training (IP = 56%). Eleven effective interventions involved cultural adaption strategies, most of which were linguistic and socio-cultural. Problem solving (IP = 56%), social support (unspecified; IP = 58%), instruction on how to perform the behaviour (IP = 67%), information about health consequences (IP = 67%), demonstration of the behaviour (IP = 75%), social comparison (IP = 67%), behavioural practice/rehearsal (IP = 67%), generalisation of a target behaviour (IP = 80%) and addition of objects to the environment (IP = 60%) were identified as promising BCTs.

### Social function outcomes

Ten interventions employed social function outcome measures, with eight interventions resulting in significant improvements in social contact, social activities, social support, and loneliness [146, 147, 150, 151, 160, 166, 169, 170, 173, 178, 179, 194]. Most social-function interventions targeted PA. Four promising intervention functions were identified: education (IP = 75%), modelling (IP = 100%), persuasion (IP = 80%) and training (IP = 100%). Five effective interventions included linguistic strategies. Eight BCTs were identified as promising: goal-setting (behaviour; IP = 100%), problem solving (IP = 100%), self-monitoring of behaviour (IP = 100%), social support (unspecified; IP = 80%), instruction on how to perform the behaviour (IP = 89%), demonstration of the behaviour (IP = 86%), social comparison (IP = 83%) and behavioural practice/rehearsal (IP = 78%).

### Generic health and well-being outcomes

Eighteen interventions employed generic health and well-being outcome measures, covering aspects such as vitality, life satisfaction, stress, pain and fatigue [146, 151, 153, 154, 159, 160, 163, 164, 169–172, 176–180, 183–186], ten interventions showed to be significant [146, 160, 164, 169, 170, 176–180, 183, 186]. Eleven interventions targeted PA, with education (IP = 60%), modelling (IP = 86%) and training (IP = 83%) identified as promising intervention functions. Linguistic strategies were implemented in most of the effective interventions involving cultural adaption. In total, five BCTs were deemed promising: instruction on how to perform the behaviour (IP = 83%), demonstration of the behaviour (IP = 78%), social comparison (IP = 57%), behavioural practice/rehearsal (IP = 78%) and generalisation of a target behaviour (IP = 79%).

### BCTs used in trials involving assessment ≥3 months after baseline characterised as promising

The impacts of interventions involving assessment ≥3 months after baseline on effectiveness are summarised in S7 Table. Of the 27 interventions in this category [144–147, 150, 155, 157–161, 163–166, 169–172, 174–179, 181–187], 10 were effective assessing anthropometrics outcomes [144, 155, 156, 158, 161, 164, 174, 176, 178, 179, 184, 185]. Nine interventions were effective assessing behaviour outcomes [144, 147, 149, 157–159, 163, 165, 172, 182], 16 for physical functioning [146, 147, 150, 159, 160, 163, 164, 166, 170, 171, 175–179, 181, 183, 187], 10 for mental health and cognitive functioning [146, 161, 163, 170, 171, 174, 178, 179, 181, 183, 184], 6 for social functioning [146, 147, 150, 166, 169, 178, 179] and 9 effective interventions employed generic health and well-being outcomes [146, 164, 169, 170, 176, 178, 179, 183–186]. Only one BCT, instruction on how to perform the behaviour, remained promising in all outcome clusters. Problem solving (IP = 60%), self-monitoring of behaviour (IP = 75%), social support (unspecified; IP = 75%), instruction on how to perform the behaviour (IP = 89%), information about health consequences (IP = 63%) and behavioural practice/rehearsal (IP = 100%) remained promising among the interventions assessing anthropometrics outcomes. Interventions assessing behaviour outcomes employed six promising BCTs: goal-setting (behaviour; IP = 100%), problem solving (IP = 100%), self-monitoring of behaviour (IP = 100%), social support (unspecified; IP = 8%), instruction on how to perform the behaviour (IP = 100%) and information about health consequences (IP = 88%). In interventions assessing physical outcomes and mental health and cognitive function outcomes, all BCTs except social comparison and generalisation of a target behaviour were promising. Only three BCTs were promising in interventions assessing social functioning: instruction on how to perform the behaviour (IP = 86%), demonstration of the behaviour (IP = 80%) and behavioural practice/rehearsal (IP = 83%). All BCTs in the generic health and well-being outcome cluster were promising.

## Discussion

The aim of this review was to summarise the current body of literature on BCTs that are present in effective behavioural change interventions which target the health and well-being of older migrants. Thirty-nine BCIs showed mixed effects in health and well-being outcome clusters, and 13 BCTs were identified as promising for at least one outcome cluster. Four BCTs and two intervention functions were identified as promising for all outcome clusters; only instruction on how to perform the behaviour and training remained promising for all outcome clusters ≥3 months after baseline. Twenty-four studies included cultural adaption, most commonly using linguistic and socio-cultural strategies; linguistic strategies were identified as promising for all outcome clusters (except social functioning at ≥3 months after baseline).

Given the lack of previous systematic reviews focusing on older migrants, the results from this study can be compared with systematic reviews of BCTs used to promote health and/or well-being in other populations. Promising BCTs included: goal-setting (behaviour), problem-solving, self-monitoring of behaviour, social support (unspecified), instruction on how to perform the behaviour, demonstration how to perform the behaviour, behavioural practice/rehearsal, information about health consequences, information about social and environmental consequences, behavioural contract, social comparison, generalisation of a target behaviour and adding objects to the environment.

The finding that goal-setting (behaviour), problem solving and self-monitoring of behaviour were promising is in line with results from previous studies [116, 117, 122, 195–197]. The definition of goals, for instance by developing PA plans or by monitoring behaviour using a pedometer or log book, has been associated with better intervention effects in previous research [116, 122, 195, 197]. In contrast, the systematic review conducted by French *et al*. [198] showed a negative association of goal-setting (behaviour) with PA among older individuals. Further research is needed to explore whether this difference in study findings is due to differences in the populations examined or other factors.

Social support (unspecified) has been shown to be effective in promoting healthy behaviours, such as PA [195, 199] and good dietary habits [117, 200, 201]. Older individuals have also reported that social support facilitates the participation in PA [202]. Social support from family members and friends may be more important for the initiation of PA among community-dwelling older individuals [203–205], whereas social support from sport professionals, health care providers and exercise group members may be more important for the maintenance of PA [206].

Instruction on how to perform the behaviour has been shown in previous research to be associated with better outcomes of interventions targeting healthy behaviours, such as smoking cessation and PA [116, 118, 207, 208]. Interventions involving such instruction provided, for example, handouts or exercise training, and some also involved the information about health, social and/or environmental consequences, which have been identified as promising for the promotion of smoking cessation [207, 209].

To our knowledge, our review showed for the first time that the BCTs of behavioural practice/rehearsal, behavioural contract, social comparison, generalisation of a target behaviour, and addition of objects to the environment can be promising for the promotion of health behaviours. Further research is needed to explore the effectiveness of these BCTs in achieving this goal.

In line with previous research, the results of this systematic review suggest that group-based interventions are more effective than individually delivered interventions [210, 211].

Previous research has indicated that people from disadvantaged backgrounds are less successful in achieving behaviour changes (e.g. cessation of smoking) following participation in formal programmes than less disadvantaged socio-economic groups [212]. However, Michie and colleagues [213] indicated that BCIs can be effective among individuals with such backgrounds. They proposed that a small set of BCTs may be more effective for this part of the population than would interventions combining large numbers of different BCTs. In contrast to other literature showing that BCT combinations are more likely to show effectivity in promoting healthy behaviour, suggesting that greater numbers of BCTs are associated with increased intervention effectiveness [197, 214–216]. The BCTs that we identified as promising may work differently for older migrants than for native older individuals. We cannot draw specific conclusions about comparative intervention effectiveness in these two populations because most interventions included in this review targeted older migrants.

The number of behaviours targeted might also impact intervention effectiveness; in this systematic review, 11 of 35 interventions targeted two behaviours. Efforts to change multiple behaviours simultaneously, rather than changing behaviours individually, have been found to be more effective in changing at least one behaviour [217]. Precisely how this process works is unclear, but it has been proposed that a successful change in one behaviour can enable change in other behaviours, and that the targeting of behavioural patterns may be more suitable [218]. For example, Schölmerich and Kawachi [219, 220] suggest that multi-level interventions (which target change at levels including policy, community, organisational, interpersonal and intrapersonal) exert the strongest effects on health outcomes. The implementation of health behaviour interventions is a complex process; more insight is needed on the optimal number of BCTs to implement and behaviours to target for the promotion of health behaviour among older migrants.

Four-teen research groups reported the use of eight behaviour change theories and models to underpin interventions in the articles included in this review; research has shown that theory-driven interventions are more effective [216, 221–224]. Although, the reasons for the incorporation of some BCTs in interventions were not always clear, our findings show that a variety of BCTs can be implemented to improve the health and/or well-being of older migrants. These findings reflect the heterogeneity of BCTs and technical intervention formats that can effectively promote health and/or well-being among older migrants.

This review showed mixed results for intervention effectiveness among outcome clusters. First, several interventions had no impact on anthropometric [145, 154, 159, 173], health behaviour [145, 148, 181], physical function [153, 174], mental health and cognitive function [147, 151, 152, 159, 180, 184, 187], social function [151, 170] and/or generic health and well-being [151, 153, 159, 163, 171, 172, 184, 185] outcomes, highlighting the possibility that BCIs will fail and emphasising the importance of identifying intervention components that may contribute to effectiveness [225]. However, the identification of effective BCTs is difficult, given the pool of different combinations of BCTs within and across studies. Second, more than half of the interventions were effective for each outcome cluster in this review, in contrast to a previous study in which less than half of interventions effectively enhanced health and well-being outcomes [121]. An explanation for this difference might be publication bias among included articles, or our definition of a promising BCT based on positive effects in at least one outcome in a cluster, which may have led to the overestimation of effectiveness. For instance, two interventions with promising BCTs each changed only one of four health behaviours [147, 157, 159] or social function outcomes [147, 157]. Reasons for the mixed results may be the lack of clarity about key intervention components [226], lack of a theoretical framework [227] and/or use of inappropriate BCTs for the target population [228].

Most of the included interventions targeted PA, with three interventions addressing social functioning. Social functioning is an important social determinant of health (i.e. social participation, loneliness) and has impacts on health outcomes (i.e. health-related quality of life, cognitive impairment, dementia, depression and mortality) [108–111]. Previous research has indicated that interventions addressing social participation, social isolation and loneliness in community-dwelling ethnic minority groups can be effective [229]. Our review highlights the importance of investing in the development and implementation of interventions addressing social functioning among older migrants, as we found only a small number of studies on this subject.

### Implications for policy and intervention design

This study provides an overview of promising BCTs for six health/well-being outcome clusters, which can be used for intervention development. For example, three BCTs (demonstration of the behaviour, social comparison, and behavioural practice/rehearsal) were promising for all outcome clusters, suggesting that they could be used in the development of interventions targeting older migrants’ health and well-being. Behavioural contract establishment and the provision of information about social and environmental consequences were promising only for health behaviour outcomes. Thus, the implementation of these BCTs in BCIs targeting such outcomes (e.g., PA) would likely be beneficial. In addition, six intervention functions were identified as promising, for all outcome clusters in two cases (education and modeling). Thus, BCIs that increase participants’ knowledge and understanding (education) and provide examples for people to aspire to or imitate (modeling) are likely to improve health and well-being among older migrants.

The influence of culture is important to consider in intervention development, as many cultural factors influence people’s health-related beliefs [230] and, in turn, might influence their actions and impact intervention effectiveness. For example, previous findings suggest that culture impacts the role of social support with regard to PA [231–233]. Interventions included in this review that involved cultural adaption employed single cultural adaptive strategies Linguistic and sociocultural strategies to make interventions culturally appropriate were found to be promising in this study. This finding suggests that information about an intervention and the materials used therein should be provided in the native language of the target population. In addition, the social and/or cultural values of the target population should be considered when BCIs are developed.

Most studies included in this review were conducted in high-income countries, which resulted in the exclusion of certain countries and migrant groups. This situation has to do partly with funding for migrant research. Funding agencies could aid research efforts by investing in studies conducted with migrant (sub)groups in various countries.

### Strengths and limitations

The results of our review must be viewed with caution because of several limitations. Our findings have limited generalisability to the international population of older migrants, as all trials were conducted in high-income countries, primarily the United States and only one study in Europe. This lack of diversity in studies shows that academic research on ageing among older migrants remains scarce. The search of this study yielded several study protocols of future interventions targeting health and/or well-being among older migrants in various countries, this shows that there will be more interventions for this population in the future.

Despite the heterogeneity that exists among and within subgroups of migrant populations (e.g. in terms of country of origin, reason for migration, age at migration, number of years living in the host country), our review focused on migrants in general and included studies conducted with diverse racial/ethnic groups. Thus, we could not draw conclusions about the effectiveness of BCTs for specific migrant groups. Our findings shed light on ageing among migrants, but as older migrants form a heterogeneous group, suggestions about behavioural change interventions need to be aligned with country and population subgroup contexts. In addition, this study did not report possible costs associated with older migrant health.

The BCTTv1 facilitates the accurate identification of intervention content and provides a useful overview of BCTs and their definitions. However, it does not permit consideration that BCTs might be effective only under the specific conditions in which the interventions are delivered (i.e. the potential for interaction between BCTs and intervention contexts) [234]. In addition, overlap may exist in BCTs delivered in the intervention and control groups [235, 236], which hampers the drawing of conclusions about which BCTs do and do not work. In this study, this factor was taken into account by including only BCTs delivered in the intervention group, and not in the control group, in the analysis, as recommended by Peters and colleagues [191]. Moreover, the implementation of sets of BCTs in interventions might pose a challenge because some BCTs may be employed simultaneously. For example, a previous study showed that interventions combining self-monitoring, goal-setting and action planning were twice as effective as those that did not [122]. These contextual factors might impact the effect size and interact with the intervention content, thereby confounding the BCT–effect size relationship [191, 237]. Despite these limitations, however, the BCTTv1 is a useful tool for the assessment of effectiveness at the BCT level, as demonstrated by analyses conducted in various health behaviour contexts [116, 118, 122]. For example, one study showed that problem-solving, social support, goal-setting, the use of prompts and the provision of feedback on behaviour were associated with greater intervention effects on fruit and vegetable consumption compared with interventions not including these BCTs [117]. To fully understand the effects of BCTs on behaviour, the classification of knowledge about other aspects of BCIs and its inclusion in the analysis of BCT effectiveness are crucial. Michie and colleagues [189] noted the need to develop proper methods to link evidence from various types of evaluation, to permit the drawing of conclusions regarding effect sizes of BCT combinations tailored to the targeted behaviours and contexts.

The BCTTv1 is a valuable tool, as it assisted to relate descriptions of intervention content to definitions of BCTs. Vigorous BCT coding depends on the provision of complete descriptions of interventions in the original studies [189, 238]. In addition, more BCTs than reported may have been implemented in the interventions in practice. We did not contact the authors of the included studies; rather, we addressed this situation by coding BCTs as probably present (+) in addition to definitely present (++) following the coding scheme [192]. BCTs were coded using only published papers and, when available, published protocols, which might have increased the risk of publication bias. Additionally, some BCTs may not have been captured, as interventions are often poorly described [239]. Detailed intervention description is important not only for BCT coding, but also intervention delivery. BCT delivery can be challenging; for example, goal setting requires specific behavioural, measurable, observable and challenging, yet realistic, goals [240–242]. Published descriptions of interventions typically provide insufficient details to check for appropriate delivery of this BCT and others.

Our study included articles described health and well-being outcomes in different ways, and not all articles mentioned effect sizes, thereby limiting our ability to conduct a meta-analysis. Although our review revealed differences in terms of health and well-being outcome measures, features beyond ethnic association alone may have contributed to this variance. For instance, contextual factors such as limited proficiency in the native language, social isolation and barriers to health and well-being behaviours might be stronger predictors among migrant (sub)groups. Migrants are extremely diverse in terms of migration patterns, nativity, language and socio-economic status. Thus, interventions that consider how these factors may affect health and well-being behaviours by subgroup are critical. Although linguistic tailoring increases accessibility and acceptability, more adaptions might lower barriers to the performance of healthy behaviour(s).

### Future research

In this study we focused on the general older migrant population. Future research could focus on the effectiveness of the BCTs examined in various contexts and subgroups of older migrants. For example, acculturation is related to health behaviours [243–245], but may differ among migrant groups and host countries. Moreover, all interventions examined in this review incorporated combinations of BCTs, which may increase effectiveness [122]. The specific BCT combinations that most effectively promote health and/or well-being among older migrants, however, have not been identified clearly. Future research could focus on examining BCT combinations and the interplay among them, as well as the mechanisms by which effective BCTs modify behaviour, to enable the development of interventions with components that are more likely to be effective [246] and to better explain interventions’ effects [247]. In addition, the majority of participants in the studies included in the present review were female (S8 Table). An in-depth understanding of this bias will be important for future research to meet the needs of other gender groups. Finally, the quality of the included studies was judged using various categories and the risk of bias. In future research, several other sources of bias may also need to be considered. For example, detailed descriptions of the interventions (e.g., language use in intervention materials), which may not always be provided in study publications, should be considered as such factors may have impacts on response rates.

## Conclusions

We identified 13 promising BCTs used in interventions to promote health and well-being among older migrants. Older migrants are heterogeneous, with diverse subgroups within and across countries, and have different unhealthy behaviours to address. In addition, future research should examine the effectiveness of these BCTs in various contexts and among different subgroups of older migrants, as well as the mechanisms through which these BCTs act. In addition, given the paucity of interventions in which cultural adaption has been taken into account in alignment with the target group, future BCIs should consider cultural appropriateness for different older migrant (sub)groups. Our findings may guide future research with the goal of developing culturally appropriate interventions incorporating promising BCTs that can change behaviours and improve the health and well-being of older migrants.

## Supporting information

S1 Table PRISMA 2009 checklist. (DOCX)

S2 Table Search strategy. (DOCX)

S3 Table Assessment of quality for included randomized controlled trials studies based on Cochrane tool. (DOCX)

S4 Table Assessment of quality for included non-controlled intervention studies based on Newcastle-Ottawa Scale. (DOCX)

S5 Table Study characteristics of included trials. (DOCX)

S6 Table Coded BCT per individual intervention. (DOCX)

S7 Table Intervention effectiveness involving assessment ≥ 3 months post baseline by outcome clusters. (DOCX)

S8 Table Summary of study characteristics. (DOCX)

## Abbreviations

BCI: Behaviour Change Intervention
BCT: Behaviour Change Technique
BCTTv1: Behaviour Change Technique Taxonomy version 1
IF: Intervention Function
IP: Index of Potential
NOS: Newcastle-Ottawa Scale
PA: Physical Activity
PRISMA: Preferred Reporting Items for Systematic reviews and Meta-Analyses
