# Supplementary material for: Behaviour change interventions to promote health and well-being among older migrants: A systematic review
Source: PLoS One. 2022 Jun 16;17(6):e0269778. doi: 10.1371/journal.pone.0269778 (PMC9202883; doi:10.1371/journal.pone.0269778)
Supplement: S2 Table — (DOCX) [file pone.0269778.s002.docx]

## **S2 Table. Search strategy**

| **embase.com 1711**  ('behavior change'/exp OR 'lifestyle'/de OR 'lifestyle modification'/exp OR diet/exp OR 'physical activity'/exp OR 'smoking **and** smoking related phenomena'/exp OR 'social behavior'/de OR 'social interaction'/de OR 'social participation'/de OR 'health behavior'/de OR 'feeding behavior'/de OR ((behav* NEAR/3 change*) OR lifestyle OR life-style OR diet OR (dietar* NEAR/3 pattern*) OR (physical* NEAR/3 (activ* OR inactiv*)) OR smoking OR (social* NEAR/3 (behav* OR activ* OR interact* OR participat* OR engage*)) OR (communit* NEAR/3 (engage* OR activit*)) OR smoke OR tobacco OR ((health* OR unhealth*) NEAR/3 (behav*)) OR ((health* OR unhealth* OR feeding OR eating) NEAR/3 behav*)):ab,ti) **AND** ('health promotion'/de OR (promot* OR interven* OR education* OR ((health*) NEAR/3 (literat*)) ):ab,ti) **AND** ('neighborhood'/exp OR 'community'/de OR 'urban population'/exp OR 'rural population'/exp OR 'community dwelling person'/de OR (neighborhood* OR neighbourhood* OR communit* OR urban OR rural):ab,ti) **AND** ('immigrant'/exp OR 'minority group'/exp OR (immigrant* OR morocc* OR turk* OR Minorit* OR ethnic* OR multiethnic*):ab,ti) **AND** ('aged'/exp OR 'middle aged'/exp OR 'home for the aged'/exp OR 'nursing home'/de OR 'nursing home patient'/de OR 'aging'/de OR 'geriatrics'/exp OR 'gerontology'/de OR 'geriatric nursing'/de OR 'gerontological research'/de OR 'gerontologist'/de OR 'geriatric care'/exp OR 'geriatric patient'/exp OR 'elderly care'/exp OR 'senescence'/exp OR 'geriatric disorder'/de OR 'geriatric surgery'/de OR 'geriatric assessment'/de OR 'frailty'/de OR 'Longevity'/de OR 'life extension'/de OR 'life expectancy'/de OR (elder* OR ((for-the-aged OR older) NEAR/6 (care OR people OR subject* OR person* OR patient* OR home OR homes OR housing OR adult* OR women OR woman OR female* OR men OR man OR male*)) OR very-old* OR frail* OR old*-age* OR oldest-old* OR ((aged) NEXT/1 (people OR subject* OR person* OR patient* OR population*)) OR senior* OR nursing-home* OR frail* OR aging OR ageing OR geriatric* OR Gerontolog* OR septagenarian* OR octagenarian* OR nonagenarian* OR centenarian* OR supercentenarian* OR senescen* OR Immunosenescen* OR Longevit* OR (life NEXT/1 (exten* OR expectan*)) OR gerontopsych* OR psychogeriat* OR geropsych* OR age-relat* OR ((55 OR 56 OR 57 OR 58 OR 59 OR 60 OR 61 OR 62 OR 63 OR 64 OR 65 OR 66 OR 67 OR 68 OR 69 OR 70 OR 71 OR 72 OR 73 OR 74 OR 75 OR 76 OR 77 OR 78 OR 79 OR 80 OR 81 OR 82 OR 83 OR 84 OR 85 OR 86 OR 87 OR 88 OR 89 OR 90 OR 91 OR 92 OR 93 OR 94 OR 95 OR 96 OR 97 OR 98 OR 99 OR 100 OR 101 OR 102 OR 103 OR 104 OR 105 OR 106 OR 107 OR 108 OR 109 OR 110 OR 111 OR 112 OR 113 OR 114 OR 115 OR 116 OR 117 OR 118 OR 119 OR 120 OR 121 OR 122) NEXT/1 (year* OR yr OR yrs OR and-older)) OR ((older-than OR age-of OR aged) NEXT/1 (55 OR 56 OR 57 OR 58 OR 59 OR 60 OR 61 OR 62 OR 63 OR 64 OR 65 OR 66 OR 67 OR 68 OR 69 OR 70 OR 71 OR 72 OR 73 OR 74 OR 75 OR 76 OR 77 OR 78 OR 79 OR 80 OR 81 OR 82 OR 83 OR 84 OR 85 OR 86 OR 87 OR 88 OR 89 OR 90 OR 91 OR 92 OR 93 OR 94 OR 95 OR 96 OR 97 OR 98 OR 99 OR 100 OR 101 OR 102 OR 103 OR 104 OR 105 OR 106 OR 107 OR 108 OR 109 OR 110 OR 111 OR 112 OR 113 OR 114 OR 115 OR 116 OR 117 OR 118 OR 119 OR 120 OR 121 OR 122))):kw,ab,ti) NOT ([Conference Abstract]/lim) AND [english]/lim | **Medline ovid 1642**  (exp Life Style/ OR exp diet/ OR exp Exercise/ OR exp smoking/ OR Health Behavior/ OR Smoking Cessation/ OR Smoking Reduction/ OR "Tobacco Use Cessation"/ OR exp Social Behavior/ OR exp Social Participation/ OR ((behav* ADJ3 change*) OR lifestyle OR life-style OR diet OR (dietar* ADJ3 pattern*) OR (physical* ADJ3 (activ* OR inactiv*)) OR smoking OR (social* ADJ3 (behav* OR activ* OR interact* OR participat* OR engage*)) OR (communit* ADJ3 (engage* OR activit*)) OR smoke OR tobacco OR ((health* OR unhealth*) ADJ3 (behav*)) OR ((health* OR unhealth* OR feeding OR eating) ADJ3 behav*)).ab,ti.) **AND** (health promotion/ OR (promot* OR interven* OR education* OR ((health*) ADJ3 (literat*)) ).ab,ti.) **AND** (Community Participation/ OR Community Health Services/ OR Community Networks/ OR Community Health Centers/ OR urban population/ OR rural population/ OR (neighborhood* OR neighbourhood* OR communit* OR urban OR rural).ab,ti.) **AND** ("Emigrants **and** Immigrants"/ OR minority groups/ OR (immigrant* OR morocc* OR turk* OR Minorit* OR ethnic* OR multiethnic*).ab,ti.) **AND** (exp Aged/ OR Health Services for the Aged/ OR Homes for the Aged/ OR Housing for the Elderly/ OR Nursing Homes/ OR exp Aging/ OR Geriatrics/ OR Geriatricians/ OR Geriatric Nursing/ OR Geriatric Assessment/ OR Geriatric Psychiatry/ OR Geriatric Dentistry/ OR Dental Care for Aged/ OR Life Expectancy/ OR (elder* OR ((for-the-aged OR older) ADJ6 (care OR people OR subject* OR person* OR patient* OR home OR homes OR housing OR adult* OR women OR woman OR female* OR men OR man OR male*)) OR very-old* OR frail* OR old*-age* OR oldest-old* OR ((aged) ADJ (people OR subject* OR person* OR patient*)) OR senior* OR nursing-home* OR frail* OR aging OR ageing OR geriatric* OR Gerontolog* OR septagenarian* OR octagenarian* OR nonagenarian* OR centenarian* OR supercentenarian* OR senescen* OR Immunosenescen* OR Longevit* OR (life adj (exten* OR expectan*)) OR gerontopsych* OR psychogeriat* OR geropsych* OR age-relat* OR (("65" OR "66" OR "67" OR "68" OR "69" OR "70" OR "71" OR "72" OR "73" OR "74" OR "75" OR "76" OR "77" OR "78" OR "79" OR "80" OR "81" OR "82" OR "83" OR "84" OR "85" OR "86" OR "87" OR "88" OR "89" OR "90" OR "91" OR "92" OR "93" OR "94" OR "95" OR "96" OR "97" OR "98" OR "99" OR "100" OR "101" OR "102" OR "103" OR "104" OR "105" OR "106" OR "107" OR "108" OR "109" OR "110" OR "111" OR "112" OR "113" OR "114" OR "115" OR "116" OR "117" OR "118" OR "119" OR "120" OR "121" OR "122") ADJ (year* OR yr OR yrs OR and-older)) OR ((older-than OR age-of OR aged) ADJ ("65" OR "66" OR "67" OR "68" OR "69" OR "70" OR "71" OR "72" OR "73" OR "74" OR "75" OR "76" OR "77" OR "78" OR "79" OR "80" OR "81" OR "82" OR "83" OR "84" OR "85" OR "86" OR "87" OR "88" OR "89" OR "90" OR "91" OR "92" OR "93" OR "94" OR "95" OR "96" OR "97" OR "98" OR "99" OR "100" OR "101" OR "102" OR "103" OR "104" OR "105" OR "106" OR "107" OR "108" OR "109" OR "110" OR "111" OR "112" OR "113" OR "114" OR "115" OR "116" OR "117" OR "118" OR "119" OR "120" OR "121" OR "122"))).kw,ab,ti.) AND english.la. | **Web of science 764**  TS=((((behav* NEAR/2 change*) OR "lifestyle" OR "life-style" OR "diet" OR (dietar* NEAR/2 pattern*) OR (physical* NEAR/2 (activ* OR inactiv*)) OR "smoking" OR (social* NEAR/2 (behav* OR activ* OR interact* OR participat* OR engage*)) OR (communit* NEAR/2 (engage* OR activit*)) OR "smoke" OR "tobacco" OR ((health* OR unhealth*) NEAR/2 (behav*)) OR ((health* OR unhealth* OR "feeding" OR "eating") NEAR/2 behav*))) **AND** ((promot* OR interven* OR education* OR ((health*) NEAR/2 (literat*)) )) **AND** ((neighborhood* OR neighbourhood* OR communit* OR "urban" OR "rural")) **AND** ((immigrant* OR morocc* OR turk* OR Minorit* OR ethnic* OR multiethnic*)) **AND** ((elder* OR (("for-the-aged" OR "older") NEAR/5 ("care" OR "people" OR subject* OR person* OR patient* OR "home" OR "homes" OR "housing" OR adult* OR "women" OR "woman" OR female* OR "men" OR "man" OR male*)) OR very-old* OR frail* OR old*-age* OR oldest-old* OR (("aged") NEAR/1 ("people" OR subject* OR person* OR patient* OR population*)) OR senior* OR nursing-home* OR frail* OR "aging" OR "ageing" OR geriatric* OR Gerontolog* OR septagenarian* OR octagenarian* OR nonagenarian* OR centenarian* OR supercentenarian* OR senescen* OR Immunosenescen* OR Longevit* OR ("life" NEAR/1 (exten* OR expectan*)) OR gerontopsych* OR psychogeriat* OR geropsych* OR age-relat*))) AND DT=(article) AND LA=(English) | **Cochrane CENTRAL 83**  (((behav* NEAR/3 change*) OR lifestyle OR life-style OR diet OR (dietar* NEAR/3 pattern*) OR (physical* NEAR/3 (activ* OR inactiv*)) OR smoking OR (social* NEAR/3 (behav* OR activ* OR interact* OR participat* OR engage*)) OR (communit* NEAR/3 (engage* OR activit*)) OR smoke OR tobacco OR ((health* OR unhealth*) NEAR/3 (behav*)) OR ((health* OR unhealth* OR feeding OR eating) NEAR/3 behav*)):ab,ti) **AND** ((promot* OR interven* OR education* OR ((health*) NEAR/3 (literat*))):ab,ti) **AND** ((neighborhood* OR neighbourhood* OR communit* OR urban OR rural):ab,ti) **AND** ((immigrant* OR morocc* OR turk* OR Minorit* OR ethnic* OR multiethnic*):ab,ti) **AND** ((elder* OR ((for-the-aged OR older) NEAR/6 (care OR people OR subject* OR person* OR patient* OR home OR homes OR housing OR adult* OR women OR woman OR female* OR men OR man OR male*)) OR very-old* OR frail* OR (old* next age*) OR (oldest next old*) OR ((aged) NEXT/1 (people OR subject* OR person* OR patient* OR population*)) OR senior* OR (nursing next home*) OR frail* OR aging OR ageing OR geriatric* OR Gerontolog* OR septagenarian* OR octagenarian* OR nonagenarian* OR centenarian* OR supercentenarian* OR senescen* OR Immunosenescen* OR Longevit* OR (life NEXT/1 (exten* OR expectan*)) OR gerontopsych* OR psychogeriat* OR geropsych* OR (age next relat*)):ab,ti) |
| --- | --- | --- | --- |
