# Supplementary material for: Behaviour change interventions to promote health and well-being among older migrants: A systematic review
Source: PLoS One. 2022 Jun 16;17(6):e0269778. doi: 10.1371/journal.pone.0269778 (PMC9202883; doi:10.1371/journal.pone.0269778)
Supplement: S3 Table — (DOCX) [file pone.0269778.s003.docx]

## **S3 Table: Assessment of quality for included randomized controlled trials studies based on Cochrane tool**

|  | Agurs-Collins, T.D. (1997) [[1](#_ENREF_1)] | Batik, O. (2008) [[2](#_ENREF_2)] | Clark, F.(2012) [[3](#_ENREF_3)] | Fernandez, S. (2008) [[4](#_ENREF_4)] | Fried, L.P. (2004) [[5](#_ENREF_5)] | Geller, K.S. (2012) [[6](#_ENREF_6)] | Holland, S.K. (2005) [[7](#_ENREF_7)] | Juang, C. (2018) [[8](#_ENREF_8)] | Keller, C. (2008) [[9](#_ENREF_9)] | Kim, B.H. (2013) [[10](#_ENREF_10)] | Kim, K.B. (2014) [[11](#_ENREF_11)] | Palta, P. (2012) [[12](#_ENREF_12)] | Parisi, J.M. (2015) [[13](#_ENREF_13)] | Qi, B.B. (2001) [[14](#_ENREF_14)] | Rejeski, W.J. (2014) [[15](#_ENREF_15)] | Resnick, B. (2008) [[16](#_ENREF_16)] | Sun, W.Y. (1996) [[17](#_ENREF_17)] | Wolf, R.L. (2009) [[18](#_ENREF_18)] | Yeom, H. (2013) [[19](#_ENREF_19)] |
| --- | --- | --- | --- | --- | --- | --- | --- | --- | --- | --- | --- | --- | --- | --- | --- | --- | --- | --- | --- |
| Random sequence generation | Yes | Yes | Yes | Yes | ? | ? | Yes | Yes | Yes | Yes | Yes | ? | ? | Yes | Yes | Yes | Yes | Yes | ? |
| Allocation concealment | ? | ? | ? | Yes | ? | ? | Yes | Yes | Yes | Yes | Yes | Yes | Yes | Yes | ? | Yes | Yes | Yes | Yes |
| Blinding of participants and personnel | ? | ? | ? | Yes | Yes | Yes | Yes | Yes | Yes | Yes | Yes | Yes | No | Yes | ? | Yes | Yes | Yes | Yes |
| Blinding of outcome assessment | Yes | Yes | Yes | Yes | Yes | Yes | Yes | Yes | Yes | Yes | Yes | Yes | Yes | Yes | Yes | Yes | Yes | Yes | Yes |
| Incomplete outcome data addressed | ? | ? | ? | ? | ? | ? | ? | ? | ? | ? | ? | ? | ? | ? | ? | ? | ? | Yes | Yes |
| Selective reporting | Yes | Yes | Yes | Yes | Yes | Yes | Yes | Yes | ? | Yes | Yes | Yes | Yes | Yes | Yes | Yes | Yes | Yes | Yes |
| Free of other bias | Yes | Yes | Yes | Yes | Yes | Yes | Yes | Yes | Yes | No | Yes | Yes | Yes | Yes | Yes | Yes | Yes | Yes | Yes |
| Risk of bias | High | High | High | Low | High | High | Low | Low | Moderate | High | Low | Moderate | Moderate | Low | High | Low | Low | Low | Low |
| Level of evidence | 1 | 1 | 1 | 1 | 1 | 1 | 1 | 1 | 1 | 1 | 1 | 1 | 1 | 1 | 1 | 1 | 1 | 1 | 1 |

Each paper was assessed on level of evidence and as either having a low (Yes), high (No), or unclear (?) risk of bias according to the *Cochrane Handbook [*[*20*](#_ENREF_20)*]*; moderate if one domain is scored as susceptible to bias or if 2 domains are scored as unclear; high if ≥ 2 domains are scored as susceptible to bias, if ≥ 3 or more of the domains are scored as unclear, or if one domain is scored as susceptible to bias and 2 domains are scored as unclear.

1. Agurs-Collins TD, Kumanyika SK, Ten Have TR, Adams-Campbell LL: **A randomized controlled trial of weight reduction and exercise for diabetes management in older African-American subjects**. *Diabetes Care* 1997, **20**(10):1503-1511.

2. Batik O, Phelan EA, Walwick JA, Wang G, LoGerfo JP: **Translating a community-based motivational support program to increase physical activity among older adults with diabetes at community clinics: a pilot study of Physical Activity for a Lifetime of Success (PALS)**. *Prevention Chronic Disease* 2008, **5**(1):A18.

3. Clark F, Jackson J, Carlson M, Chou CP, Cherry BJ, Jordan-Marsh M, Knight BG, Mandel D, Blanchard J, Granger DA *et al*: **Effectiveness of a lifestyle intervention in promoting the well-being of independently living older people: Results of the Well Elderly 2 Randomised Controlled Trial**. *Journal of Epidemiology and Community Health* 2012, **66**(9):782-790.

4. Fernandez S, Scales KL, Pineiro JM, Schoenthaler AM, Ogedegbe G: **A senior center-based pilot trial of the effect of lifestyle intervention on blood pressure in minority elderly people with hypertension**. *Journal of the American Geriatrics Society* 2008, **56**(10):1860-1866.

5. Fried LP, Carlson MC, Freedman M, Frick KD, Glass TA, Hill J, McGill S, Rebok GW, Seeman T, Tielsch J *et al*: **A social model for health promotion for an aging population: initial evidence on the Experience Corps model**. *Journal of urban health : bulletin of the New York Academy of Medicine* 2004, **81**(1):64-78.

6. Geller KS, Mendoza ID, Timbobolan J, Montjoy HL, Nigg CR: **The Decisional Balance Sheet to Promote Healthy Behavior Among Ethnically Diverse Older Adults**. *Public Health Nurs* 2012, **29**(3):241-246.

7. Holland SK, Greenberg J, Tidwell L, Malone J, Mullan J, Newcomer R: **Community-based health coaching, exercise, and health service utilization**. *Journal of Aging and Health* 2005, **17**(6):697-716.

8. Juang C, Knight BG, Carlson M, Schepens Niemiec SL, Vigen C, Clark F: **Understanding the Mechanisms of Change in a Lifestyle Intervention for Older Adults**. *Gerontologist* 2018, **58**(2):353-361.

9. Keller CS, Cantue A: **Camina por Salud: walking in Mexican-American women**. *Applied Nursing Research* 2008, **21**(2):110-113.

10. Kim BH, Glanz K: **Text messaging to motivate walking in older african americans: A randomized controlled trial**. *American Journal of Preventive Medicine* 2013, **44**(1):71-75.

11. Kim KB, Han HR, Huh B, Nguyen T, Lee H, Kim MT: **The effect of a community-based self-help multimodal behavioral intervention in Korean American seniors with high blood pressure**. *American Journal of Hypertension* 2014, **27**(9):1199-1208.

12. Palta P, Page G, Piferi RL, Gill JM, Hayat MJ, Connolly AB, Szanton SL: **Evaluation of a mindfulness-based intervention program to decrease blood pressure in low-income African-American older adults**. *Journal of urban health : bulletin of the New York Academy of Medicine* 2012, **89**(2):308-316.

13. Parisi JM, Kuo J, Rebok GW, Xue Q-L, Fried LP, Gruenewald TL, Huang J, Seeman TE, Roth DL, Tanner EK *et al*: **Increases in lifestyle activities as a result of experience Corps® participation**. *Journal of urban health : bulletin of the New York Academy of Medicine* 2015, **92**(1):55-66.

14. Qi BB, Resnick B, Smeltzer SC, Bausell B: **Self-efficacy program to prevent osteoporosis among Chinese immigrants: a randomized controlled trial**. *Nursing research* 2011, **60**(6):393-404.

15. Rejeski WJ, Spring B, Domanchuk K, Tao H, Tian L, Zhao L, McDermott MM: **A group-mediated, home-based physical activity intervention for patients with peripheral artery disease: effects on social and psychological function**. *J Transl Med* 2014, **12**:29-29.

16. Resnick B, Luisi D, Vogel A: **Testing the Senior Exercise Self-efficacy Project (SESEP) for use with Urban dwelling minority older adults**. *Public Health Nurs* 2008, **25**(3):221-234.

17. Sun WY, Dosch M, Gilmore GD, Pemberton W, Scarseth T: **Effects of a Tai Chi Chuan program on Hmong American older adults**. *Educational Gerontology* 1996, **22**(2):161-167.

18. Wolf RL, Lepore SJ, Vandergrift JL, Basch CE, Yaroch AL: **Tailored telephone education to promote awareness and adoption of fruit and vegetable recommendations among urban and mostly immigrant black men: a randomized controlled trial**. *Preventive Medicine* 2009, **48**(1):32-38.

19. Yeom HA, Fleury J: **A Motivational Physical Activity Intervention for Improving Mobility in Older Korean Americans**. *Western Journal of Nursing Research* 2014, **36**(6):713-731.

20. Green S, Higgins J: **Cochrane handbook for systematic reviews of interventions**. In*.*: Version; 2005.
