# Supplementary material for: Behaviour change interventions to promote health and well-being among older migrants: A systematic review
Source: PLoS One. 2022 Jun 16;17(6):e0269778. doi: 10.1371/journal.pone.0269778 (PMC9202883; doi:10.1371/journal.pone.0269778)
Supplement: S4 Table — (DOCX) [file pone.0269778.s004.docx]

**S4 Table: Assessment of quality for included non-controlled intervention studies based on Newcastle-Ottawa Scale**

|  |  | Beissner, K. (2012) [1] | Collins, C.C. (2006) [2] | Dogra, S. (2015) [3] | Emery-Tilburcio, E.(2017) [4] | Goldfinger, J.Z. (2008) [5] | Hau, C. (2016) [6] | Hooker, S.P. (2011) [7] | Jih, J. (2016) [8] | Lu, Y. (2014) [9] | Manson, J. (2013) [10] | Manson, J. (2013) [11] | Melchior, M.A. (2013) [12] | Parker, S.J. (2011) [13] | Sin, M.K. (2005) [14] | Taylor-Piliae, R.E. (2006) [15, 16] | Wilcox, S. (2006) [17] | Wilcox, S. (2008) [18] | Yan, T. (2009) [19] | Yan, T. (2009) [20] |
| --- | --- | --- | --- | --- | --- | --- | --- | --- | --- | --- | --- | --- | --- | --- | --- | --- | --- | --- | --- | --- |
| **Selection (max 4 points)** | 1a) Group truly representative | No | No | No | No | No | No | No | No | No | Yes | Yes | No | No | No | No | Yes | Yes | Yes | Yes |
|  | 1c) Selected group of users | Yes | No | Yes | Yes | Yes | Yes | Yes | Yes | Yes | No | No | Yes | Yes | Yes | Yes | No | No | No | No |
|  | 1d) No description of group | No | Yes | No | No | No | No | No | No | No | No | No | No | No | No | No | No | No | No | No |
|  | 2a) Control group from same community | Yes | Yes | Yes | Yes | Yes | Yes | Yes | Yes | Yes | Yes | Yes | Yes | Yes | Yes | Yes | Yes | Yes | Yes | Yes |
|  | 3a) Secure record | Yes | Yes | Yes | Yes | Yes | Yes | Yes | Yes | Yes | Yes | Yes | Yes | Yes | Yes | Yes | Yes | Yes | Yes | Yes |
|  | 4) Outcome of interested not present at start | No | No | No | No | No | No | No | No | No | No | No | No | No | No | No | No | No | No | No |
| **Comparability (max 2 points)** | 1a) Important factor controlled | Yes | No | No | No | No | Yes | No | Yes | No | No | No | No | Yes | No | No | Yes | Yes | Yes | Yes |
|  | 1b) Additional factor controlled | No | No | No | No | No | No | No | No | No | No | No | No | No | No | No | No | No | No | No |
| **Outcome (max 3 points)** | 1a) Structured interview | Yes | Yes | Yes | Yes | No | Yes | Yes | Yes | Yes | Yes | Yes | Yes | Yes | Yes | Yes | Yes | Yes | Yes | Yes |
|  | 1c) Self-report | Yes | No | No | No | Yes | No | No | No | No | No | No | No | No | No | No | No | No | No | No |
|  | 2) Follow-up long enough | Yes | Yes | Yes | Yes | Yes | Yes | Yes | Yes | Yes | Yes | Yes | Yes | Yes | Yes | Yes | Yes | Yes | Yes | Yes |
|  | 3b) Complete follow-up | Yes | Yes | Yes | Yes | Yes | Yes | Yes | Yes | Yes | Yes | Yes | Yes | Yes | Yes | Yes | Yes | Yes | Yes | Yes |
|  | Risk of bias | 7 = low | 6 = moderate | 6 = moderate | 6 = moderate | 6 = moderate | 7 = low | 6 = moderate | 7 = low | 6 = moderate | 6 = moderate | 6 = moderate | 6 = moderate | 7 = low | 6 = moderate | 6 = moderate | 7 = low | 7 = low | 7 = low | 7 = low |
|  | Level of evidence | 2 | 2 | 2 | 2 | 2 | 2 | 2 | 2 | 2 | 2 | 2 | 2 | 2 | 2 | 2 | 2 | 2 | 2 | 2 |

Each paper was assessed on level of evidence and as having a high risk of bias (0-3 points), moderate risk of bias (4-6 points) or low risk of bias (7-9 points), according to the Newcastle- Ottawa Scale [21]

1. Beissner K, Parker SJ, Henderson Jr CR, Pal A, Iannone L, Reid MC: **A cognitive-behavioral plus exercise intervention for older adults with chronic back pain: race/ethnicity effect?** *Journal of Aging and Physical Activity* 2012, **20**(2):246-265.

2. Collins CC, Benedict J: **Evaluation of a community-based health promotion program for the elderly: lessons from Seniors CAN**. *American Journal of Health Promotion* 2006, **21**(1):45-48.

3. Dogra S, Shah S, Patel M, Tamim H: **Effectiveness of a Tai Chi intervention for improving functional fitness and general health among ethnically diverse older adults with self-reported arthritis living in low-income neighborhoods: a cohort study**. *Journal of Geriatric Physical Therapy* 2015, **38**(2):71-77.

4. Emery-Tiburcio EE, Mack L, Lattie EG, Lusarreta M, Marquine M, Vail M, Golden R: **Managing Depression among Diverse Older Adults in Primary Care: The BRIGHTEN Program**. *Clinical Gerontologist* 2017, **40**(2):88-96.

5. Goldfinger JZ, Arniella G, Wylie-Rosett J, Horowitz CR: **Project HEAL: Peer education leads to weight loss in harlem**. *Journal of Health Care foor the Poor and Underserved* 2008, **19**(1):180-192.

6. Hau C, Reid KF, Wong KF, Chin RJ, Botto TJ, Eliasziw M, Bermudez OI, Fielding RA: **Collaborative evaluation of the healthy habits program: An effective community intervention to improve mobility and cognition of Chinese older adults living in the U.S**. *Journal Nutrition Health and Aging* 2016, **20**(4):391-397.

7. Hooker SP, Harmon B, Burroughs EL, Rheaume CE, Wilcox S: **Exploring the feasibility of a physical activity intervention for midlife African American men**. *Health Education Research* 2011, **26**(4):732-738.

8. Jih J, Le G, Woo K, Tsoh JY, Stewart S, Gildengorin G, Burke A, Wong C, Chan E, Fung LC *et al*: **Educational Interventions to Promote Healthy Nutrition and Physical Activity Among Older Chinese Americans: A Cluster-Randomized Trial**. *American Journal of Public Health* 2016, **106**(6):1092-1098.

9. Lu Y, Dipierro M, Chen L, Chin R, Fava M, Yeung A: **The evaluation of a culturally appropriate, community-based lifestyle intervention program for elderly Chinese immigrants with chronic diseases: a pilot study**. *Journal of public health (Oxford, England)* 2014, **36**(1):149-155.

10. Manson J, Ritvo P, Ardern C, Weir P, Baker J, Jamnik V, Tamim H: **Tai Chi's Effects on Health-Related Fitness of Low-Income Older Adults**. *Canadian Journal on Aging* 2013, **32**(3):270-277.

11. Manson J, Rotondi M, Jamnik V, Ardern C, Tamim H: **Effect of tai chi on musculoskeletal health-related fitness and self-reported physical health changes in low income, multiple ethnicity mid to older adults**. *BMC Geriatrics* 2013, **13**:114.

12. Melchior MA, Seff LR, Bastida E, Albatineh AN, Page TF, Palmer RC: **Intermediate outcomes of a chronic disease self-management program for Spanish-speaking older adults in South Florida, 2008-2010**. *Prevention Chronic Disease* 2013, **10**:E146.

13. Parker SJ, Vasquez R, Chen EK, Henderson CR, Jr., Pillemer K, Robbins L, Reid MC: **A comparison of the arthritis foundation self-help program across three race/ethnicity groups**. *Ethnicity and Disease* 2011, **21**(4):444-450.

14. Sin MK, Belza B, LoGerfo J, Cunningham S: **Evaluation of a community-based exercise program for elderly Korean immigrants**. *Public Health Nurs* 2005, **22**(5):407-413.

15. Taylor-Piliae RE, Haskell WL, Sivarajan Froelicher E: **Hemodynamic responses to a community-based Tai Chi exercise intervention in ethnic Chinese adults with cardiovascular disease risk factors**. *European Journal of Cardiovascular Nursing: journal of the Working Group on Cardiovascular Nursing of the European Society of Cardiology* 2006, **5**(2):165-174.

16. Taylor-Piliae RE, Haskell WL, Waters CM, Froelicher ES: **Change in perceived psychosocial status following a 12-week Tai Chi exercise programme**. *Journal of Advanced Nursing* 2006, **54**(3):313-329.

17. Wilcox S, Dowda M, Griffin SF, Rheaume C, Ory MG, Leviton L, King AC, Dunn A, Buchner DM, Bazzarre T *et al*: **Results of the first year of active for life: translation of 2 evidence-based physical activity programs for older adults into community settings**. *American Journal of Public Health* 2006, **96**(7):1201-1209.

18. Wilcox S, Dowda M, Leviton LC, Bartlett-Prescott J, Bazzarre T, Campbell-Voytal K, Carpenter RA, Castro CM, Dowdy D, Dunn AL *et al*: **Active for Life. Final Results from the Translation of Two Physical Activity Programs**. *American Journal of Preventive Medicine* 2008, **35**(4):340-351.

19. Yan T, Wilber KH, Aguirre R, Trejo L: **Do sedentary older adults benefit from community-based exercise? results from the active start program**. *Gerontologist* 2009, **49**(6):847-855.

20. Yan T, Wilber KH, Wieckowski J, Simmons WJ: **Results from the healthy moves for aging well program: Changes of the health outcomes**. *Home Health Care Services Quarterly* 2009, **28**(2-3):100-111.

21. Peterson J, Welch V, Losos M, Tugwell P: **The Newcastle-Ottawa scale (NOS) for assessing the quality of nonrandomised studies in meta-analyses**. *Ottawa: Ottawa Hospital Research Institute* 2011.
