# Supplementary material for: Behaviour change interventions to promote health and well-being among older migrants: A systematic review
Source: PLoS One. 2022 Jun 16;17(6):e0269778. doi: 10.1371/journal.pone.0269778 (PMC9202883; doi:10.1371/journal.pone.0269778)
Supplement: S5 Table — (DOCX) [file pone.0269778.s005.docx]

## **S5 Table: Study characteristics of included trials**

| **Author (year) Location** | **Study design** | **Sample characteristics**  - Subjects - Age (mean) - Ethnicity - Gender (% female) - Definition migrant | **Health behaviour** | **Intervention**  - Setting  - Content - Format - Duration - Total contact hours (h) | **BCT clusters BC theory/model** | **Cultural awareness** | **Follow-up** | **Outcome considered in the review** | **Effect size** | **Level of evidence Susceptibility to bias** |
| --- | --- | --- | --- | --- | --- | --- | --- | --- | --- | --- |
| Agurs-Collins, T.D. (1997) [1] Washington, D.C., United States | RCT | IG n=32, CG n=32 **Age**: IG 62.4 years (5.9) CG 61.0 years (5.7) **Ethnicity**: African American n=64 **Female**  76.6% **Definition migrant**: Nm  Overweight African American with non-insulin dependent diabetes | Physical activity  Diet | **Setting**: Urban hospital **IG:** Nutrition education on relationship of food and meals and of physical activity to body weight and to insulin activity and blood glucose (g) + Exercise; low-impact aerobic activity (g) + One individual diet counseling^[[1]](#footnote-1)^ session (i) + Additional information and support in sharing and problem-solving format (g) **CG:** (usual care) one class, related to methods of glycemic control (g) + two mailings of nutrition information (i) **Provider**: Dietitian **Duration**: 6 months **Total contact:** IG 37.5h  CG 1.5h | **BCT clusters**: 1 Goals and planning  3 Social support  4 Shaping knowledge  5 Natural consequences 6 Comparison of behaviour 8 Repetition and substitution 13 Identity  **BC theory/model**: Social action theory | Yes | 3 months  6 months | Weight BMI Blood pressure  HbA1c Total serum cholesterol  Physical activity levels *Scale for the Elderly Questionnaire Physical activity levels 1-week food frequency questionnaire*  Dietary intake | **Intervention vs. control at 3 weeks baseline** Anthropometrics: *Bodyweight***, *BMI***, *HbA1c***, *SBP*^#^, **DBP*^#^, *cholesterol*^#^   Health behaviour: *Physical activity*, percentage intake of;* *kilocalories**, *kcal* *fat***, *kcal saturated fat**, *kcal protein*^#^, *kcal carbohydrate**, *cholestero*l^#^  **Intervention vs. control at 6 weeks baseline** Anthropometrics: *Bodyweight***, *BMI***, *HbA1c***, *SBP*^#^, *DBP*^#^, *cholesterol*^#^   Health behaviour: *Physical activity*^#^, *percentage intake of; kilocalories*^#^*, kcal* *fat*^#^, *kcal* *saturated fat*^#^, *kcal protein*^#^, *kcal carbohydrate*^#^, *cholestero*l^#^ | Level of evidence: 1 Susceptibility to bias: High |
| Batik, O. (2008) [2] South Seattle, United States | RCT | **IG** n=135, **CG** n=170 **Age**: IG 73.6 years (7.9) CG 71.9 years (6.3) **Ethnicity**: Races other than white **Female** 68.5% Diabetic patients **Definition migrant**: Nm | Physical activity | **Setting**: Community-based **IG**: *Physical Activity for a Lifetime of Success^[[2]](#footnote-2)^* (PALS) (i);  Reviews of patient’s physical activity level and discuss the benefits of PA with the patient + Developing PA prescription in collaboration with participant  + Motivational support program delivered by older adult volunteers over the telephone to carry out a PA plan + Guide to local activity resources + Handouts about benefits of exercise, tips on safety and strength and balance exercise  **CG**: Handout about benefits of exercise, tips on safety, and strength and balance exercise + Guide to local activity resources **Provider**: Primary Care Provider, Older adult volunteers **Duration**: 6 months **Total contact**: Nm | **BCT clusters**: 1 Goals and palnning  2 Feedback and monitoring  3 Social support 4 Shaping knowledge  5 Natural consequences 9 Comparioson of outcomes | No | 6 months | Physical activity level *RAPA questionnaire* | **Intervention vs. control at 6 months baseline** Anthropometrics: *HbA1c*^#^  Health behaviour: *Sufficiently active*^#^ | Level of evidence: 1 Susceptibility to bias: High |
| Beissner, K. (2012) [3] New York City, United States | Pre-post design | **IG** n=69 **Age**: 75.57 years (7.92) **Ethnicity**: African American n=20, Hispanic n=25, Non-Hispanic White n=24, **Female** 81% **Definition migrant**: Individual criterion of race/ethnicity  Chronic, non-cancer-related back pain | Physical activity | **Setting**: Senior center **IG**: *Moving Past the Pain* (*MPP*) (g) Cognitive-behavioural exercise therapy  + Discussing challenges and success experienced by participants doing the homework exercises + Handout materials to highlight key take-home point and homework exercise **Provider**: Exercise expert **Duration**: 8 weeks **Total contact**: 12h | **BCT clusters**: 1 Goals and planning 3 Social support 4 Shaping knowledge  6 Comparison of behaviour 8 Repetition and substitution | Yes | 9 weeks | Pain-related disability *24-item Roland-Morris Disability Questionnaire (0-10)*  Pain intensity   Functional status *ADL score*  Level of social activity *Validated protocol*^[[3]](#footnote-3)^   Depressive symptoms *PHQ-9* | **9 weeks vs. baseline** Health behaviour:  Social activity (all **large**); African American d=0.77^#^, Hispanic d=1.72*, Non-Hispanic Whites d=7.79^#^ , all participants d=1.50^#^  Physical functioning:  *Pain-related disability* (all **large**);  African American d=-3.46***, Hispanic d=-6.54***, Non-Hispanic Whites d=-2.88***, all participants d=-7.10***  *Functional status*;  African American d=0.45 (**medium**)^#^, Hispanic d=2.43 (**large**)**, Non-Hispanic Whites d=0.76 (**medium**)^#^, all participants d=2.03 (**large**)***  Mental health and functioning: *Depressive symptoms*; African American d=2.15 (**large**)^#^, Hispanic d=-2.92 (**large**)*, Non- Hispanic Whites d=-1.30 (**large**)^#^, all participants d=-1.33  **(large**)^#^  Generic health and well-being: *Pain intensity*;  African American d=0.04 (**small**)^#^, Hispanic d=-5.61 (**large**)***, Non-Hispanic Whites d=-2.36 (**large**)^#^, all participants d=-4.51 (**large**)*** | Level of evidence: 2 Susceptibility to bias: Low |
| Clark, F. (2012) [4]  Juang, C. (2018) [5]  Los Angeles, United States | RCT | **IG** n=232, **CG** n=228 **Age**: 74.85 years (7.7) **Ethnicity**: Asian n=18, Black/ African American n=149, Hispanic or Latino n=92, White n=172, Other n=29  **Female** 65.9% **Definition migrant**: Nm | Physical activity | **Setting**: Homes or community settings **IG**: *Lifestyle Redesign* (i+g), occupational therapy Group sessions by occupational therapist concerning activity and health, time use and energy conservation, transport utilization, home and community safety, social relationships, cultural awareness, goal setting and changing routines and habits involving peer exchange, direct experience, personal exploration (g) + Individual sessions in homes or community setting (i) + Community outings (g)  **CG**: Wait list  **Provider**: Occupational therapist **Duration:** 6 months **Total contact**: 58h | **BCT clusters**: 1 Goals and planning 2 Feedback and monitoring  3 Social support 4 Shaping knowledge  6 Comparison of behaviour 8 Repetition and substitution  9 Comparison of outcomes 12 Antecedents | Yes | 6 months | Perceived physical health and well-being *SF-36v 2*  Social integration and connections *LSNS*  Activity *MAPA-f*  Depressive symptoms  *CES-D scale*  Life satisfaction *LSI-Z*  Immediate recall Delayed recall Recognition *Consortium*  *to Establish a Registry of Alzheimer’s Disease Word List Memory* task | **Intervention vs. control at 6 months** Physical functioning: (All **small**) *Physical function* d=0.09^#^, *physical composite* d=0.01^#^, *psychomotor spe*ed d=0.067**  Social functioning:  *Social function* d=0.22 (**small**)*,  *social connections*^#^, *activity frequency* d=0.44 (**small**)***  Mental health and functioning: (All **small**) *Mental health* d=0.14*, *mental composite* d=0.18**depression* d=-0.18*, *depressive symptoms* d=-0.16*, *immediate recall* d=-0.09*, *delayed recall* d=-0.005***, *recognition* d=-0.04**, *cognitive functioning* d=-0.03^#^  Generic health and well-being: B*odily pain* d=0.18*, *general health* d=0.001^#^, *vitality* d=0.18*, *life satisfaction* d=0.12*, | **Level of evidence**: 1 **Susceptibility to bias**: High |
| Collins C.C. (2006) [6] Nevada, United States | Pre-post design | **IG** n=339 **Age**: 72.20 years (8.64) **Ethnicity**: African American n=34, Asian American n=20, Latino n=48, Native American n=7, White n=230, **Female** 80% **Definition migrant**: Nm | Social functioning | **Setting**: Senior centers and senior housing developments. **IG**: *Seniors CAN* (g); Education sessions on nutrition and food, personal safety, financial strategies to manage limited resources, general wellness, and productive aging  + Emphasizing how information could be readily applied in life, encouraging participants to integrate one new idea or skill from each lesson + Sharing experiences  **Provider**: Peer educator  **Duration**: 4 months **Total contact**: 32h | **BCT clusters**: 4 Shaping knowledge  5 Natural consequences  6 Comparison of behaviour 8 Repetition and substitution  15 Self-belief | No | 4 months | Loneliness *UCLA Loneliness Scale*  Stress *PPS-10* | **16 weeks vs. baseline** Social functioning:  *Loneliness* d=-1.01 (**large**)***  Generic health and well-being: *Stress* d=-1.51 (**large**)*** | Level of evidence: 2 Susceptibility to bias: Moderate |
| Dogra, S. (2015) [7] Greater Toronto Area, Canada | Pre-post design | **IG** n=201 **Age**: 67.8 years (8.1) **Ethnicity**: Canada n=8, Caribbean n=9, Chinese n=37, Europe n=14, South Asian n=3, South America n=25, Other n=4 **Female** 77.2% **Definition migrant**: Born out-side Canada | Physical activity | **Setting**: Local center  **IG**: Tai Chi; 15 min Qigong, 45 min Yang-style Tai Chi (g) (advised to attend 2 classes) **Provider**: Professional Tai Chi master **Duration**: 4 months  **Total contact**: ~ 32h | **BCT clusters**: 4 Shaping knowledge  6 Comparison of behaviour 8 Repetition and substitution | Yes | 4 months | Muscular strength *Hand grip strength (kg) Timed up-and-go (s)*    Muscular endurance  *Arm curl (number of repetitions/30-second) Chair stand (number of repetitions/30-second)*  Flexibility *Sit-and-reach (cm)*  Mental and physical health   *SF-36* | **16 weeks vs. baseline**  Physical functioning: *Left hand grip strength* d=0.26 (**medium**)**, r*ight hand grip strength* d=0.14 (**small**)^#^, *combined hand grip strength* d=0.21 (**medium**)**, *timed up-and-go* d=-0.19 (**small; in favor CG**)**, *arm curl* d=0.56 (**medium**)***, *chair stand* d=0.69 (**medium**)***, *sit-and-reach* d=0.11 (**small**) ^#^, *overall physical component summary* d=0.32 (**small**)***, *physical functioning* d=0.37 (**small**)***, *role physical* d=0.12 (**small**) ^#^  Social functioning:  Social functioning d=-0.12 (**small; in favor CG**) ^#^  Mental health and functioning: Mental component summery d=0.023 (**small**) ^#^, mental health d= 0.25 (**small**)*  Generic health and well-being: *Bodily pain* d=0.17 (**small**) ^#^, *general health* d=0.21 (**small**)*, *vitality* d=0.31 (**small**)**, role emotional d=-0.059 (**small**) ^#^ | Level of evidence: 2 Susceptibility to bias: Moderate |
| Emery-Tilburcio, E. (2017) [8] Cook County, United States | Pre-post design | **IG** N n=131 **Age**: 67.4 years (6.7) **Ethnicity**: African American n=49, Hispanic n=38, Non-Hispanic White n=39 **Female** 80.9% **Definition migrant**: Nm  Older adults with depression | Depression management | **Setting**: Community-based senior centers  **IG**: *BRIGHTEN* (i) (Bridging Resources of a Geriatric Health Team via Electronic Networking), biopsychosocial evaluation + Recommendations for mental and overall health by *BRIGHTEN* team via secure email  + Developing person-centered treatment plan in collaboration with participant + Referral to treatment providers and community services + Follow-up calls to monitor adherence upon treatment plan **Provider**: BRIGHTEN team (psychologist, social worker,  and psychiatrist, as well as an occupational therapist,  physical therapist, dietitian, and chaplain) **Duration**: 6 months **Total contact**: Nm | BCT clusters: 1 Goals and planning 2 Feedback and monitoring  3 Social support)  9 Comparison of outcomes 12 Antecedents | Yes | 6 months | Mental and physical health  SF-12^[[4]](#footnote-4)^  Depression GDS-154 | **6 months vs. baseline**  Physical functioning: *Physical health composite* d=-0.08 (**small; in favor CG**)^#^  Mental health and functioning: *Mental health composite* d=0.47 (**medium**)***, *depression* d=-0.60 (**medium**)***  - No difference on outcome measures based on race/ethnicity | Level of evidence: 2 Susceptibility to bias: Moderate |
| Fernandez, S. (2008) [9] New York City, United States | RCT | **IG** n=35, **CG** n=30 **Age**: 72.29 years (6.92) **Ethnicity**: African American n=55, Black + Latino + Hispanic n=15 **Female** 53.8% **Definition migrant**: Self-identification as black, African American, Latino or Hispanic  Uncontrolled blood pressure^[[5]](#footnote-5)^ | Physical activity  Diet | **Setting**: Community-based senior center **IG:** Behavioural counseling sessions (g) + 'Booster' sessions on goal setting, self- monitoring, stimulus control, and problem solving strategies for adoption and maintenance of lifestyle modifications + Handouts corresponding with topic of the session were provided **CG**: Wait list **Provider**: Researcher(s) **Duration**: 2 months **Total contact**: 10h | BCT clusters: 1 Goals and planning  3 Social support 4 Shaping knowledge | No | 6 weeks 14 weeks | Blood pressure   Physical activity Diet *REAP* | **Intervention vs. control at 6 weeks** Anthropometrics: *SBP* d=-0.31 (**small**)**, *DBP* d=-0.21 (**small**)*  Health behaviour: *Physical activity* d=0.20 (**small**)^#^, *fruit intake* d=0.48 (**medium**)^#^, *vegetable intake* d=0.35 (**small**)^#^  **Intervention vs. control at 14 weeks** Anthropometrics: *SBP* d=-0.15 (**small**)**, *DBP* d=-0.24 (**small**)*  Health behaviour: *Physical activity* d=0.2 (**small**)^#^, f*ruit intake* d=0.56 (**medium**)^#^, *vegetable intake* d=0.64 (**medium**)* | Level of evidence: 1 Susceptibility to bias: High |
| Fried, L.P. (2004) [10] Baltimore, United States | RCT | **IG** n=70, **CG** n=58 **Age**: 60-86 years **Ethnicity**: African American n=122 Caucasian/other n=6  **Female** 91.7% **Definition migrant**: Nm | Social functioning | **Setting**: Public elementary school **IG:** *Experience Corps* (i+g) Participants placed in a given public elementary school to a) support literacy development for children in kindergarten through third grade, b) support library functions under the guidance of a librarian, c) teach children how to solve problems and play, d) enhance school attendance (i)  + Participants were trained in team building problem-solving, planning, and socializing (g) **CG**: waitlist  **Provider**: Nm **Duration**: One school year **Total contact**: ~615h | BCT clusters: 3 Social support 4 Shaping knowledge  8 Repetition and substitution 10 Reward and threat | No | 1 year | Physical activity *Number blocks walked/week Flights of stairs climbed/week Activity in kilocalories/week Strength (very good/excellent) Walking speed (m/s)*  Social networks and support *Who would check on you if sick One could depend on Seen in a typical week Could have used more emotional support in past year*  Cognitive functioning *Books and variety of materials read per month High (crossword puzzles), moderate (cooking), and low (tv) cognitive intensity activities engaged in outside the program over the prior month Hours spent watching television per day* | **Intervention vs. control at one year**  Health behaviour: *More active**, *number blocks walked*^#^, *flights of stairs climbed*^#^, a*ctivity*^#^  Physical functioning:  *Strength**, *walking speed****  Social functioning: *Who* *would check on you if sick**, *one could depend on*^#^, *seen in a typical week*^#^, *could have used more emotional support in past year*^#^ Mental health and functioning: *Books read*^#^, *high-intensity*^#^, *moderate- intensity*^#^, *low- intensity*^#^ | Level of evidence: 1 Susceptibility to bias: Moderate |
| Geller, K.S. (2012) [11] Hawaii | RCT | Physical activity n=12 Fruit and Vegetable n=9 **Age**: 72.24 years (11.84) **Ethnicity**: Caucasian n=4, Filipino n=4, , Hispanic n=1, Japanese n=5, Native American n=1, Native Hawaiian n=1, Others n=5  **Female** 76% **Definition migrant**: Nm | Physical activity  Diet | **Setting**: community housing **IG**: Balance sheet program (g) Group discussion on gains and losses associated with behaviour adaptions for physical activity or fruit and vegetable intake, both personal and social consequences  **CG:** PA vs. F&V and vice versa **Provider**: Nm **Duration**: One day **Total contact:** Nm | BCT clusters: 5 Natural consequences 6 Comparison of behaviour 9 Comparison of outcomes | No | ~2 weeks | Physical activity  *IPAQ* Fruit and vegetable intake  *NHNES* | **2 weeks vs. baseline** Health behaviour: *Moderate physical activity minutes* PA group d=-0.80 (**small; in favor CG**) *Fruit and vegetable intake* F&V group d=0.30 (**small**) | Level of evidence: 1 Susceptibility to bias: High |
| Goldfinger, J.Z. (2008) [12] Harlem, United States | Pre-post design | **IG** N = 26 **Age**: 68.3 years (10.0) **Ethnicity**: African American (all) **Female** 81% **Definition migrant**: Nm  Overweight or obese^[[6]](#footnote-6)^ | Physical activity Diet | **Setting**: Local church **IG**: *Project HEAL: Healthy Eating, Active Lifestyle* (i+g)  Education on activity and diet (g) + Weekly action plans (i), group feedback and support to inspire change and to model self-management and problem-solving + Two refresher classes  **Provider**: Peer **Duration**: 10 weeks **Total contact**: 12h | **BCT clusters**: 1 Goals and planning 2 Feedback and monitoring  3 Social support 5 Natural consequences  6 Comparison of behaviour 8 Repetition and substitution  13 Identity | Yes | 10 weeks 22 weeks 1 year | Diet Physical activity Sedentary time Perceived health related QoL Self-reported behaviour | **10 weeks vs. baseline** Health behaviour: *Physical activity* d=0.36 (**small**)^#^, *sedentary time* d=-0.44 (**small**)*, Intake of; *Total fat* d=-0.34 (**small**)*, *daily saturated* *fat* d=-0.29 (**small**)*, *daily cholesterol* d=-0.27 (**small**)*, *fruit and vegetable* d=0.13 (**small**) ^#^  **22 weeks vs. baseline** Health behaviour: *Physical activity* d=0.21 (**small**) ^#^, *sedentary time* d=-0.29 (**small**), Intake of; *Total fat* d=-0.17 (**small**)*, *daily saturated fat* d=-0.19 (**small**)*, *daily cholesterol* d=-0.14 (**small**)*, *fruit and vegetable* d=0.41 (**small**)*  **One year vs. baseline** Health behaviour: *Physical activity* d=0.08 (**small**)^#^, *sedentary time* d=-1.038 (**large**)***, Intake of; *Total fat* d=-0.38 (**small**)^#^, *daily saturated fat* d= -0.3 (**small**)^#^, *daily cholesterol* d=-0.27 (**small**)^#^, *fruit and vegetable* d=0.37 (**small**)*  Generic health and well-being: Health related QoL^#^ | Level of evidence: 2 Susceptibility to bias: Moderate |
| Hau, C. (2016) [13] Boston, United States | Pre-post design | **IG** n=50 **Age**: 68.4 years (6.2) **Ethnicity**: Chinese (all) **Female** Nm **Definition migrant**: Nm | Physical activity Diet | **Setting**: Wang Young Men’s Christian Association (community center) **IG**: *Healthy Habits Program* (i/g) Physical activity by utilizing available facilities, equipment and exercise classes offered at the Wang YMCA (i/g), asked to perform ≥3 times/week (i/g) + Counseling sessions involving discussions on topics related to healthy lifestyle: healthy diet, physical activity, disease management (g)  **Provider**: Nm **Duration**: 6 months **Total contact**: Nm | **BCT clusters**: 3 Social support  5 Natural consequences  13 Identity | Yes | 6 months | Nutrition Status  *MNA* Mobility limitations  *SPPB*  Muscular strength *Grip strength*  *Isometric knee strength*   Depressive symptoms  *PHQ-9*   Disability in ADL  *WHODAS-I*  Cognition *MMSE TMT CWT*^[[7]](#footnote-7)^ | **6 months vs. baseline** Health behaviour: Nutrition status d=0.27 (**small**)^#^  Physical functioning:   *SPPB* d=0.4 (**small**)**, *habitual gait speed* d=0.65 (**medium**)***, *5-repeated chair rises* d=-0.47 (**small; in favor CG**)**, *maximal gait speed* d= 0.57 (**medium**)***, *grip strength* d=-0.061 (**small; in favor CG**)^#^, *left* *isometric knee strength* d=0.17 (**small**)^#^, *right* *isometric knee strength* d=0.19 (**small**)^#^, *ADL disability* d=-0.20 (**small**)**  Mental health and functioning: *Depressive symptoms* d=-0.26 (**small**)*, *MMSE* d=0.71(**medium**)***, *TMT part A* d=-0.36 (**small; in favor CG**)***, TMT part B d=0.01 (**small**)^#^, *complex walking* d=-0.34 (**small; in favor CG**)* | Level of evidence: 2 Susceptibility to bias: Low |
| Holland, S.K. (2005) [14] California, United States | RCT | **IG** n=255, **CG** n=249 **Age**: IG 73.1 years (4.9)  CG 72.9 years (5.0) **Ethnicity**: White n=204 (both groups) Non-White IG n=51, CG n=45 **Female** IG 60%, CG 50% **Definition migrant**: Nm  ≥1 chronic health conditions | Physical activity | **Setting**: Community-based  **IG**: *Health Matters Program* (*HMP*)^[[8]](#footnote-8)^ (i) Health assessment  + Client-developed health action plan + Health coaching  + Health education, counseling and medication management coaching (upon request)  + Monitoring of adherence and progress of health action plan by follow-up visits and phone contacts + Fitness program, *Lifetime Fitness* + Referral to community programs + Monthly newsletter with health information and list of *Health Matters* and community-sponsored classes + Availability of social worker **CG:** No access to *Health Matters* programs, after 12 months eligible for *Lifetime Fitness program* **Provider:** Nurse health coach, social worker and geriatrician **Duration**: 6 months **Total contact:** 11h | **BCT clusters**: 1 Goals and planning  2 Feedback and monitoring 3 Social support  5 Natural consequences  12 Antecedents  15 Self-belief | No | One year | Health status risk indicators BMI  *Health distress (0-5) Functional difficulties (0-3) Health limitations (0-4) Fatigue (0-10) Pain (0-10)*   Physical activity  *Self-reported  Aerobic activity Stretching*  Social activity *Social visits Telephone contacts  Formal meetings*  Depression *GDS* | **Intervention vs. control at one year** Anthropometrics: *BMI*^#^  Health behaviour: *Aerobic activity**, social visits*^#^, *telephone contacts*^#^*, formal meetings*^#^  Physical functioning: *Functional difficulties*^#^, *stretching***  Mental health and functioning: *Depression*^#^  Generic health and well-being: *Health distress*^#^, *fatigue*^#^, *pain*^#^, *health limitations*^#^, | Level of evidence: 1 Susceptibility to bias: Low |
| Hooker, S.P. (2011) [15]  Columbia, United Sates | Quasi-experiment | **IG** n=25 **Age**: 54.6 years (5.9) **Ethnicity**: African American (all) **Female** 0% **Definition migrant**: Nm  Inactive or irregularly active African American men | Physical activity Social functioning | **Setting**: Community wellness center **IG**: Sessions on benefits of PA, overcoming barriers, gaining social support, setting goals, self-monitoring, and fitting PA into a daily routine (g) + Discussions embracing problem solving, accountability and camaraderie (g) + Brief PA demonstrations on stretching, resistance training, and brisk walking were provided during the program (g) **Provider**: Trained facilitators **Duration**: 2 months **Total contact**: 24h | **BCT clusters**: 1 Goals and planning 2 Feedback and monitoring 3 Social support 4 Shaping knowledge  5 Natural consequences  6 Comparison of behaviour 8 Repetition and substitution  **BC theory/model**: Social cognitive theory | Yes | 2 months | Bodyweight (kg) BMI   Lower-body leg strength *Chair stand*  Flexibility *Sit-and-reach*  Aerobic fitness *Rickport Fitness (1 mile) Walking test*   Physical activity *CHAMPS PA questionnaire*  Social support | **8 weeks vs. baseline** Anthropometrics: *Bodyweight* d=-0.06 (**small**)^#^*, BMI* d=-0.11 (**small**)^#^  Health behaviour: MVPA d=1.05 (**large**)**, *overall PA* 0.78 (**large**)**  Physical functioning: *Chair stand* d=0.93 (**large**)***, *sit and reach* d=0.57 (**medium**)***, *aerobic fitness* d=0.89 (**large**)***  Social functioning: *Social support from family* d=0.83 (**large**)***, *friends* d=1.53 (**large**)*** | Level of evidence: 2 Susceptibility to bias: Moderate |
| Jih, J. (2016) [16] San Francisco, United States | Cluster RCT | **IG** n=365, **CG** n=360 **Age**: IG 61.7 years (7.0) CG 62.8 years (6.8) **Ethnicity**: Chinese (all) **Female** IG 79.2%, CG 83.1% **Definition migrant**: Self-identifying as Chinese or Chinese American | Physical activity Diet | **Setting**: Nm **IG**: Nutrition and physical activity lectures^[[9]](#footnote-9)^ (g) Lecture 1: How to Eat Smart and Be Active; basic NPA education. Lecture 2: Disease Prevention and Health Promotion; general information on hypertension, hypercholesterolemia, and diabetes mellitus; how healthy NPA reduces the risks of those conditions  + Chinese-language print materials + Two maintenance telephone calls, one month after lecture (reminder next lecture) + Printed lecture handouts and nutrition brochure  **CG**: Same print materials + Two small group education sessions on colorectal cancer and  + Two phone calls about colorectal cancer, one month after each small group session **Provider**: Lay health worker **Duration**: 3 months **Total contact**: 3h | **BCT clusters**:  5 Natural consequences 12 Antecedents | Yes | 3 months | Physical activity  *Duration of at least moderate intensity physical activity during previous week (150 minutes)*  Diet *Number servings of vegetables (≥5 per day) and fruit (≥4 per day) eaten previous day* | **Intervention vs. control at 3 months** Health behaviour: *Physical activity* d=0.13 (**small**)^#^, *vegetable intake* d=0.94 (**large**)***, *fruit intake* d=0.31 (**small**)** | Level of evidence: 1 Susceptibility to bias: Low |
| Keller, C. (2008) [17] United States | Two group- RCT | **Group I** N = 11 **Group II** N = 7 **Age**: Group I 56.5 (6.4)  Group II 53.5 (5.8) **Ethnicity**: Hispanic (all) **Female** 100% Postmenopausal, obese, sedentary^[[10]](#footnote-10)^ Mexican-American women **Definition migrant**: Nm | Physical activity  Diet | **Setting**: Neighborhood of participant and community center **IG**: *Camina por Salud* (i+g); 30 minutes walking, Group I: 3 days/ week Group II: 5 days/week; weekly planned (g) + Walking plan designed by promotora (i) + Creating walking summary (i) + Education sessions; heart-health information, including nutritional education directed towards low-fat dietary intake and low-fat food preparation methods (g), monthly  + Social time with snacks (g) + Additional routes mapped out, surrounding the community center (i) **Provider**: Lay health worker (promotora) **Duration**: 9 months **Total contact**: Group I ~63h, Group II ~99h | **BCT clusters**: 1 Goals and planning 2 Feedback and monitoring 3 Social support 5 Natural consequences 8 Comparison of behaviour 10 Reward and threat 12 Antecedents | Yes | 3 months 9 months | Bodyweight BMI  Total serum cholesterol LDL-C Triglycerides   Social support Friend *Support for Exercise Scale and Family Support for Exercise Scale* | **12 weeks post-baseline**: Anthropometrics: *Bodyweight* (both **small**) Group I d=-0.37 Group II d=0.20  *BMI* (both **small**) Group I d=-0.34*, Group II d=0.26** *Cholesterol* (both **small**) Group I d=0.28, Group II d=-0.21  *HDL-C* (both **small**) Group I d=-0.10, Group II d=0.26  *LDL-C* (both **small**) Group I d=0.26, Group II d=-0.05  *Triglycerides* (both **medium**) Group I d=0.5, Group II d=-0.52  **36 weeks post-baseline**: Anthropometrics:  *Bodyweight* (both **large**) Group I d=1.16, Group II d=0.91  *BMI* (both **large**) Group I d=-1.08***, Group II d=-0.91^#^  *Cholesterol* Group I d=0.67 (**medium**), Group II d=0.03 (**small**) *HDL-C* (both **medium**) Group I d=0.51, Group II d=0.60  *LDL-C*  Group I d=0.47 (**medium**), Group II d=-0.30 (**small**) *Triglycerides* (both **small**) Group I d=-0.16, Group II d=0.19  **Group I vs. Group II at 36 weeks** Anthropometrics: *BMI**, body*weight****, *serum* *cholesterol*^#^, *LDL-C*^#^, *triglyceride*^#^ | Level of evidence: 1 Susceptibility to bias: Moderate |
| Kim, B.H. (2013) [18] United States | RCT | **IG** n=26, **CG** n=15 **Age**: 60-85 years **Ethnicity**: African American (all) **Female** 80.6% **Definition migrant**: Nm | Physical activity | **Setting**: Urban setting **IG**: Motivational text messages via telephone (i), 3 times a day, 3 days a week + Walking instructional manual + Pedometer **Provider**: Google Voice **Duration**: 6 weeks **Total contact**: Nm | **BCT clusters**: 1 Goals and planning  2 Feedback and monitoring  3 Social support 5 Natural consequences 7 Associations 8 Repetition and substitution  13 Identity | No | 6 weeks | Step count Pedometer  Perceived activity level *LTEQ* | **Intervention vs. control at 6 weeks** Health behaviour: *Number of steps* d= 1.19 (**large**)*, *perceived activity level*  d=0.77 (**large**)* | Level of evidence: 1 Susceptibility to bias: High |
| Kim, K.B. (2014) [19] Baltimore, United States | RCT | **IG** n=184, **CG** n=185 **Age**: 70.9 years (5.3) **Ethnicity**: Korean American (all) **Female** 69.9% **Definition migrant**: Nm  High blood pressure | Physical activity  Diet | **Setting**: Korean American churches and senior centers **IG**: Education and training on health literacy training and high blood pressure (HBP) management (g) + Twice a day BP measurement at home (i) + Monthly telephone counseling for 12 months (i) **Provider**: Registered nurse **Duration**: 12 months **Total contact**: Nm | **BCT clusters**: 1 Goals and planning 2 Feedback and monitoring 3 Social support 4 Shaping knowledge 5 Natural consequences  **BC theory/model**: Social cognitive theory | Yes | 6 months  12 months | Blood pressure   Depressive symptoms *PHQ-9* | **Intervention vs. control at 6 months** Anthropometrics : *SBP* d=-0.37 (**small**)***, *DBP* d=-0.27 (**small**)***  Mental health and functioning: *Depressive symptoms* d=0.03 (**small**)*  **Intervention vs. control at 12 months** Behavioural : *SBP* d=-0.37 (**small**)***, *DBP* d=-0.36 (**small**)**  Mental health and functioning: *Depressive symptoms* d=-0.22 (**small**)* | Level of evidence: 1 Susceptibility to bias: Low |
| Lu, Y. (2014) [20] Boston, United States | Pre-post design | **IG** n=99 **Age**: 70.6 years (5.8) **Ethnicity**: Chinese Americans **Female** 58% **Definition migrant**: Nm  At least one chronic disease/condition | Physical activity  Diet | **Setting**: Wang Young Men’s Christian Association (community center) **IG**: *Healthy Habits Program* (i/g) Physical activity; utilizing available facilities, equipment and exercise classes offered at the Wang YMCA, asked to perform ≥3 times/week (i/g) + Group education program; maintaining healthy diet, exercise and management of diabetes, hypertension and arthritis, weekly 1h (g) + Share personal experience **Provider**: YMCA care manager **Duration**: Nm **Total contact**: Nm | **BCT clusters**: 3 Social support  5 Natural consequences 6 Comparison of behaviour | Yes | 3 months  6 months | Bodyweight Blood pressure  Balance *One-leg stance*  Lower body strength *Chair stand test*  Disability in ADL  *WHODAS-II*  Difficulties in seeking, hearing, walking, memory, self-care and communication *UN Washington Group Disability scale*  Depressive symptoms *PHQ-9* | **3 months vs. baseline** Anthropometrics: *Bodyweight* d=-0.20 (**small**), *BMI* -0.12 (**small**), *SBP* d=-0.36 (**medium**), *DBP* d=-0.25 (**small**)  Physical functioning: *Left one-leg stance* d=0.31 (**small**), *right one-leg stance* d= 0.21 (**small**), *chair stand* d=0.46 (**small**), *ADL disability* d=0.03 (**small**), *disability* d=-0.03 (**small**)  Mental health and functioning: *Depressive symptoms* d=-0.20 (**small**)  **6 months vs. baseline** Anthropometrics:  *Bodyweight* d=-0.10 (**small**)***, BMI -0.12 (**small**)***, SBP d=-0.50 (**medium**)***, DBP d=-0.41 (**small**)***  Physical functioning: *Left one-leg stance* d=0.38 (**small**)***, *right one-leg stance* d=0.31 (**small**)***, *chair stand* d=0.83 (**large**)***, *ADL impairment* d=0.14 (**small**)^#^, *disability* d=-0.11 (**small**)^#^  Mental health and functioning: *Depressive symptoms* d=-0.19 (**small**)** | Level of evidence: 2 Susceptibility to bias: Moderate |
| Manson, J. (2013) [21] Toronto, Canada | Pre-post design | **IG** n=78 **Age**: 55-64 years n=11 65-74 years n=42 75+ years n=25 **Ethnicity**: Argentinian, Ecuadorian & Brazilian n=16; Guyanese, Trinidadian & West Indian n= 45; Hindu, East Indian, Sri Lankan & Jamaican n= 17 **Female** 80.4% **Definition migrant**: Nm | Physical activity | **Setting**: Community housing  **IG**: Tai Chi (g) 15 min Qigong, followed by 45 min Yang-style Tai Chi, 2 classes per week (advised to attend) **Provider**: Tai Chi master **Duration**: 4 months  **Total contact**: 12h | **BCT clusters**: 4 Shaping knowledge  6 Comparison of behaviour 8 Repetition and substitution | No | 4 months | BMI Resting BP  Musculoskeletal upper and lower body measures *Grip strength Arm curl test  Chair and stand  Partial curl- ups*  *Sit and reach  Timed 8-foot up and go test* | **16 weeks vs. baseline** Anthropometrics: *BMI* d=-0.08 (**small**)^#^, SBP d=0.02 (**small**)^#^, *DBP* d=0.01 (**small**)^#^  Physical functioning**:** *Overall grip strength* d=0.35 (**small**)**, *arm curl test* d=0.73 (**medium**)**, *chair* *stand t*est d=0.71 (**medium**)**, *partial* *curl-ups* d=0.51 (**medium**)**, *sit and reach* d=0.18 (**small**)^#,^ *8-foot up and go test* d=-0.13 (**small; in favor CG**)^#^ | Level of evidence: 2 Susceptibility to bias: Moderate |
| Manson, J. (2013) [22] Greater Toronto Area, Canada | Pre-post design | **IG** n=209 **Age**: 68.1 years (8.62) **Ethnicity**: American n=54, Canadian n=13, Chinese n=74, European n=33, South Caribbean n=13, South Asian n=10, Other n=8 **Female** 79.9% **Definition migrant**: Nm | Physical activity | **Setting**: Community housing  **IG**: Tai Chi (g) 15 min Qigong, followed by 45 min Yang-style Tai Chi, 2 classes per week (advised to attend) **Provider**: Tai Chi master **Duration**: 4 months  **Total contact**: 12h | **BCT clusters**: 4 Shaping knowledge  6 Comparison of behaviour 8 Repetition and substitution | No | 4 months | BMI  Musculoskeletal upper and lower body measures *Grip strength Arm curl test  Chair and stand  Partial curl- ups*  *Sit and reach  Timed 8-foot up and go test*  Mental and physical health *SF-36* | **16 weeks vs. baseline**: Anthropometrics: *BMI* d= -0.048 (**small**)***  Physical functioning: *Overall grip strength* d=0.312 (**small**)***, *arm curl* d=0.673 (**medium**)***, *chair stand test* d=0.717 (**medium**)***, *8-foot up and go test* d=-0.300 (**small; in favor CG**)***, *sit and reach* d=0.268 (**small**)**, *physical functioning* d= 0.326 (**small**)***, *role physical* d=0.037 (**small**)^#^, *physical health summary* d=0.192 (**small**)**  Generic health and well-being: *Bodily pain* d=0.047 (**small**)^#^, *general health* d=0.241 (**small**)** | Level of evidence: 2 Susceptibility to bias: Moderate |
| Melchior, M.A. (2013) [23] South Florida, United States | Pre-post design | **IG** n=682 **Age**: 76.4 years (8.7) **Ethnicity**: Hispanic **Female** 83% **Definition migrant**: Spanish speaking Hispanic  At least one chronic condition | Health management | **Setting**: Churches, nursing homes, community centers, residential community clubhouses, health clinics **IG**: *Tomando Control de su Salud* (g) Sessions by instructors including didactic lectures, role play, brainstorming, written assignments, modeling and goal-setting involving disease management skills, problem- solving techniques, critical thinking and how to appropriately use available resources **Provider**: Instructor **Duration**: 6 weeks **Total contact**: 15h | **BCT clusters**: 1 Goals and planning 4 Shaping knowledge 6 Comparison of behaviour 8 Repetition and substitution | Yes | 6 weeks | Time spent performing physical activity  *Likert response scale (0-5; >3h/week)*  Perceived social and role activities limitations for participants  *Likert response scale (0-4; not at all)* | **6 weeks vs. baseline**: Health behaviour: *Time spent stretching* d=0.56 (**medium**)^#^, *walking* d=0.41 (**small**)*, *aerobic activities* d=0.39 (**small**)**  Social functioning:  *Perceived social/ role activities limitations* d=0.05 (**small**)*** | Level of evidence: 2 Susceptibility to bias: Moderate |
| Palta, P. (2012) [24] Baltimore, United States | RCT | **IG** N = 12, **CG** N = 8 **Age**: IG 72.3 years (4.4) CG 73.7 years (5.8) **Ethnicity**: African American (all) **Female** 95% **Definition migrant**: Nm | Blood pressure management | **Setting**: Senior housing facility **IG**: Mindfulness-based stress reduction (g), ELDERSHINE Sessions on managing mental and physical health through mindfulness meditation and social and emotional skills development + Sharing experiences + Practicing and applying mindfulness skills to self-care and interpersonal relationships + Guided breath meditation + A healthy fruit-and-vegetable snack **CG**: mirroring topics in intervention group  + A healthy fruit-and-vegetable snack **Provider**: Interventionist trained in mindfulness-based stress reduction **Duration**: 2 months **Total contact**: 12h | **BCT clusters**: 3 Social support 4 Shaping knowledge  6 Comparison of behaviour  8 Repetition and substitution  11 Regulation  **BC theory/model**: NIH behaviour change consortium | No | 2 months | Blood pressure | **Intervention vs. control al 8 weeks** Anthropometrics: *SBP* d=0.43 (**small**)*, *DBP* d=0.092 (**small**)** | Level of evidence: 1 Susceptibility to bias: Low |
| Parisi, J.M. (2015) [25] Baltimore, United States | RCT | **IG** n=352, **CG** n=350 **Age**: 67.4 years (5.9) **Ethnicity**: Black/African American n= 646, White/European n= 35, Other n=21 **Female** 85% **Definition migrant**: Nm | Social functioning | **Setting**: Public elementary school **IG:** *Experience Corps* (i+g) Participants placed in a given public elementary school to a) support literacy development for children in kindergarten through third grade, b) support library functions under the guidance of a librarian, c) teach children how to solve problems and play, d) enhance school attendance (i)  + Participants were trained in team building problem-solving, planning, and socializing (g) **CG**: waitlist  **Provider**: Nm **Duration**: One school year **Total contact**: ~615h | **BCT clusters**: 3 Social support 4 Shaping knowledge  8 Repetition and substitution 10 Reward and threat | No | 4-8 months | Activity *LAQ Activity domain^[[11]](#footnote-11)^* | **Intervention vs. control at one year** *ITT analysis*:  Health behaviour: *Overall activity level**, *physical activities**, s*ocial activities*^#,^ *intellectual**, *passive* *activities*^#^, *creative* *activities*^#^  *CACE analysis* Health behaviour: *Overall activity level**, *physical activities**, s*ocial activities**, i*ntellectual**, *passive* *activities*^#^, *creative* *activities*^#^ | Level of evidence: 1 Susceptibility to bias: Low |
| Parker, S.J. (2011) [27] New York City, United States | Pre-post design | **IG** n=112 **Age**: 75.0 years (8.0) **Ethnicity**: African American n=37, Hispanic n=38, Non-Hispanic White n=37 **Female** 83% **Definition migrant**: Nm  Self-identified pain disorder | Physical activity | **Setting**: Senior centers **IG**: *Arthritis Foundation Self Help Program* (*ASHP*) (g) Education sessions regarding pain and its consequences  + Relaxation skills training, cognitive coping skills training, problem solving, and communication skills training + Weekly action plan embracing goal-setting, self-efficacy and support (i) + Arthritis education book (English and Spanish) For Spanish group: + Practicing exercise, 15-25 minutes + Two instructional CDs **Provider**: Instructor certified by the Arthritic Foundation **Duration**: 6 weeks **Total contact**: 12h | **BCT clusters**: 1 Goals and planning 4 Shaping knowledge  5 Natural consequences  6 Comparison of the behaviour  8 Repetition and substitution  10 Reward and threat  12 Antecedents | Yes | 6 weeks 18 weeks | Pain  Physical activity  Mood  *37-item, self-administered Arthritis Foundation* | **6 weeks vs. baseline** Physical functioning: *Stretching*  African American**, Hispanic**, Non-Hispanic White^#^ *Endurance*  African American**, Hispanic*, Non-Hispanic White*  *Relaxation* African American, Hispanic, Non-Hispanic White; all ***  Generic health and well-being: *Days of pain*  African American*, Hispanic^#^, Non-Hispanic White^#^  *Pain intensity* African American***, Hispanic*, Non-Hispanic White* *Mood*  African American^#^, Hispanic*, Non-Hispanic White**  **18 weeks vs. baseline**  Physical functioning: *Stretching*  African American***, Hispanic***, Non-Hispanic White** *Endurance*  African American, Hispanic, Non-Hispanic White; all ***  *Relaxation*  African American, Hispanic, Non-Hispanic White; all ***  Generic health and well-being:  *Days of pain* African American*, Hispanic^#^, Non-Hispanic White^#^  *Pain intensity* African American***, Hispanic*, Non-Hispanic White* | Level of evidence: 2 Susceptibility to bias: Low |
| Qi, B.B. (2001) [28]  United States | RCT | **IG** n=4, **CG** n=41 **Age**: 64.08 years (9.48) **Ethnicity**: Chinese **Female** 75.9% **Definition migrant**: Foreign-born Asian | Physical activity | **Setting**: Immigrant clinic associated with a Catholic church **IG**: *Self-efficacy-based Osteoporosis preventive educational* (*SEOPE*) (i+g) + One hour PowerPoint presentation (g) + Discussion by a nurse researcher^[[12]](#footnote-12)^ on osteoporosis prevention and medication use, benefits and barriers of exercise and taking supplements, and sharing experiences (g) + Supplemental handouts including individualized education booklet, Exercise and Screening for You screening tool, and safety tips for exercise initiation + Development of individualized goals and appropriate action strategies^[[13]](#footnote-13)^ (i) **CG**: Health promotion education PowerPoint on enhancing cerebrovascular health and brain, one hour **Provider**: Nurse researcher **Duration**: One hour **Total contact**: 1h | **BCT clusters**: 1 Goals and planning 3 Social support 5 Natural consequences 6 Comparison of behaviour  9 Comparison of outcomes  10 Reward and threat  12 Antecedents  **BC theory/model**: Theory of self-efficacy | Yes | 2 weeks | Physical activity *YPAS* | **Intervention vs. control at 2 weeks** Health behaviour: *Time spent in* *exercise* d=-0.16 (**small; in favor CG**)*, *exercise regularly**, *weight bearing exercise**** | Level of evidence: 1 Susceptibility to bias: Low |
| Rejeski, W.J. (2014) [29] Chicago area, United States | Parallel design RCT | **IG** n=88, **CG** n=90 **Age**: 70.66 years (9.44) **Ethnicity**: African American IG n=49 CG n=38 **Female** 50.56% **Definition migrant**: Nm | Physical activity | **Setting**: Home-based **IG**: Cognitive behavioural intervention (i+g); Discussions and session on peripheral artery disease (PAD), benefits of walking exercise, goal-setting, self-monitoring, and managing pain during exercise (g)  + Walking exercise around and indoor track at the exercise facility (g)  + Weekly developing walking goals for at least five days of the week (i) + Participants recorded actual exercise each day and severity of their pain/discomfort (i) **CG:** Health education information on management of hypertension, cancer screening, preventing falls, and vaccinations **Provider**: Facilitator **Duration**: 6 months  **Total contact**: 36h | **BCT clusters**: 1 Goals and planning 2 Feedback and monitoring  4 Shaping knowledge  6 Comparison of behaviour 8 Repetition and substitution  9 Comparison of behaviour  **BC theory/model**: Social cognitive theory | No | 6 months | Six-minute walk test  Social functioning *SPS* | **Intervention vs. control at** **6 months** Physical functioning: 6-minute walk*  Social functioning:  *Social functioning* d=0.63 (**medium**)**, *guidance* d=0.51 (**medium**)**, *reassurance of worth* d=0.64 (**medium**)**, *social integration* d=0.39 (**small**)^#^, *attachment* d=0.63 (**medium**)**, *nurturance* d=0.45 (**small**)*, *reliable* *alliance* d=0.26 (**small**)^#^ | Level of evidence: 1 Susceptibility to bias: High |
| Resnick, B. (2008) [30] New York City, United States | Cluster RCT | **IG** n=100, **CG** n=66 **Age**: IG 73.3 years (8.5)  CG 72.7 years (8.1)  **Ethnicity**: African American n=121, Latino n=33, Other n=12 **Female** 80.7% **Definition migrant**: Nm | Physical activity | **Setting**: Senior center **IG**: *Senior Exercise Self-efficacy Project* (*SESEP*)^[[14]](#footnote-14)^ (g) Physical activity and efficacy-enhancing intervention  + Learn about the benefit of exercise and PA + Discuss unpleasant sensations associated with exercise and how to eliminate this + Goal development, with verbal encouragement (i) + Provision of loose-leaf binder with recommended exercise program and a picture and brief description of how to perform each exercise **CG**: Nutrition education **Provider**: Lay exercise trainers **Duration**: 3 months  **Total contact**: 30h | **BCT clusters**: 1 Goals and planning 4 Shaping knowledge  6 Comparison of behaviour 8 Repetition and substitution  9 Comparison of behaviour  15 Self- belief  **BC theory/model**: Theory of self-efficacy | Yes | 3 months | Exercise Overall activity *YPAS*  Health related quality of life *SF-12*  Depressive symptoms *GDS*  Mobility *Tinitti Scale*  Functional decline *Chair rise*  Pain  *Rating 0-10* | **Intervention vs. control at 12 weeks** Health behaviour: *Exercise* d=0.23 (**small**)*, *overall physical activity* d=0.14 (**small**)^#^  Physical functioning: *Chair rise* d=-0.22 (**small; in favor CG**)*, *mobility* d=0.29 (**small**)^#^  Mental health and functioning: *Depressive symptoms* d=-0.40 (**small**)*  Generic health and well-being: *Physical health related QoL* d=-0.22 (**small; in favor CG**)^#^, *mental health related QoL* d=0.56 (**medium**)^#^, *pain* d=0.33 (**small**)^#^ | Level of evidence: 1 Susceptibility to bias: Low |
| Sin, M.K. (2005) [31] Seattle, United States | Pre-post design | **IG** n=13 **Age**: 77 years (5.9) **Ethnicity**: Korean (all) **Female** 61.5% **Definition migrant**: Nm | Physical activity | **Setting**: Multi-purpose dining room of the senior house where the participants lived **IG**: Exercise program^[[15]](#footnote-15)^ (g); balance, flexibility, strength training and aerobic capacity **Provider**: Korean-American instructor **Duration**: 3 months  **Total contact**: 30h | **BCT clusters**: 4 Shaping knowledge  6 Comparison of behaviour 8 Repetition and substitution | Yes | 3 months | Blood pressure  Upper extremity muscle strength  *Arm curl test*  Agility and balance  *8-foot-Up-and-Go test* | **12 weeks vs. baseline** Anthropometrics: *SBP* d=-0.51 (**medium**)*, DBP d=0.16 (**small**)^#^  Physical functioning:  *Arm curls* d=1.12 (**large**)**, *8-foot-Up-and-Go* d=-0.28 (**small; in favor CG**)* | Level of evidence: 2 Susceptibility to bias: Moderate |
| Sun, W.Y. (1996) [32] La Crosse County, United States | RCT | **IG** n=10, **CG** n=10 **Age**: 60-64 years n=7 65-69 years n=9 70-74 years n=4 **Ethnicity**: Hmong American (all) **Female** 65% **Definition migrant**: Nm | Physical activity | **Setting**: Nm **IG**: Tai Chi Chuan (g)  + Mini-lecture on simple human physiological systems and common diseases in older adults, as well as information on emotional and mental health and stress management (g) **CG**: were asked to continue routine physical activity **Provider**: Nm **Duration**: 3 months  **Total contact**: 26h | **BCT clusters**: 4 Shaping knowledge  6 Comparison of behaviour 8 Repetition and substitution  11 Regulation | Yes | 3 months | Heart rate Resting BP  Flexibility various joints Self perceived stress Stress level according to skin temperature  *Tai Chi Chuan Program Inventor^[[16]](#footnote-16)^* | **Intervention vs. control at 12 weeks** Anthropometrics:  *Resting SBP* d=1.31 (**large**)*, *resting DBP* d=1.94 (**large**)**  Physical functioning:  *Flexibility of trunk* d=0.86 (**large**) ^#^, *shoulder* d=1.46 (**large**)**, *knee* d=1.46 (**large**)**  Generic health and well-being:  *Self-perceived stress scores* d=3.19 (**large**)**, *stress level according to body temperature* d=1.96 (**large**)** | Level of evidence: 1 Susceptibility to bias: Low |
| Taylor-Piliae, R.E. (2006) [33, 34] San Francisco Bay Area, United States | Quasi-experiment | **IG** n=39 **Age**: 66 years (8.3) **Ethnicity**: Chinese (all) **Female** 69% **Definition migrant**: Ethnic Chinese  At least one cardiovascular disease risk factor^[[17]](#footnote-17)^ | Physical activity | **Setting**: Community center  **IG**: Tai Chi exercise intervention (g) + On completion, each participant received a CD-Rom of the Tai Chi Master performing the form taught **Provider**: Tai Chi master **Duration**: 12 weeks **Total contact**: 36h | **BCT clusters**: 4 Shaping knowledge  6 Comparison of behaviour 8 Repetition and substitution  10 Reward and threat | Yes | 6 weeks 12 weeks | Blood pressure  Aerobic endurance *Two-minute step-in-place*  Social support  *MSPSS*^[[18]](#footnote-18)^  Perceived stress  PSS^18^  Mood  POMS^18^ | **6 weeks vs. baseline**: Anthropometrics: *SBP***, *DBP***  Physical functioning: *2-min step count***  **12 weeks vs. baseline**: Anthropometrics: *SBP* d=1.73 (**large**)**, *DBP* d=1.60 (**large**)**  Physical functioning:  *2 min step count* d=1.60 (**large**)**  Social functioning: *Overall Social Support* d=0.74 (**medium**)**, *family* d=0.60 (**medium**)*, *friends* d=0.35 (**small**)*, *significant other* d=0.81 (**medium**)*  Generic health and well-being: *Perceived stress* d=0.77 (**large**)**, *total mood disturbance* d=0.74 (**medium**)**, *tension–anxiety* d=0.84 (**large**)**, *confusion–bewilderment* d=0.67 (**medium**)*, *depression–dejection* d=0.46 (**medium**)^#^, *anger–hostility* d=0.55 (**medium**)^#^, *vigor–activity* d=0.55 (**medium**)^#^, *fatigue–inertia* d=0.46 (**medium**)^#^ | Level of evidence: 2 Susceptibility to bias: Moderate |
| Wilcox, S. (2006) [35] United States | Quasi-experiment | **IG** *AC* n=384 *ALED* n=454 **Age**: 68.4 years (9.4) **Ethnicity**: Asian n=17, Black/African American n=250, Native Hawaiian/Pacific Islander n=2, White n=536, Reporting 2 groups n=10, Other n=23 **Female** 80.6% **Definition migrant**: Nm | Physical activity | **Setting**: *AC*: home-based *ALED*: Community-based **IG**:  *Active Choices* (*AC*) (i): One face-to-face meeting to develop PA plan and goals and discus interest, motivation, perceived benefits and barriers to PA, and exercise safety + Provision of exercise log, pedometer + Provision local resource guide of PA programs + Counseling tailored to participant’s readiness to change to promote PA. Discussing potential changes in health and exercise related injuries, current PA level, modifying goals (if needed), biweekly telephone calls for first 2 months, monthly last 4 month + Tip sheets based on call content  *Active Living Every Day* (*ALED*) (g); Sessions on learning behaviour change principles + Sharing success and challenges and providing support to another  **Provider**: Nm **Duration**: *AC*: 6 weeks *ALED*: 20 weeks **Total contact**: Nm | **BCT clusters**:  *AC* 1 Goals and planning  2 Feedback and monitoring 3 Social support 9 Comparison of outcome 12 Antecedents   *ALED* 3 Social support 4 Shaping knowledge  6 Comparison of behaviour   **BC theory/model**: *AC* and *ALED*: Social cognitive theory | No | *AC*  6 weeks  *ALED*  20 weeks | BMI   Physical activity  *CAMPS*  Depressive symptoms  *CES-D*  Perceived stress *Perceived stress scale* | **AC 6 weeks vs. baseline**  Anthropometrics: *BMI* d= -0.03 (**small**)***   Health behaviour: *MVPA* d=0.74*** (**medium**), *all PA* d= 0.41 (**small**)***  Mental health and functioning: *Depressive symptoms* d=-0.03 (**small**)^#^  Generic health and well-being: *Perceived stress* d=0.00 (**small**)^#^   **ALED 20 weeks vs. baseline**  Anthropometrics: *BMI* d= -0.05 (**small**)***   Health behaviour: *MVPA* d=0.56 (**medium**)**, *all PA* d= 0.60 (**medium**)**  Mental health and functioning: *Depressive symptoms* d=-0.12 (**small**)*  Generic health and well-being: *Perceived stress* d=-0.21 (**small**)* | Level of evidence: 2 Susceptibility to bias: Low |
| Wilcox, S. (2008) [36] United States | Quasi-experiment | **IG** *AC* Year 1 n=384 Year 2 n=1136 Year 3 n=982  *ALED* Year 1 n=454 Year 2 n=1433 Year 3 n=1501 **Age**: *AC*  Year 1: 65.9 years (9.8) Year 2: 66.0 years (9.6) Year 3: 65.6 years (10.0) *ALED* Year 1: 70.6 years (8.6) Year 2: 70.9 years (9.4) Year 3: 70.4 years (9.6) **Ethnicity**: *AC* American Indian/Alaskan Native Year 1: n=0 Year 2: n=1 Year 3: n=2  Asian Year 1: n=13 Year 2: n=55 Year 3: n=60 Black/African American Year 1 n=123 Year 2 n=499 Year 3 n=413 Latino  Year 1: n=36 Year 2: n=159 Year 3: n=71 Native Hawaiian/ Pacific Islander Year 1: n=0 Year 2: n=6 Year 3: n=7 Non-Hispanic White Year 1 n=205 Year 2 n=389 Year 3 n=421  Reporting 2 groups Year 1: n=4 Year 2: n=12 Year 3: n=3 Other  Year 1: n=3 Year 2: n=15 Year 3: n=5 *ALED*  American Indian/Alaskan Native Year 1 n=0 Year 2 n=11 Year 3 n=6  Asian Year 1 n=3 Year 2 n=10 Year 3 n=17  Black/African American  Year 1 n=124 Year 2 n=447 Year 3 n=463  Latino  Year 1 n=13 Year 2 n=39 Year 3 n=39  Native Hawaiian/ Pacific Islander Year 1 n=0 Year 2 n=0 Year 3 n=2 Non-Hispanic White Year 1 n=312 Year 2 n=909 Year 3 n=958 Reporting 2 groups Year 1 n=1 Year 2 n=9 Year 3 n=14 Other Year 1 n=1 Year 2 n=9 Year 3 n=5 **Female** AC Year 1 n= 78.3% Year 2 n= 80.1 % Year 3 n= 80.6% ALED Year 1 n= 82.6% Year 2 n= 82.5% Year 3 n=84.8% **Definition migrant**: Nm | Physical activity | **Setting**: *AC*: home-based *ALED*: Community-based **IG**: *Active Choices* (*AC*) (i): One face-to-face meeting to develop PA plan and goals and discus interest, motivation, perceived benefits and barriers to PA, and exercise safety + Provision of exercise log, pedometer + Provision local resource guide of PA programs + Counseling tailored to participant’s readiness to change to promote PA. Discussing potential changes in health and exercise related injuries, current PA level, modifying goals (if needed), biweekly telephone calls for first 2 months, monthly last 4 month + Tip sheets based on call content  *Active Living Every Day* (*ALED*)^[[19]](#footnote-19)^ (g): Sessions on learning behaviour change principles + Sharing success and challenges and providing support to another  **Provider**: Nm **Duration**: *AC*: 6 weeks *ALED*: 12 weeks **Total contact**: Nm | **BCT clusters**:  *AC* 1 Goals and planning  2 Feedback and monitoring 3 Social support 9 Comparison of outcome 12 Antecedents   *ALED* 3 Social support 4 Shaping knowledge  6 Comparison of behaviour  **BC theory/model**: *AC* and *ALED*: Social cognitive theory | No | *AC*  6 weeks  *ALED*  20 weeks | BMI   Physical activity *CAMPS*   Depressive symptoms *CES-D*   Perceived stress *Perceived stress scale* | AC  Anthropometrics:  BMI (all **small**)* year 1 d= -0.03, year 3 d=-0.04, year 4 d=-0.03  Health behaviour: *MVPA* (all **small**)* year 1 d=0.43, year 3 d=0.36, year 4 d= 0.43  *All PA* (all **small**)* year 1 d=0.41, year 3 d=0.32, year 4 d=0.37   Mental health and functioning *Depressive symptoms* (all **small**)^#^  year 1 d=-0.03, year 3 d= -0.03, year 4 d= 0.02  Generic health and well-being: *Perceived stress* (all **small**)^#^   year 1 d=0.00, year 3 d= -0.03, year 4 d=0.03  ALED Anthropometrics: BMI (all **small**)*  year 1 d= -0.05, year 3 d=-0.02, year 4 d=-0.03/-0.04  Bheaviour:  *MVPA* year 1 d=0.56 (**medium**)* year 3 d=0.46 (**medium**)* year 4 d= 0.42 (**small**)* *All PA* year 1 d=0.60 (**medium**)* year 3 d= 0.39 (**small**)* year 4 d= 0.46 (**medium**)*   Mental health and functioning:  *Depressive symptoms* (all **small**)^#^ year 1 d=-0.12, year 3 d=-0.09, year 4 d=-0.07  Generic health and well-being: *Perceived stress* (all **small**)* year 1 d=-0.21, year 3 d=-0.15, year 4 d=-0.10 | Level of evidence: 2 Susceptibility to bias: Low |
| Wolf, R.L. (2009) [37] New York City, United States | RCT | **IG** n=240, **CG** n=239 **Age**: ≥45 years  **Ethnicity**: African  **Female** Nm  **Definition migrant**: Self-identify as being of African descent (Black, African American, Caribbean, African) | Diet | **Setting**: Home-based  **IG:**: Tailored Telephone Education (TTE) (i); Recommendations on fruit and vegetable intake embracing amounts, serving size, the importance of eating a colorful variety of FVs, and potential health benefits linked to FV intake  + Tailored recommendations and tips based on awareness of health benefits linked to FV intake, bolster motivation by tailoring participant’s values, identification which FV most consumed, followed by positive reinforcement, 2 monthly + Mailed NCI’s print brochure, “Men Eat 9 A Day” + Brief follow-up call of average 5 minutes; asking for possible barriers, attempted to identify practical goals for increasing FV at meals and snacks, providing social and emotional support and, finally, attempting to elicit a verbal commitment to increase FV intake based on the goals set  **CG**: Tailored Telephone Education on Prostate cancer education  + Mailed NCI’s print brochure, “Men Eat 9 A Day”  **Provider**: Health educator **Duration**: Nm **Total contact:** Nm | **BCT clusters**: 1 Goals and planning  3 Social support 5 Natural consequences  10 Reward and threat **BC theory/model**: Transtheoretical model | No | 8 months | Fruit and vegetable intake *Self-reported behaviour* | **Intervention vs. control at 8 months** Health behaviour: *Fruit and vegetable* d=0.39 (**small**)***, *fruit* d=0.41 (**small**)***, *vegetable* d=0.25 (**small**)** | Level of evidence: 1 Susceptibility to bias: Low |
| Yan, T. (2009) [38]  Los Angles, United States | Quasi- experiment | **IG** n=151, **CG** n=57 **Age**: 72.88 years (8.83) **Ethnicity**: Black n=45, Hispanic n=105, White n=47, Other n=4, Missing n=7 **Female** 82.21% **Definition migrant**: Nm  Sedentary or underactive^[[20]](#footnote-20)^ | Physical activity | **Setting**: Senior center  **IG**: *Active Start* (g); the first 4 weeks *Active Living Every Day* (*ALED)* only, followed by 16 weeks *ALED* and *ExerStart* combined program (each 45 minutes), last 4 weeks *ExerStart* only. *(Active Living Every Day)*; Goal-setting, identify barriers, and establish social support systems regarding PA, weekly one hour + A number of behavior change strategies designed to change behavior in small steps were introduced in the first 4 weeks by assessing motivation, identifying readiness, goal setting, education, starting with small goals, and identifying barriers  *+ (ExerStart)*; exercise + Handout at the end of each class that included a safe exercise they could practice and complete at home.  **CG**: Waitlist **Provider**: Lay leaders **Duration**: 6 months **Total contact:** 36h | **BCT clusters**: 1 Goals and planning  3 Social support 4 Shaping knowledge  6 Comparison of the behaviour  8 Repetition and substitution **BC theory/model**: Transtheoretical model | Yes | 6 months | Lower and upper body strength  *Senior fitness test* | **24 weeks vs. baseline** Physical functioning:  *Chair stand* (all **large**)***  White d=0.77  Black d=0.92  Hispanic d= 1.11  All participants d=0.98  *Arm curl* (all **large**)*** White d=1.01  Black d=1.10  Hispanic d=1.25  All participants d= 1.21  *2-min test*  White d= 0.65 (**medium**)*** Black d=0.97 (**large**)** Hispanic d=0.76 (**large**)*** All participants d=0.76 (**large**)*** *Chair sit and reach* (all **small**) White d=0.32^#^  Black d=0.12^#^  Hispanic d=0.42*  All participants d=0.34***  *Back scratch* White d=0.32 (**small**)^#^ Black d=0.47 (**medium**)* Hispanic d=0.40 (**small**)* All participants d= 0.40 (**small**)*** *8 foot up and go*  White d=-0.40 (**small; in favor CG**)*** Black d=-0.55 (**small; in favor CG**)*** Hispanic d=-0.55 (**medium**)*** All participants d=-0.55 (**small; in favor CG**)*** | Level of evidence: 2 Susceptibility to bias: Low |
| Yan, T. (2009) [39] California, United States | Pre-post design | **IG** n=518 **Age**: 80.1 years (7.25) **Ethnicity**: African American n=115, Hispanic n=210, White n=132, Other n=42, Unknown n=19 **Female** 83.78% **Definition migrant**: Nm  Impairment in ≥2 activities of daily living (ADL) or one ADL deficiency and cognitive impairment. | Physical activity | **Setting**: Home-based **IG**: *Healthy Moves for Aging Well* program (i) Combination of face-to-face, on-site instruction and face-to-face phone coaching or telephone coaching only  + Behaviour- change counseling involving participant’s motivation to change behaviour by monitoring participant’s progress with the exercises covering goal-setting, encouraging of participant to come up with solutions to challenges, offered social support and recommendations when appropriate, and built confidence in the participates’ ability to make positive changes, weekly first 2 months, biweekly third month  **Provider:** Volunteer coaches  **Duration**: 3 months **Total contact:** Nm | **BCT clusters**: 1 Goals and planning 2 Feedback and monitoring  3 Social support 4 Shaping knowledge 15 Self-belief | Yes | 3 months | Depression  Pain | **3 months vs. baseline** Mental health and functioning: *Depression*^#^  Generic health and well-being: *Pain level** | Level of evidence: 2 Susceptibility to bias: Low |
| Yeom, H. (2013) [40] United States | Quasi-experiment | **IG** n=33, **CG** n=31 **Age**: 71 years (7.44) **Ethnicity**: Korean American **Female** 23.4% **Definition migrant**: Nm  Sedentary | Physical activity  Social activity | **Setting**: Local Salvation Army Church **IG**: *Motivational Physical Activity Intervention* (*MPAI*) (i+g);  Physical activity training + Social support operationalized through group process, goal setting, and interaction (g) + Empowering education focusing on the creation of social contextual resources  + Motivational support for enhancing motivational appraisal and skills to initiate and sustain regular physical activity (i)  **CG**: biweekly newsletters **Provider**: (Bilingual) researcher **Duration**: 12 weeks **Total contact**: 24h | **BCT clusters**: 1 Goals and planning  2 Feedback and monitoring 3 Social support 4 Shaping knowledge  5 Natural consequences 6 Comparison of the behaviour  8 Repetition and substitution **BC theory/model**: Wellness Motivation Theory | Yes | 13 weeks | Walking endurance *6-minute walk*  Balance Gait velocity Flexibility *SPPB*  Social support *SSES* | **Intervention vs. control at 12 weeks** Physical functioning: *6 minute walk* d=0.49 (**medium**)***,   SPPB total d= 0.55 (**medium**)***, SPPB – balance d=0.23 (**small**)^#^, SPPB – gait velocity d=0.32 (**small**)^#^, SPPB – flexibility d=0.64 (**medium**)***  Social functioning: *Social support from family* d=0.43 (**small**)***, *friends* d=0.81 (**large**)*** | Level of evidence: 2 Susceptibility to bias: Low |

*p≤0.05, **p≤0.01, ***p≤0.001,^#^not significant

ADL: Activities of Daily Living, ADL: Activities of Daily Living, ASHP: Arthritis Foundation Self Help Program, BC: behavioural change, BMI: Body Mass Index, CACE: Complier Average Causal Effect, CDC: Centers for Disease Control and Prevention, CES-D: Center for Epidemiologic Studies Depression, CHAMPS: Community Healthy Activities Model Program for Seniors, CWT: Complex Walking Task, DBP: Diastolic Blood Pressure, F&V: Fruit and Vegetable, (g): group-based GDS-15: Geriatric Depression Scale, Geriatric Depression Scale: GDS,HBP: High Blood Pressure, HbA1c = Hemoglobin A1c, (i): individual, IPAQ: International Physical Activity Questionnaire, ITT: Intention-to-treat, LAQ: Lifestyle Activity Questionnaire, LSI-Z: Life Satisfaction Index-Z,LSNS: Lubben Social Network Scale, LTEQ: Leisure Time Exercise Questionnaire, MAPA-f: Meaningful Activity Participation Assessment-Frequency, MMSE: Mini-Mental State Examination, MNA: Mini Nutrition Assessment, MSPSS: Multidimensional Scale of Perceived Social Support, MVPA: Moderate to Vigorous intensity Physical Activity, Na: not applicable, NCI: National Cancer Institute; SFT: Senior Fitness Test, NHC: Nurse Health Coach, NHNES: National Health and Nutrition Examination Survey, NIH: National Institutes of Health, Nm: not mentioned, NPA: Nutrition and Physical Activity, PA: Physical Activity, PAR: Physical Activity Recall, PCP: Primary Care Provider,PHQ-9: Patient Health Questionnaire- 9,POMS: Profile of Mood States,PPS-10: 10-item Perceived Stress Scale, PSS: Perceived Stress Scale, QoL: Quality of Life, RAPA: Rapid Assessment of Physical Activity, RCT: Randomized Controlled Trial, REAP: Rapid Eating and Activity Assessment for Patients, SBP: Systolic Blood Pressure,SF-12: Short Form-12,SF-36v2: Version 2 of the 36-Item Short-Form Health Survey, SPPB: Short Physical Performance Battery, SPPB: Short Physical Performance Battery, SPS: Social Provision Scale, SSES: Social Support and Exercise Survey, TMT: Trail Making Test, UCLA: University of California, UN: United Nations, WHODAS-II: 32-item World Health Organization Disability Assessment Schedule 2.0,YMCA: Young Men’s Christian Association, YPAS: Yale Physical Activity Scale

1. Agurs-Collins TD, Kumanyika SK, Ten Have TR, Adams-Campbell LL: **A randomized controlled trial of weight reduction and exercise for diabetes management in older African-American subjects**. *Diabetes Care* 1997, **20**(10):1503-1511.

2. Batik O, Phelan EA, Walwick JA, Wang G, LoGerfo JP: **Translating a community-based motivational support program to increase physical activity among older adults with diabetes at community clinics: a pilot study of Physical Activity for a Lifetime of Success (PALS)**. *Prevention Chronic Disease* 2008, **5**(1):A18.

3. Beissner K, Parker SJ, Henderson Jr CR, Pal A, Iannone L, Reid MC: **A cognitive-behavioral plus exercise intervention for older adults with chronic back pain: race/ethnicity effect?** *Journal of Aging and Physical Activity* 2012, **20**(2):246-265.

4. Clark F, Jackson J, Carlson M, Chou CP, Cherry BJ, Jordan-Marsh M, Knight BG, Mandel D, Blanchard J, Granger DA *et al*: **Effectiveness of a lifestyle intervention in promoting the well-being of independently living older people: Results of the Well Elderly 2 Randomised Controlled Trial**. *Journal of Epidemiology and Community Health* 2012, **66**(9):782-790.

5. Juang C, Knight BG, Carlson M, Schepens Niemiec SL, Vigen C, Clark F: **Understanding the Mechanisms of Change in a Lifestyle Intervention for Older Adults**. *Gerontologist* 2018, **58**(2):353-361.

6. Collins CC, Benedict J: **Evaluation of a community-based health promotion program for the elderly: lessons from Seniors CAN**. *American Journal of Health Promotion* 2006, **21**(1):45-48.

7. Dogra S, Shah S, Patel M, Tamim H: **Effectiveness of a Tai Chi intervention for improving functional fitness and general health among ethnically diverse older adults with self-reported arthritis living in low-income neighborhoods: a cohort study**. *Journal of Geriatric Physical Therapy* 2015, **38**(2):71-77.

8. Emery-Tiburcio EE, Mack L, Lattie EG, Lusarreta M, Marquine M, Vail M, Golden R: **Managing Depression among Diverse Older Adults in Primary Care: The BRIGHTEN Program**. *Clinical Gerontologist* 2017, **40**(2):88-96.

9. Fernandez S, Scales KL, Pineiro JM, Schoenthaler AM, Ogedegbe G: **A senior center-based pilot trial of the effect of lifestyle intervention on blood pressure in minority elderly people with hypertension**. *Journal of the American Geriatrics Society* 2008, **56**(10):1860-1866.

10. Fried LP, Carlson MC, Freedman M, Frick KD, Glass TA, Hill J, McGill S, Rebok GW, Seeman T, Tielsch J *et al*: **A social model for health promotion for an aging population: initial evidence on the Experience Corps model**. *Journal of urban health : bulletin of the New York Academy of Medicine* 2004, **81**(1):64-78.

11. Geller KS, Mendoza ID, Timbobolan J, Montjoy HL, Nigg CR: **The Decisional Balance Sheet to Promote Healthy Behavior Among Ethnically Diverse Older Adults**. *Public Health Nurs* 2012, **29**(3):241-246.

12. Goldfinger JZ, Arniella G, Wylie-Rosett J, Horowitz CR: **Project HEAL: Peer education leads to weight loss in harlem**. *Journal of Health Care foor the Poor and Underserved* 2008, **19**(1):180-192.

13. Hau C, Reid KF, Wong KF, Chin RJ, Botto TJ, Eliasziw M, Bermudez OI, Fielding RA: **Collaborative evaluation of the healthy habits program: An effective community intervention to improve mobility and cognition of Chinese older adults living in the U.S**. *Journal Nutrition Health and Aging* 2016, **20**(4):391-397.

14. Holland SK, Greenberg J, Tidwell L, Malone J, Mullan J, Newcomer R: **Community-based health coaching, exercise, and health service utilization**. *Journal of Aging and Health* 2005, **17**(6):697-716.

15. Hooker SP, Harmon B, Burroughs EL, Rheaume CE, Wilcox S: **Exploring the feasibility of a physical activity intervention for midlife African American men**. *Health Education Research* 2011, **26**(4):732-738.

16. Jih J, Le G, Woo K, Tsoh JY, Stewart S, Gildengorin G, Burke A, Wong C, Chan E, Fung LC *et al*: **Educational Interventions to Promote Healthy Nutrition and Physical Activity Among Older Chinese Americans: A Cluster-Randomized Trial**. *American Journal of Public Health* 2016, **106**(6):1092-1098.

17. Keller CS, Cantue A: **Camina por Salud: walking in Mexican-American women**. *Applied Nursing Research* 2008, **21**(2):110-113.

18. Kim BH, Glanz K: **Text messaging to motivate walking in older african americans: A randomized controlled trial**. *American Journal of Preventive Medicine* 2013, **44**(1):71-75.

19. Kim KB, Han HR, Huh B, Nguyen T, Lee H, Kim MT: **The effect of a community-based self-help multimodal behavioral intervention in Korean American seniors with high blood pressure**. *American Journal of Hypertension* 2014, **27**(9):1199-1208.

20. Lu Y, Dipierro M, Chen L, Chin R, Fava M, Yeung A: **The evaluation of a culturally appropriate, community-based lifestyle intervention program for elderly Chinese immigrants with chronic diseases: a pilot study**. *Journal of public health (Oxford, England)* 2014, **36**(1):149-155.

21. Manson J, Ritvo P, Ardern C, Weir P, Baker J, Jamnik V, Tamim H: **Tai Chi's Effects on Health-Related Fitness of Low-Income Older Adults**. *Canadian Journal on Aging* 2013, **32**(3):270-277.

22. Manson J, Rotondi M, Jamnik V, Ardern C, Tamim H: **Effect of tai chi on musculoskeletal health-related fitness and self-reported physical health changes in low income, multiple ethnicity mid to older adults**. *BMC Geriatrics* 2013, **13**:114.

23. Melchior MA, Seff LR, Bastida E, Albatineh AN, Page TF, Palmer RC: **Intermediate outcomes of a chronic disease self-management program for Spanish-speaking older adults in South Florida, 2008-2010**. *Prevention Chronic Disease* 2013, **10**:E146.

24. Palta P, Page G, Piferi RL, Gill JM, Hayat MJ, Connolly AB, Szanton SL: **Evaluation of a mindfulness-based intervention program to decrease blood pressure in low-income African-American older adults**. *Journal of urban health : bulletin of the New York Academy of Medicine* 2012, **89**(2):308-316.

25. Parisi JM, Kuo J, Rebok GW, Xue Q-L, Fried LP, Gruenewald TL, Huang J, Seeman TE, Roth DL, Tanner EK *et al*: **Increases in lifestyle activities as a result of experience Corps® participation**. *Journal of urban health : bulletin of the New York Academy of Medicine* 2015, **92**(1):55-66.

26. Parisi JM, Rebok GW, Seeman TE, Tanner EK, Tan EJ, Fried LP, Xue Q-L, Frick KD, Carlson MC: **Lifestyle Activities in Sociodemographically at-risk Urban, Older Adults Prior to Participation in the Baltimore Experience Corps(®) Trial**. *Act Adapt Aging* 2012, **36**(3):242-260.

27. Parker SJ, Vasquez R, Chen EK, Henderson CR, Jr., Pillemer K, Robbins L, Reid MC: **A comparison of the arthritis foundation self-help program across three race/ethnicity groups**. *Ethnicity and Disease* 2011, **21**(4):444-450.

28. Qi BB, Resnick B, Smeltzer SC, Bausell B: **Self-efficacy program to prevent osteoporosis among Chinese immigrants: a randomized controlled trial**. *Nursing research* 2011, **60**(6):393-404.

29. Rejeski WJ, Spring B, Domanchuk K, Tao H, Tian L, Zhao L, McDermott MM: **A group-mediated, home-based physical activity intervention for patients with peripheral artery disease: effects on social and psychological function**. *J Transl Med* 2014, **12**:29-29.

30. Resnick B, Luisi D, Vogel A: **Testing the Senior Exercise Self-efficacy Project (SESEP) for use with Urban dwelling minority older adults**. *Public Health Nurs* 2008, **25**(3):221-234.

31. Sin MK, Belza B, LoGerfo J, Cunningham S: **Evaluation of a community-based exercise program for elderly Korean immigrants**. *Public Health Nurs* 2005, **22**(5):407-413.

32. Sun WY, Dosch M, Gilmore GD, Pemberton W, Scarseth T: **Effects of a Tai Chi Chuan program on Hmong American older adults**. *Educational Gerontology* 1996, **22**(2):161-167.

33. Taylor-Piliae RE, Haskell WL, Sivarajan Froelicher E: **Hemodynamic responses to a community-based Tai Chi exercise intervention in ethnic Chinese adults with cardiovascular disease risk factors**. *European Journal of Cardiovascular Nursing: journal of the Working Group on Cardiovascular Nursing of the European Society of Cardiology* 2006, **5**(2):165-174.

34. Taylor-Piliae RE, Haskell WL, Waters CM, Froelicher ES: **Change in perceived psychosocial status following a 12-week Tai Chi exercise programme**. *Journal of Advanced Nursing* 2006, **54**(3):313-329.

35. Wilcox S, Dowda M, Griffin SF, Rheaume C, Ory MG, Leviton L, King AC, Dunn A, Buchner DM, Bazzarre T *et al*: **Results of the first year of active for life: translation of 2 evidence-based physical activity programs for older adults into community settings**. *American Journal of Public Health* 2006, **96**(7):1201-1209.

36. Wilcox S, Dowda M, Leviton LC, Bartlett-Prescott J, Bazzarre T, Campbell-Voytal K, Carpenter RA, Castro CM, Dowdy D, Dunn AL *et al*: **Active for Life. Final Results from the Translation of Two Physical Activity Programs**. *American Journal of Preventive Medicine* 2008, **35**(4):340-351.

37. Wolf RL, Lepore SJ, Vandergrift JL, Basch CE, Yaroch AL: **Tailored telephone education to promote awareness and adoption of fruit and vegetable recommendations among urban and mostly immigrant black men: a randomized controlled trial**. *Preventive Medicine* 2009, **48**(1):32-38.

38. Yan T, Wilber KH, Aguirre R, Trejo L: **Do sedentary older adults benefit from community-based exercise? results from the active start program**. *Gerontologist* 2009, **49**(6):847-855.

39. Yan T, Wilber KH, Wieckowski J, Simmons WJ: **Results from the healthy moves for aging well program: Changes of the health outcomes**. *Home Health Care Services Quarterly* 2009, **28**(2-3):100-111.

40. Yeom HA, Fleury J: **A Motivational Physical Activity Intervention for Improving Mobility in Older Korean Americans**. *Western Journal of Nursing Research* 2014, **36**(6):713-731.

1. Based on Diabetic Exchange Lists for Meal Planning [↑](#footnote-ref-1)
2. Volunteer training materials were stream lined, materials’ literacy level were reduced [↑](#footnote-ref-2)
3. Cornoni-Huntley J, Ostfeld AM, Taylor JO, Wallace RB, Blazer D, Berkman LF, et al. Established populations for epidemiologic studies of the elderly: study design and methodology. Aging (Milan, Italy). 1993 Feb;5(1):27-37 [↑](#footnote-ref-3)
4. In interview format to minimize sensory, functional, and literacy barriers [↑](#footnote-ref-4)
5. SBP≥140mmHg or DBP≥90 mmHg; for those with diabetes mellitus or kidney disease, SBP≥30mmHg or DBP≥80 mmHg [↑](#footnote-ref-5)
6. Overweight (BMI 25–29.9 kg/m^2^) or obese (BMI ≥30kg/m^2^) [↑](#footnote-ref-6)
7. Modified version to minimize potential problems related to language barriers [↑](#footnote-ref-7)
8. Participants in both treatment and control groups continued to be cared for by their primary care physician and received coverage for health services under their regular HMO benefit contracts [↑](#footnote-ref-8)
9. Basic recommendations regarding maintaining a healthy diet and an optimal aerobic physical activity level, adopted from the National 2005 Dietary Guidelines of America and the 2008 Physical Activity Guidelines [↑](#footnote-ref-9)
10. Categorized by the Physical Activity Recall [↑](#footnote-ref-10)
11. Based on an extensive review of the literature and previous work [26. Parisi JM, Rebok GW, Seeman TE, Tanner EK, Tan EJ, Fried LP, Xue Q-L, Frick KD, Carlson MC: **Lifestyle Activities in Sociodemographically at-risk Urban, Older Adults Prior to Participation in the Baltimore Experience Corps(®) Trial**. *Act Adapt Aging* 2012, **36**(3):242-260. of the authors; ***Intellectual***: discussing local/national issues, reading a book, reading a newspaper, balancing a checkbook, using a computer, crossword puzzles, taking courses/ classes ***Social***: attending church/religious service, visiting, clubs/organizations, playing cards/ games, going to movies, going to plays/concerts ***Physical***: shopping, gardening, hunting/fishing/camping ***Creative***: preparing food, sewing/ mending /fixing things, singing/ playing instrument, drawing/painting, looking at art ***Passive***: watching TV, listening to music/ radio [↑](#footnote-ref-11)
12. Based on bone mineral density, risk factors, and diet and activity preferences [↑](#footnote-ref-12)
13. Based on the National Institutes of Health’s (2006) booklet Bone Health and Osteoporosis: A guide for Asian Women Aged 50 and Older; Families or relatives were encouraged to join [↑](#footnote-ref-13)
14. Weekly reminders for class times were posted in senior centers [↑](#footnote-ref-14)
15. Telephone reminders 5 minutes before exercise class were given to people who did not attend the class; Participants had to be able to speak and write Korean [↑](#footnote-ref-15)
16. Translated into Hmong language [↑](#footnote-ref-16)
17. Current smoker, high blood pressure, high cholesterol, diabetes [↑](#footnote-ref-17)
18. Translation and independent back-translation of the tool into Chinese by bilingual and/or bicultural persons with translation expertise and graduate level education [↑](#footnote-ref-18)
19. Year 4 program shortened to 12 weeks to enhance recruitment and foster partner ships with organizations that organized shorter programs. [↑](#footnote-ref-19)
20. Physical activity levels less than the recommended moderate physical activity of at least 30 minutes per session on 5 or more days per week or vigorous physical activity of at least 20 minutes per session on 3 or more days per week. [↑](#footnote-ref-20)
