# Supplementary material for: Behaviour change interventions to promote health and well-being among older migrants: A systematic review
Source: PLoS One. 2022 Jun 16;17(6):e0269778. doi: 10.1371/journal.pone.0269778 (PMC9202883; doi:10.1371/journal.pone.0269778)
Supplement: S6 Table — (DOCX) [file pone.0269778.s006.docx]

## **S6 Table: Coded BCT per individual intervention**

| **Study** | **Targeted behaviour** | **Bhavioural change techniques** | **Definitely (++)/ possible present (+)** | **Mode of delivery** | **Behaviour change theory/model** |  |
| --- | --- | --- | --- | --- | --- | --- |
|  | **Health behaviour: physical activity, healthy diet, social functioning, blood pressure management, depression management, and health management** |  |  | Individual/ Group |  |  |
| Agurs-Collins, T.D. (1997) [1] | Physical activity Healthy diet | **1 Goals and planning** 1.2 Problem-solving  **3 Social support**  3.1 Social support (unspecified) **4 Shaping knowledge**  4.1 Instruction on how to perform the behaviour **5 Natural consequences** 5.1 Information about health consequences 5.3 Information about social and economic consequences **6 Comparison of behaviour** 6.1 Demonstration of the behaviour 6.2 Social comparison  **8 Repetition and substitution** 8.1 Behavioural practice/ rehearsal  8.2 Behaviour substitution 8.7 Graded tasks **13 Identity**  13.2 Framing/ reframing | ++  ++  ++  ++ ++   ++ +  ++ ++ ++  ++ | Individual and Group | Yes: Social action theory |  |
| Batik, O. (2008) [2] | Physical activity | **1 Goals and palnning**  1.1 Goal- setting (behaviour) 1.2 Problem- solving 1.8 Behavioural contract  **2 Feedback and monitoring**  2.1 Monitoring of behaviour by others without feedback **3 Social support** 3.3 Social support (emotional) **4 Shaping knowledge** 4.1 Instruction on how to perform the behaviour **5 Natural consequences** 5.1 Information about health consequences **9 Comparioson of outcomes**  9.2 Pros and cons | ++ + ++  ++   ++  ++  +  ++ | Individual | No |  |
| Beissner, K. (2012) [3] | Physical activity | **1 Goals and palnning**  1.2 Problem- solving **3 Social support** 3.1 Social support (unspecified) **4 Shaping knowledge**  4.1 Instruction on how to perform the behaviour **6 Comparison of behaviour** 6.1 Demonstration of the behaviour 6.2 Social comparison **8 Repetition and substitution** 8.1 Behavioural practice/ rehearsal 8.3 Habit formation  8.6 Generalization of target behaviour | +  ++  ++  ++ +  ++ ++ ++ | Group | No |  |
| Clark, F. (2012) [4] Juang, C. (2018) [5] | Physical activity | **1 Goals and planning** 1.1 Goal-setting (behaviour) 1.2 Problem-solving  1.8 Behavioural contract **2 Feedback and monitoring** 2.3 Self-monitoring of behaviour **3 Social support** 3.1 Social support (unspecified) **4 Shaping knowledge** 4.1 Instruction on how to perform the behaviour **6 Comparison of behaviour**  6.1 Demonstation how to perform the behaviour 6.2 Social comparison **8 Repetition and substitution**  8.1 Behavioural practice/ rehearsal 8.2 Behaviour substitution  8.3 Habit formation 8.6 Generalization of a target behaviour **9 Comparison of outcomes** 9.1 Credible resource  **12 Antecedents** 12.1 Restructuring the physical environment 12.2 Restructuring the social environment | ++ ++ ++  +  ++  +  +  ++  ++ ++ ++ ++  ++  ++ ++ | Individual and Group | No |  |
| Collins C.C. (2006) [6] | Social functioning | **4 Shaping knowledge**  4.1 Instruction on how to perform the behaviour **5 Natural consequences**  5.1 Information about health consequences **6 Comparison of behaviour** 6.2 Social comparison **8 Repetition and substitution** 8.6 Generalisation of a target behaviour **15 Self-belief**  15.1 Verbal persuasion about capability | ++  ++  +  ++  ++ | Group | No |  |
| Dogra, S. (2015) [7] | Physical activity | **4 Shaping knowledge**  4.1 Instruction on how to perform the behaviour **6 Comparison of behaviour**  6.1 Demonstration of the behaviour **8 Repetition and substitution**  8.1 Behavioural practice/ rehearsal | ++  ++  ++ | Group | No |  |
| Emery-Tilburcio, E. (2017) [8] | Depression management | **1 Goals and planning** 1.2 Problem-solving 1.8 Behavioural contract **2 Feedback and monitoring** 2.1 Monitoring of the behaviour by others without feedback 2.2 Feedback on behaviour **3 Social support** 3.1 Social support (unspecified)  **9 Comparison of outcomes** 9.1 Credible resource **12 Antecedents**  12.5 Adding objects to the environment | + ++  ++  +  ++  ++  ++ | Individual | No |  |
| Fernandez, S. (2008) [9] | Physical activity Diet | **1 Goals and planning** 1.2 Problem-solving  **3 Social support** 3.1 Social support (unspecified) **4 Shaping knowledge** 4.1 Instruction on how to perform the behaviour | ++  ++  ++ | Group | No |  |
| Fried, L.P. (2004) [10] Parisi, J.M. (2015) [11] | Social functioning | **3 Social support** 3.1 Social support (unspecified) **4 Shaping knowledge**  4.1 Instruction on how to perform behaviour **8 Repetition and substitution** 8.1 Behavioural practice/ rehearsal **10 Reward and threat** 10.1 Material incentive | ++  ++  ++  ++ | Individual and group | No |  |
| Geller, K.S. (2012) [12] | Physical activity Healthy diet | **5 Natural consequences**  5.1 Information about health consequences 5.3 Information about social and environmental consequences **6 Comparison of behaviour** 6.2 Social comparison **9 Comparison of outcomes**  9.2 Pros and cons | + +   +  ++ | Group | No |  |
| Goldfinger, J.Z. (2008) [13] | Physical activity Healthy diet | **1 Goals and planning** 1.1 Goal-setting  1.4 Action planning 1.8 Behavioural contract **2 Feedback and monitoring** 2.2 Feedback on behaviour 2.3 Self-monitoring of behaviour **3 Social support**  3.1 Social support (unspecified) **5 Natural consequences**  5.1 Information about health consequences  **6 Comparison of behaviour** 6.2 Social comparison **8 Comparison of behaviour** 8.7 Graded tasks **13 Identity**  13.2 Framing/ reframing | ++ ++ ++  ++ +  +  ++  +  +  ++ | Individual and Group | No |  |
| Hau, C. (2016) [14] | Physical activity Healthy diet | **3 Social support**  3.1 Social support (unspecified) **5 Natural consequences** 5.1 Information about health consequences  **6 Comparison of behaviour** 6.2 Social comparison | ++  +  + | Individual and Group | No |  |
| Holland, S.K. (2005) [15] | Physical activity | **1 Goals and planning** 1.1 Goal-setting  1.2 Problem- solving  1.8 Behavioural contract  **2 Feedback and monitoring** 2.1 Monitoring of behaviour by others without feedback 2.2 Feedback on behaviour 2.3 Self-monitoring of behaviour **3 Social support**  3.1 Social support (unspecified)  **5 Natural consequences**  5.1 Information about health consequences **12 Antecedents** 12.5 Adding objects to the environment **15 Self-belief** 15.1 Verbal persuasion about capability | + + ++  ++  ++ ++  ++  ++  ++  + | Individual | No |  |
| Hooker, S.P. (2011) [16] | Physical activity Social functioning | **1 Goals and planning** 1.1 Goal-setting (behaviour) 1.2 Problem-solving  1.5 Review of goals **2 Feedback and monitoring** 2.2 Feedback on behaviour 2.3 Self-monitoring of behaviour **3 Social support** 3.1 Social support (unspecified) 3.2 Social support (practical) **4 Shaping knowledge**  4.1 Instruction on how to perform the behaviour **5 Natural consequences**  5.1 Information about health consequences  **6 Comparison of behaviour** 6.1 Demonstration of the behaviour 6.2 Social comparison **8 Repetition and substitution**  8.1 Behavioural practice/ rehearsal | ++ ++ ++  ++ ++  ++ ++  ++  ++  ++ ++  ++ | Individual and group | Yes: Social cognitive theory |  |
| Jih, J. (2016) [17] | Physical activity Healthy diet | **5 Natural consequences** 5.1 information about health consequences **12 Antecedents** 12.5 Adding objects to the environment | ++  ++ | Group | No |  |
| Keller, C. (2008) [18] | Physical activity  Healthy diet | **1 Goals and planning** 1.1 Goal-setting (behaviour) 1.4 Action planning 1.8 Behavioural contract **2 Feedback and monitoring** 2.3 Self-monitoring of behaviour **3 Social support** 3.1 Social support (unspecified) 3.2 Social support (practical) **5 Natural consequences** 5.1 Information about health consequences **8 Comparison of behaviour** 8.6 Generalisation of the behaviour 8.7 Graded tasks **10 Reward and threat** 10.1 Material incentive **12 Antecedents** 12.5 Adding objects to the environment | ++ ++ ++  ++  ++ ++  ++  ++ ++  ++  ++ | Individual and group | No |  |
| Kim, B.H. (2013) [19] | Physical activity | **1 Goals and planning** 1.1 Goal-setting behaviour 1.5 Review behaviour goal 1.9 Commitment  **2 Feedback and monitoring**  2.3 Self-monitoring of behaviour **3 Social support**  3.1 Social support (unspecified) 3.2 Social support (practical) **5 Natural consequences** 5.1 Information about health consequences  **7 Associations** 7.1 Prompts/cues **8 Repetition and substitution**  8.2 Behaviour substitution 8.6 Generalization of target behaviour **13 Identity**  13.2 Framing/reframing | ++ ++ ++  ++  ++ ++  ++  ++  ++ ++  ++ | Individual | No |  |
| Kim, K.B. (2014) [20] | Physical activity Healthy diet | **1 Goals and planning** 1.2 Problem-solving 1.3 Goal-setting (outcome) 1.7 Review outcome goal(s) **2 Feedback and monitoring** 2.3 Self-monitoring  2.7 Feedback on outcome(s) of behaviour **3 Social support** 3.1 Social support (unspecified) **4 Shaping knowledge** 4.1 Instruction on how to perform the behaviour  **5 Natural consequences**  5.1 Information about health consequences | ++ ++ +  ++ +  ++  +  + | Individual and group | Yes: Social cognitive theory |  |
| Lu, Y. (2014) [21] | Physical activity Healthy diet | **3 Social support**  3.1 Social support (unspecified)  **5 Natural consequences** 5.1 Information about health consequences **6 Comparison of behaviour** 6.2 Social comparison | ++  ++  + | Individual and Group | No |  |
| Manson, J. (2013) [22, 23] | Physical activity | **4 Shaping knowledge**  4.1 Instruction on how to perform the behaviour **6 Comparison of behaviour** 6.1 Demonstration of the behaviour **8 Repetition and substitution** 8.1 Behavioural practice/ rehearsal | ++  ++  ++ | Group |  |  |
| Melchior, M.A. (2013) [24] | Health management | **1 Goals and planning** 1.1 Goal setting 1.2 Problem-solving **4 Shaping knowledge** 4.1 Instruction on how to perform the behaviour **6 Comparison of behaviour**  6.1 Demonstration how to perform the behaviour 6.2 Social comparison **8 Repetition and substitution**  8.1 Behavioural practice/ rehearsal | ++ ++  ++  ++ +  ++ | Group | No |  |
| Palta, P. (2012) [25] | Blood pressure management | **3 Social support**  3.1 Social support (unspecified) **4 Shaping knowledge** 4.1 Instruction on how to perform the behaviour **6 Comparison of behaviour**  6.1 Demonstation how to perform the behaviour 6.2 Social comparison **8 Repetition and substitution**  8.1 Behavioural practice/rehearsal  **11 Regulation**  11.2 Reduce negative emotions | +  ++  ++ +  ++  ++ | Group | Yes: NIH behaviour change consortium |  |
| Parker, S.J. (2011) [26] | Physical activity | **1 Goals and planning** 1.1 Goal setting (behaviour) 1.2 Problem-solving  1.4 Action planning **4 Shaping knowledge**  4.1 Instruction on how to perform the behaviour **5 Natural consequences** 5.3 Information about social and environmental consequences **6 Comparison of the behaviour** 6.1 Demonstration of the behavior **8 Repetition and substitution** 8.1 Behavioural practice/ rehearsal 8.6 Generalisation of the behaviour **10 Reward and threat** 10.1 Material incentive (behaviour) **12 Antecedents**  12.5 Adding objects to the environment | ++ ++ ++  ++  ++   ++  ++ ++  ++  ++ | Group | No | |
| Qi, B.B. (2001) [27] | Physical activity | **1 Goals and planning** 1.1 Goal setting (behaviour) 1.2 Problem solving **3 Social support** 3.1 Social support (unspecified) **5 Natural consequences** 5.1 Information about health consequences  5.3 Information about social and environmental consequences **6 Comparison of behaviour** 6.2 Social comparison **9 Comparison of outcomes**  9.1 Credible resource  9.2 Pros and cons **10 Reward and threat**  10.1 Material incentive (behaviour) **12 Antecedents**  12.5 Adding objects to the environment | ++ +  ++  ++ ++   +  ++ ++  ++  ++ | Individual and group | Yes: Theory of self-efficacy | |
| Rejeski, W.J. (2014) [28] | Physical activity | **1 Goals and planning** 1.1 Goal setting (behaviour) 1.4 Action planning **2 Feedback and monitoring** 2.3 Self-monitoring of behaviour **4 Shaping knowledge** 4.1 Instruction on how to perform the behaviour **6 Comparison of behaviour** 6.1 Demonstration of the behavior **8 Repetition and substitution** 8.1 Behavioural practice/ rehearsal **9 Comparison of behaviour** 9.2 Pros and cons | ++ ++  ++  ++  ++  ++  ++ | Individual and group | Yes: Social cognitive theory | |
| Resnick, B. (2008) [29] | Physical activity | **1 Goals and planning** 1.1 Goal setting (behaviour) 1.2 Problem-solving **4 Shaping knowledge** 4.1 Instruction on how to perform the behaviour **6 Comparison of behaviour** 6.1 Demonstration of the behavior **8 Repetition and substitution** 8.1 Behavioural practice/ rehearsal 8.6 Generalization of target behaviour **9 Comparison of behaviour** 9.2 Pros and cons  **15 Self- belief**  15.1 Verbal persuasion about capability | ++ ++  ++  ++  ++ ++  ++  ++ | Group | Yes: Theory of self-efficacy | |
| Sin, M.K. (2005) [30] | Physical activity | **4 Shaping knowledge** 4.1 Instruction on how to perform the behaviour **6 Comparison of behaviour** 6.1 Demonstration of the behavior **8 Repetition and substitution** 8.1 Behavioural practice/ rehearsal | ++  ++  ++ | Group | No | |
| Sun, W.Y. (1996) [31] | Physical activity | **4 Shaping knowledge** 4.1 Instruction on how to perform the behaviour **5 Natural consequences** 5.1 Information about health consequences **6 Comparison of behaviour** 6.1 Demonstration of the behavior **8 Repetition and substitution** 8.1 Behavioural practice/ rehearsal **11 Regulation** 11.2 Reduce negative emotions | ++  +  ++  ++  ++ | Group | No | |
| Taylor-Piliae, R.E. (2006) [32, 33] | Physical activity | **4 Shaping knowledge** 4.1 Instruction on how to perform the behaviour **6 Comparison of behaviour** 6.1 Demonstration of the behavior **8 Repetition and substitution** 8.1 Behavioural practice/rehearsal **10 Reward and threat** 10.1 Material incentive (behaviour) | ++  ++  ++  ++ | Group | No | |
| Wilcox, S. (2006) [34] Wilcox, S. (2008) [35] | Physical activity | *AC* **1 Goals and planning** 1.1 Goal setting (behviour) 1.2 Problem solving 1.5 Review goals (behaviour) 1.8 Behavioural contract  **2 Feedback and monitoring**  2.3 Self-monitoring behaviour **3 Social support** 3.1 Social support (unspecified) **9 Comparison of outcome** 9.1 Credible resource **12 Antecedents**  12.5 Adding objects to the environment  *ALED* **3 Social support** 3.1 Social support (unspecified) **4 Shaping knowledge**  4.1 Instruction on how to perform the behaviour **6 Comparison of behaviour**  6.2 Social comparison | ++ + ++ ++  ++  ++  ++  ++   ++  ++  ++ | *AC*: Individual *ALED*: Group | Yes: Social cognitive theory (*AC* and *ALED*) | |
| Wolf, R.L. (2009) [36] | Healthy diet | **1 Goals and planning** 1.1 Goal setting (behaviour) 1.2 Problem solving **3 Social support** 3.1 Social support (unspecified) 3.3 Social support (emotional) **5 Natural consequences**  5.1 Information about health consequences 5.3 Information about social and economic consequences **10 Reward and threat** 10.4 Social reward | ++ +  ++ ++  + +   + | Individual | Yes: Transtheoretical model | |
| Yan, T. (2009) [37] | Physical activity | **1 Goals and planning**  1.1 Goal setting (behaviour)  1.2 Problem solving **3 Social support** 3.2 Social support (unspecified) **4 Shaping knowledge** 4.1 Instruction on how to perform the behaviour **6 Comparison of the behaviour** 6.1 Demonstration of the behaviour **8 Repetition and substitution**  8.1 Behavioural practice/ rehearsal 8.6 Generalisation of a target behaviour 8.7 Graded tasks | ++ +  ++  ++  ++  ++ ++ ++ | Group | Yes: Transtheoretical model | |
| Yan, T. (2009) [38] | Physical activity | **1 Goals and planning** 1.1 Goal setting (behaviour) 1.2 Problem-solving 1.5 Review behaviour goal(s) **2 Feedback and monitoring** 2.2 Feedback on behaviour **3 Social support**  3.1 Social support (unspecified) **4 Shaping knowledge** 4.1 Instruction on how to perform the behaviour **15 Self-belief**  15.1 Verbal persuasion about capability | ++ ++ +  +  ++  ++  ++ | Individual | No | |
| Yeom, H. (2013) [39] | Physical activity Social functioning | **1 Goals and planning**  1.1 Goal setting (behaviour) 1.2 Problem-solving **2 Feedback ad monitoring** 2.3 Self-monitoring of behaviour **3 Social support**  3.1 Social support (unspecified) **4 Shaping knowledge** 4.1 Instruction on how to perform the behaviour **5 Natural consequences**  5.3 Information about social and environmental consequences **6 Comparison of the behaviour** 6.1 Demonstration of the behaviour 6.2 Social comparison **8 Repetition and substitution**  8.1 Behavioural practice/ rehearsal | ++ ++  +  ++  ++  +   ++ +  ++ | Individual and group | Yes: Wellness Motivation Theory | |

1. Agurs-Collins TD, Kumanyika SK, Ten Have TR, Adams-Campbell LL: **A randomized controlled trial of weight reduction and exercise for diabetes management in older African-American subjects**. *Diabetes Care* 1997, **20**(10):1503-1511.

2. Batik O, Phelan EA, Walwick JA, Wang G, LoGerfo JP: **Translating a community-based motivational support program to increase physical activity among older adults with diabetes at community clinics: a pilot study of Physical Activity for a Lifetime of Success (PALS)**. *Prevention Chronic Disease* 2008, **5**(1):A18.

3. Beissner K, Parker SJ, Henderson Jr CR, Pal A, Iannone L, Reid MC: **A cognitive-behavioral plus exercise intervention for older adults with chronic back pain: race/ethnicity effect?** *Journal of Aging and Physical Activity* 2012, **20**(2):246-265.

4. Clark F, Jackson J, Carlson M, Chou CP, Cherry BJ, Jordan-Marsh M, Knight BG, Mandel D, Blanchard J, Granger DA *et al*: **Effectiveness of a lifestyle intervention in promoting the well-being of independently living older people: Results of the Well Elderly 2 Randomised Controlled Trial**. *Journal of Epidemiology and Community Health* 2012, **66**(9):782-790.

5. Juang C, Knight BG, Carlson M, Schepens Niemiec SL, Vigen C, Clark F: **Understanding the Mechanisms of Change in a Lifestyle Intervention for Older Adults**. *Gerontologist* 2018, **58**(2):353-361.

6. Collins CC, Benedict J: **Evaluation of a community-based health promotion program for the elderly: lessons from Seniors CAN**. *American Journal of Health Promotion* 2006, **21**(1):45-48.

7. Dogra S, Shah S, Patel M, Tamim H: **Effectiveness of a Tai Chi intervention for improving functional fitness and general health among ethnically diverse older adults with self-reported arthritis living in low-income neighborhoods: a cohort study**. *Journal of Geriatric Physical Therapy* 2015, **38**(2):71-77.

8. Emery-Tiburcio EE, Mack L, Lattie EG, Lusarreta M, Marquine M, Vail M, Golden R: **Managing Depression among Diverse Older Adults in Primary Care: The BRIGHTEN Program**. *Clinical Gerontologist* 2017, **40**(2):88-96.

9. Fernandez S, Scales KL, Pineiro JM, Schoenthaler AM, Ogedegbe G: **A senior center-based pilot trial of the effect of lifestyle intervention on blood pressure in minority elderly people with hypertension**. *Journal of the American Geriatrics Society* 2008, **56**(10):1860-1866.

10. Fried LP, Carlson MC, Freedman M, Frick KD, Glass TA, Hill J, McGill S, Rebok GW, Seeman T, Tielsch J *et al*: **A social model for health promotion for an aging population: initial evidence on the Experience Corps model**. *Journal of urban health : bulletin of the New York Academy of Medicine* 2004, **81**(1):64-78.

11. Parisi JM, Kuo J, Rebok GW, Xue Q-L, Fried LP, Gruenewald TL, Huang J, Seeman TE, Roth DL, Tanner EK *et al*: **Increases in lifestyle activities as a result of experience Corps® participation**. *Journal of urban health : bulletin of the New York Academy of Medicine* 2015, **92**(1):55-66.

12. Geller KS, Mendoza ID, Timbobolan J, Montjoy HL, Nigg CR: **The Decisional Balance Sheet to Promote Healthy Behavior Among Ethnically Diverse Older Adults**. *Public Health Nurs* 2012, **29**(3):241-246.

13. Goldfinger JZ, Arniella G, Wylie-Rosett J, Horowitz CR: **Project HEAL: Peer education leads to weight loss in harlem**. *Journal of Health Care foor the Poor and Underserved* 2008, **19**(1):180-192.

14. Hau C, Reid KF, Wong KF, Chin RJ, Botto TJ, Eliasziw M, Bermudez OI, Fielding RA: **Collaborative evaluation of the healthy habits program: An effective community intervention to improve mobility and cognition of Chinese older adults living in the U.S**. *Journal Nutrition Health and Aging* 2016, **20**(4):391-397.

15. Holland SK, Greenberg J, Tidwell L, Malone J, Mullan J, Newcomer R: **Community-based health coaching, exercise, and health service utilization**. *Journal of Aging and Health* 2005, **17**(6):697-716.

16. Hooker SP, Harmon B, Burroughs EL, Rheaume CE, Wilcox S: **Exploring the feasibility of a physical activity intervention for midlife African American men**. *Health Education Research* 2011, **26**(4):732-738.

17. Jih J, Le G, Woo K, Tsoh JY, Stewart S, Gildengorin G, Burke A, Wong C, Chan E, Fung LC *et al*: **Educational Interventions to Promote Healthy Nutrition and Physical Activity Among Older Chinese Americans: A Cluster-Randomized Trial**. *American Journal of Public Health* 2016, **106**(6):1092-1098.

18. Keller CS, Cantue A: **Camina por Salud: walking in Mexican-American women**. *Applied Nursing Research* 2008, **21**(2):110-113.

19. Kim BH, Glanz K: **Text messaging to motivate walking in older african americans: A randomized controlled trial**. *American Journal of Preventive Medicine* 2013, **44**(1):71-75.

20. Kim KB, Han HR, Huh B, Nguyen T, Lee H, Kim MT: **The effect of a community-based self-help multimodal behavioral intervention in Korean American seniors with high blood pressure**. *American Journal of Hypertension* 2014, **27**(9):1199-1208.

21. Lu Y, Dipierro M, Chen L, Chin R, Fava M, Yeung A: **The evaluation of a culturally appropriate, community-based lifestyle intervention program for elderly Chinese immigrants with chronic diseases: a pilot study**. *Journal of public health (Oxford, England)* 2014, **36**(1):149-155.

22. Manson J, Ritvo P, Ardern C, Weir P, Baker J, Jamnik V, Tamim H: **Tai Chi's Effects on Health-Related Fitness of Low-Income Older Adults**. *Canadian Journal on Aging* 2013, **32**(3):270-277.

23. Manson J, Rotondi M, Jamnik V, Ardern C, Tamim H: **Effect of tai chi on musculoskeletal health-related fitness and self-reported physical health changes in low income, multiple ethnicity mid to older adults**. *BMC Geriatrics* 2013, **13**:114.

24. Melchior MA, Seff LR, Bastida E, Albatineh AN, Page TF, Palmer RC: **Intermediate outcomes of a chronic disease self-management program for Spanish-speaking older adults in South Florida, 2008-2010**. *Prevention Chronic Disease* 2013, **10**:E146.

25. Palta P, Page G, Piferi RL, Gill JM, Hayat MJ, Connolly AB, Szanton SL: **Evaluation of a mindfulness-based intervention program to decrease blood pressure in low-income African-American older adults**. *Journal of urban health : bulletin of the New York Academy of Medicine* 2012, **89**(2):308-316.

26. Parker SJ, Vasquez R, Chen EK, Henderson CR, Jr., Pillemer K, Robbins L, Reid MC: **A comparison of the arthritis foundation self-help program across three race/ethnicity groups**. *Ethnicity and Disease* 2011, **21**(4):444-450.

27. Qi BB, Resnick B, Smeltzer SC, Bausell B: **Self-efficacy program to prevent osteoporosis among Chinese immigrants: a randomized controlled trial**. *Nursing research* 2011, **60**(6):393-404.

28. Rejeski WJ, Spring B, Domanchuk K, Tao H, Tian L, Zhao L, McDermott MM: **A group-mediated, home-based physical activity intervention for patients with peripheral artery disease: effects on social and psychological function**. *J Transl Med* 2014, **12**:29-29.

29. Resnick B, Luisi D, Vogel A: **Testing the Senior Exercise Self-efficacy Project (SESEP) for use with Urban dwelling minority older adults**. *Public Health Nurs* 2008, **25**(3):221-234.

30. Sin MK, Belza B, LoGerfo J, Cunningham S: **Evaluation of a community-based exercise program for elderly Korean immigrants**. *Public Health Nurs* 2005, **22**(5):407-413.

31. Sun WY, Dosch M, Gilmore GD, Pemberton W, Scarseth T: **Effects of a Tai Chi Chuan program on Hmong American older adults**. *Educational Gerontology* 1996, **22**(2):161-167.

32. Taylor-Piliae RE, Haskell WL, Sivarajan Froelicher E: **Hemodynamic responses to a community-based Tai Chi exercise intervention in ethnic Chinese adults with cardiovascular disease risk factors**. *European Journal of Cardiovascular Nursing: journal of the Working Group on Cardiovascular Nursing of the European Society of Cardiology* 2006, **5**(2):165-174.

33. Taylor-Piliae RE, Haskell WL, Waters CM, Froelicher ES: **Change in perceived psychosocial status following a 12-week Tai Chi exercise programme**. *Journal of Advanced Nursing* 2006, **54**(3):313-329.

34. Wilcox S, Dowda M, Griffin SF, Rheaume C, Ory MG, Leviton L, King AC, Dunn A, Buchner DM, Bazzarre T *et al*: **Results of the first year of active for life: translation of 2 evidence-based physical activity programs for older adults into community settings**. *American Journal of Public Health* 2006, **96**(7):1201-1209.

35. Wilcox S, Dowda M, Leviton LC, Bartlett-Prescott J, Bazzarre T, Campbell-Voytal K, Carpenter RA, Castro CM, Dowdy D, Dunn AL *et al*: **Active for Life. Final Results from the Translation of Two Physical Activity Programs**. *American Journal of Preventive Medicine* 2008, **35**(4):340-351.

36. Wolf RL, Lepore SJ, Vandergrift JL, Basch CE, Yaroch AL: **Tailored telephone education to promote awareness and adoption of fruit and vegetable recommendations among urban and mostly immigrant black men: a randomized controlled trial**. *Preventive Medicine* 2009, **48**(1):32-38.

37. Yan T, Wilber KH, Aguirre R, Trejo L: **Do sedentary older adults benefit from community-based exercise? results from the active start program**. *Gerontologist* 2009, **49**(6):847-855.

38. Yan T, Wilber KH, Wieckowski J, Simmons WJ: **Results from the healthy moves for aging well program: Changes of the health outcomes**. *Home Health Care Services Quarterly* 2009, **28**(2-3):100-111.

39. Yeom HA, Fleury J: **A Motivational Physical Activity Intervention for Improving Mobility in Older Korean Americans**. *Western Journal of Nursing Research* 2014, **36**(6):713-731.
