# Supplementary material for: Behaviour change interventions to promote health and well-being among older migrants: A systematic review
Source: PLoS One. 2022 Jun 16;17(6):e0269778. doi: 10.1371/journal.pone.0269778 (PMC9202883; doi:10.1371/journal.pone.0269778)
Supplement: S7 Table — (DOCX) [file pone.0269778.s007.docx]

## **S7 Table: Intervention effectiveness involving assessment ≥ 3 months post baseline by outcome clusters ***

| **Anthropometrics outcomes** | | | | | |
| --- | --- | --- | --- | --- | --- |
| Targeted behaviour |  | Evidence of effectiveness (n=10) | No evidence of effectiveness (n=2) | All (n=12) | Index of potential** |
|  | Physical activity | 5 | 2 | 8 | 75% |
|  | Physical activity and healthy diet | 5 |  | 5 | 100% |
|  | Physical activity and social functioning |  | 1 |  |  |
| Intervention functions^#^ |  |  |  |  |  |
|  | **Education** | **4** | **2** | **6** | **67%** |
|  | **Enablement** | **3** | **2** | **5** | **60%** |
|  | **Modeling** | **5** |  | **5** | **100%** |
|  | **Training** | **5** |  | **5** | **100%** |
| Cultural adaption^¥^ |  | 7 |  | 7 | 100% |
|  | Linguistic strategy | 6 |  | 6 | 100% |
| BCT code | BCT label |  |  |  |  |
| 1.1 | Goal-setting (behaviour) | 2 | 2 | 4 | 50% |
| **1.2** | **Problem-solving** | **3** | **2** | **5** | **60%** |
| **2.3** | **Self-monitoring of behaviour** | **3** | **1** | **4** | **75%** |
| **3.1** | **Social support (unspecified)** | **6** | **1** | **7** | **86%** |
| **4.1** | **Instruction on how to perform the behaviour** | **8** | **1** | **9** | **89%** |
| **5.1** | **Information about health consequences** | **5** | **2** | **7** | **71%** |
| **8.1** | **Behavioural practice/ rehearsal** | **5** |  | **5** | **100%** |
| **Behaviour outcomes** | | | | | |
| Targeted behaviour |  | Evidence of effectiveness (n=9) | No evidence of effectiveness (n=1) | All (n=10) | Index of potential** |
|  | Physical activity | 4 |  | 4 | 100% |
|  | Physical activity and healthy diet | 4 | 2 | 6 | 67% |
| Intervention functions^#^ |  |  |  |  |  |
|  | **Education** | **5** | **1** | **6** | **83%** |
|  | **Enablement** | **5** | **2** | **7** | **71%** |
|  | **Persuasion** | **4** | **1** | **5** | **80%** |
| Cultural adaption^¥^ |  | 5 | 1 | 6 | 83% |
|  | Linguistic strategy | 3 | 1 | 4 | 75% |
|  | Sociocultural strategy | 3 | 1 | 4 | 75% |
| BCT code | BCT label |  |  |  |  |
| **1.1** | **Goal-setting (behaviour)** | **7** |  |  | **100%** |
| **1.2** | **Problem-solving** | **6** |  | **6** | **100%** |
| **2.3** | **Self-monitoring of behaviour** | **4** |  | **4** | **100%** |
| **3.1** | **Social support (unspecified)** | **7** | **1** | **8** | **88%** |
| **4.1** | **Instruction on how to perform the behaviour** | **5** |  | **5** | **100%** |
| **5.1** | **Information about health consequences** | **7** | **1** | **8** | **88%** |
| **Physical functioning outcomes** | | | | | |
| Targeted behaviour |  | Evidence of effectiveness (n=16) | No evidence of effectiveness (n=1) | All (n=17) | Index of potential** |
|  | Physical activity | 13 | 1 | 14 | 93% |
| Intervention functions^#^ |  |  |  |  |  |
|  | **Education** | **6** |  | **6** | **100%** |
|  | **Enablement** | **8** | **1** | **9** | **89%** |
|  | **Environmental restructuring** | **2** |  | **2** | **100%** |
|  | **Modeling** | **8** |  | **8** | **100%** |
|  | **Persuasion** | **4** |  | **4** | **100%** |
|  | **Training** | **5** |  | **5** | **100%** |
| Cultural adaption^¥^ |  | 12 | 1 | 13 | 92% |
|  | Linguistic strategy | 10 | 1 | 11 | 91% |
|  | Sociocultural strategy | 6 |  | 6 | 100% |
| BCT code | BCT label |  |  |  |  |
| **1.1** | **Goal-setting (behaviour)** | **7** |  | **7** | **100%** |
| **1.2** | **Problem-solving** | **7** |  | **7** | **100%** |
| **2.3** | **Self-monitoring of behaviour** | **4** |  | **4** | **100%** |
| **3.1** | **Social support (unspecified)** | **6** | **1** | **7** | **86%** |
| **4.1** | **Instruction on how to perform the behaviour** | **12** |  | **12** | **100%** |
| **5.1** | **Information about health consequences** | **3** | **1** | **4** | **75%** |
| **6.1** | **Demonstration how to perform the behaviour** | **11** |  | **11** | **100%** |
| **8.1** | **Behavioural practice/ rehearsal** | **12** |  | **12** | **100%** |
| **8.6** | **Generalization of a target behaviour** | **4** |  | **4** | **100%** |
| **Mental health and cognitive functioning** | | | | | |
| Targeted behaviour |  | Evidence of effectiveness (n=10) | No evidence of effectiveness (n=3) | All (n=13) | Index of potential** |
|  | Physical activity | 7 | 4 | 11 | 63% |
| Intervention functions^#^ |  |  |  |  |  |
|  | **Education** | **4** | **1** | **5** | **80%** |
|  | **Enablement** | **6** | **3** | **9** | **67%** |
|  | Persuasion | 1 | 3 | 4 | 25% |
|  | **Training** | **4** | **2** | **6** | **67%** |
| Cultural adaption^¥^ |  | 9 | 1 | 10 | 90% |
|  | Linguistic strategy | 9 | 1 | 10 | 90% |
|  | Sociocultural strategy | 4 |  | 4 | 100% |
| BCT code | BCT label |  |  |  |  |
| **1.1** | **Goal-setting (behaviour)** | **3** | **2** | **5** | **60%** |
| **1.2** | **Problem-solving** | **5** | **2** | **7** | **71%** |
| **3.1** | **Social support (unspecified)** | **6** | **3** | **9** | **67%** |
| **4.1** | **Instruction on how to perform the behaviour** | **7** | **2** | **9** | **78%** |
| **5.1** | **Information about health consequences** | **3** | **1** | **4** | **75%** |
| **6.1** | **Demonstration how to perform the behaviour** | **5** |  | **5** | **100%** |
| **8.1** | **Behavioural practice/ rehearsal** | **5** | **1** | **6** | **83%** |
| **12.5** | **Adding objects to the environment** | **3** | **1** | **4** | **75%** |
| **Social functioning** | | | | | |
| Targeted behaviour |  | Evidence of effectiveness (n=6) | No evidence of effectiveness (n=1) | All (n=7) | Index of potential** |
|  | Physical activity | 4 | 1 | 8 | 80% |
| Intervention functions^#^ |  |  |  |  |  |
|  | **Persuasion** | **3** | **1** | **4** | **75%** |
|  | **Training** | **4** |  | **4** | **100%** |
| Cultural adaption^¥^ |  | 4 | 1 | 5 | 80% |
| BCT code | BCT label |  |  |  |  |
| **4.1** | **Instruction on how to perform the behaviour** | **6** | **1** | **7** | **86%** |
| **6.1** | **Demonstration how to perform the behaviour** | **4** | **1** | **5** | **80%** |
| **8.1** | **Behavioural practice/ rehearsal** | **5** | **1** | **6** | **83%** |
| **Generic health and well-being** | | | | | |
| Targeted behaviour |  | Evidence of effectiveness (n=9) | No evidence of effectiveness (n=4) | All (n=13) | Index of potential** |
|  | Physical activity | 8 | 3 | 11 | 73% |
| Intervention functions^#^ |  |  |  |  |  |
|  | **Education** | **4** | **3** | **7** | **57%** |
|  | Enablement | 4 | 4 | 8 | 50% |
|  | **Modeling** | **4** | **1** | **5** | **80%** |
|  | **Persuasion** | **2** | **3** | **5** | **40%** |
|  | **Training** | **4** |  | **4** | **100%** |
| Cultural adaption^¥^ |  | 6 | 3 | 9 | 67% |
|  | Linguistic strategy | 5 | 2 | 7 | 71% |
| BCT code | BCT label |  |  |  |  |
| 1.1 | Goal-setting (behaviour) | 3 | 3 | 6 | 50% |
| 1.2 | Problem-solving | 3 | 3 | 6 | 50% |
| 1.8 | Behavioural contract | 1 | 3 | 4 | 25% |
| 2.2 | Feedback on behaviour | 1 | 3 | 4 | 25% |
| 3.1 | Social support (unspecified) | 3 | 3 | 6 | 50% |
| **4.1** | **Instruction on how to perform the behaviour** | **9** | **1** | **10** | **90%** |
| 5.1 | Information about health consequences | 2 | 2 | 4 | 50% |
| **6.1** | **Demonstration how to perform the behaviour** | **6** | **1** | **7** | **86%** |
| **6.2** | **Social comparison** | **3** | **1** | **4** | **75%** |
| **8.1** | **Behavioural practice/ rehearsal** | **6** | **1** | **7** | **86%** |
| **8.6** | **Generalization of a target behaviour** | **4** | **1** | **5** | **80%** |
| 15.1 | Verbal persuasion about capability | 2 | 2 | 4 | 50% |

*Only characteristics identified in at least four interventions within each cluster are reported for that cluster.
** Index of potential refers to the percentage of studies, of all those featuring the focal intervention characteristic, found to show evidence of potential effectiveness on at least one variable within the relevant outcome cluster. Rows in bold denote components found to show promise (index potential >50%).

^#^Definitions of intervention functions: education, ‘increasing knowledge or understanding’; enablement, ‘increasing means/reducing barriers to increase capability (beyond education and training) or opportunity (beyond environmental restructuring)’; environmental restructuring, ‘changing the physical or social context’; modelling, ‘providing an example for people to aspire to or imitate’; persuasion, ‘using communication to induce positive or negative feelings or stimulate action’; training, ‘imparting skills’ (ref. [1], p. 7).
^¥^ Definitions of cultural adaption strategies: linguistic, improving programme/materials accessibility by providing them in the dominant or native language of the target population; socio-cultural, discussing health-related issues in the context of broader social and/or cultural values and characteristics of the target population (ref. [2], p. 135-136).

1. Michie S, van Stralen M, West R: **The behaviour change wheel: a new method for characterising and designing behaviour change interventions**. *Implementation Science* 2011, **6**:42.

2. Kreuter MW, Lukwago SN, Bucholtz DC, Clark EM, Sanders-Thompson V: **Achieving Cultural Appropriateness in Health Promotion Programs: Targeted and Tailored Approaches**. *Health Education & Behavior* 2003, **30**(2):133-146.
