# Supplementary material for: Behaviour change interventions to promote health and well-being among older migrants: A systematic review
Source: PLoS One. 2022 Jun 16;17(6):e0269778. doi: 10.1371/journal.pone.0269778 (PMC9202883; doi:10.1371/journal.pone.0269778)
Supplement: S8 Table — (DOCX) [file pone.0269778.s008.docx]

**S8 Appendix. Summary of study characteristics**

| **Author (year) Location** | **Study design** | | | **Sample characteristics** | | **Level of evidence** | | **Susceptibility to bias** | | |
| --- | --- | --- | --- | --- | --- | --- | --- | --- | --- | --- |
|  | **RCT** | **Pre-post design** | **Quasi-experiment** | **Sample size** | **Gender  (≥ 50 % female)** | **Level 1** | **Level 2** | **Low** | **Moderate** | **High** |
| Agurs-Collins, T.D. (1997) [1] Washington, D.C., United States | x |  |  | IG n=32, CG n=32 | x | x |  |  |  | x |
| Batik, O. (2008) [2] South Seattle, United States | x |  |  | IG n=135, CG n=170 | x | x |  |  |  | x |
| Beissner, K. (2012) [3] New York City, United States |  | x |  | IG n=69 | x |  | x | x |  |  |
| Clark, F. (2012) [4]  Juang, C. (2018) [5]  Los Angeles, United States | x |  |  | IG n=232, CG n=228 | x | x |  |  |  | x |
| Collins C.C. (2006) [6] Nevada, United States |  | x |  | IG n=339 | x |  | x |  | x |  |
| Dogra, S. (2015) [7] Greater Toronto Area, Canada |  | x |  | IG n=201 | x |  | x |  | x |  |
| Emery-Tilburcio, E. (2017) [8] Cook County, United States |  | x |  | IG n=131 | x |  | x |  | x |  |
| Fernandez, S. (2008) [9] New York City, United States | x |  |  | IG n=35, CG n=30 | x | x |  |  |  | x |
| Fried, L.P. (2004) [10] Baltimore, United States | x |  |  | IG n=70, CG n=58 | x | x |  |  | x |  |
| Geller, K.S. (2012) [11] Hawaii | x |  |  | Physical activity n=12 Fruit and Vegetable n=9 | x | x |  |  |  | x |
| Goldfinger, J.Z. (2008) [12] Harlem, United States |  | x |  | IG n= 26 | x |  | x |  | x |  |
| Hau, C. (2016)[13] Boston, United States |  | x |  | IG n=50 | N.m |  | x | x |  |  |
| Holland, S.K. (2005) [14] California, United States | x |  |  | IG n=255, CG n=249 | x | x |  | x |  |  |
| Hooker, S.P. (2011) [15]  Columbia, United Sates |  |  | x | IG n=25 |  |  | x |  | x |  |
| Jih, J. (2016) [16] San Francisco, United States | x |  |  | IG n=365, CG n=360 | x | x |  | x |  |  |
| Keller, C. (2008) [17] United States | x |  |  | Group I N = 11 Group II N = 7 | x | x |  |  | x |  |
| Kim, B.H. (2013) [18] United States | x |  |  | IG n=26, CG n=15 | x | x |  |  |  | x |
| Kim, K.B. (2014) [19] Baltimore, United States | x |  |  | IG n=184, CG n=185 | x | x |  | x |  |  |
| Lai et al. (2020) [20]  Canada | x |  |  | IG n=30, CG n=30 | x | x |  |  |  | x |
| Lu, Y. (2014) [21] Boston, United States |  | x |  | IG n=99 | x |  | x |  | x |  |
| Manson, J. (2013) [22] Toronto, Canada |  | x |  | IG n=78 | x |  | x |  | x |  |
| Manson, J. (2013) [23] Greater Toronto Area, Canada |  | x |  | IG n=209 | x |  | x |  | x |  |
| Melchior, M.A. (2013) [24] South Florida, United States |  | x |  | IG n=682 | x |  | x |  | x |  |
| Palta, P. (2012) [25] Baltimore, United States | x |  |  | IG n= 12, CG n= 8 | x | x |  | x |  |  |
| Parisi, J.M. (2015) [26] Baltimore, United States | x |  |  | IG n=352, CG n=350 | x | x |  | x |  |  |
| Parker, S.J. (2011) [28] New York City, United States |  | x |  | IG n=112 | x |  | x | x |  |  |
| Piedra et al. [29] (2017)  Los Angles, United States | x |  |  | IG n=279, CG n=292 | x | x |  |  |  | x |
| Qi, B.B. (2001) [30]  United States | x |  |  | IG n=4, CG n=41 | x | x |  | x |  |  |
| Reijneveld et al. [31] (2003)  the Netherlands | x |  |  | Age: 54.8 (7.5) | x | x |  |  |  | x |
| Rejeski, W.J. (2014) [32] Chicago area, United States | x |  |  | IG n=88, CG n=90 | x | x |  |  |  | x |
| Resnick, B. (2008) [33] New York City, United States | x |  |  | IG n=100, CG n=66 | x | x |  | x |  |  |
| Sin, M.K. (2005) [34] Seattle, United States |  | x |  | IG n=13 | x |  | x |  | x |  |
| Skelly et al. [35] (2009) United States | x |  |  | Age: 67 | x | x |  |  |  | x |
| Sun, W.Y. (1996) [36] La Crosse County, United States | x |  |  | IG n=10, CG n=10 | x | x |  | x |  |  |
| Taylor-Piliae, R.E. (2006) [37, 38] San Francisco Bay Area, United States |  |  | x | IG n=39 | x |  | x |  | x |  |
| Wilcox, S. (2006) [39] United States |  |  | x | IG *AC* n=384 *ALED* n=454 |  |  | x | x |  |  |
| Wilcox, S. (2008) [40] United States |  |  | x | IG *AC* Year 1 n=384 Year 2 n=1136 Year 3 n=982  *ALED* Year 1 n=454 Year 2 n=1433 Year 3 n=1501 | x |  | x | x |  |  |
| Wolf, R.L. (2009) [41] New York City, United States | x |  |  | IG n=240, CG n=239 | N.m. | x |  | x |  |  |
| Yan, T. (2009)[42]  Los Angles, United States |  |  | x | IG n=151, CG n=57 Female 82.21% | x |  | x | x |  |  |
| Yan, T. (2009) [43] California, United States |  | x |  | IG n=518 | x |  | x | x |  |  |
| Yeom, H. (2013) [44] United States |  |  | x | IG n=33, CG n=31 |  |  | x | x |  |  |
| **Total** | 22 | 13 | 6 |  | 36 | 22 | 19 | 17 | 13 | 11 |
